# UHPLC-QTOF-ESI-MS/MS, SNAP-MS Identification, In Silico Prediction of Pharmacokinetic Properties of Constituents from the Stem Bark of *Holarrhena floribunda* (G. Don) T. Durand and Schinz (Apocynaceae)

**DOI:** 10.3390/biom15101415

**Published:** 2025-10-04

**Authors:** Franck Landry Djila Possi, Mc Jesus Kinyok, Joseph Eric Mbasso Tameko, Bel Youssouf G. Mountessou, Johanne Kevine Jumeta Dongmo, Mariscal Brice Tchatat Tali, Appolinaire Kene Dongmo, Fabrice Fekam Boyom, Jean Jules Kezetas Bankeu, Norbert Sewald, Jean Rodolphe Chouna, Bruno Ndjakou Lenta

**Affiliations:** 1 Department of Organic Chemistry, Faculty of Science, University of Yaoundé 1, Yaoundé P.O. Box 812, Cameroon; possidjila@yahoo.com (F.L.D.P.); jumetakevine@yahoo.fr (J.K.J.D.); 2 Department of Chemistry, Higher Teacher Training College, University of Yaoundé 1, Yaoundé P.O. Box 47, Cameroon; jesus-kinyok.mc@univ-yaounde1.cm (M.J.K.); tamekombasso@yahoo.fr (J.E.M.T.); mountessou@yahoo.com (B.Y.G.M.); bk_jeanjules@yahoo.fr (J.J.K.B.); bruno.lenta_ndjakou@uni-bielefeld.de (B.N.L.); 3 Department of Biochemistry, Faculty of Science, University of Yaoundé 1, Yaoundé P.O. Box 812, Cameroon; b.tchatat@yahoo.com (M.B.T.T.); fabrice.boyom@fulbrightmail.org (F.F.B.); 4 Organic and Bioorganic Chemistry, Faculty of Chemistry, Bielefeld University, P.O. Box 100131, D-33501 Bielefeld, Germany; norbert.sewald@uni-bielefeld.de; 5 Department of Chemistry, Faculty of Science, University of Dschang, Dschang P.O. Box 67, Cameroon; jean.chouna@univ-dschang.org

**Keywords:** Apocynaceae, *Holarrhena floribunda*, UHPLC-QTOF-ESI-MS/MS identification, SNAP-MS, constituents, in silico prediction, pharmacokinetic properties

## Abstract

The present work reports the bioguided isolation of constituents from the ethanol extract of *Holarrhena floribunda* stem bark, their identification by UHPLC-ESI-QTOF-MS/MS identification, and the in silico prediction of the pharmacokinetic and toxicity parameters. The crude extract, along with its *n*-hexane and alkaloid-rich fractions, displayed moderate to good antiplasmodial activity in vitro against chloroquine-sensitive (3D7) and multidrug-resistant (Dd2) strains of *Plasmodium falciparum*, with IC_50_ values ranging from 6.54 to 43.54 µg/mL. Seventeen steroidal alkaloids (**1**–**17**) were identified in the most active fraction using UHPLC-ESI-QTOF-MS/MS, based on their fragmentation patterns and analysis with the Structural Similarity Network Annotation Platform for Mass Spectrometry (SNAP-MS). Furthermore, bioguided isolation of the ethanol extract yielded twenty-one compounds (**3**, **5**, **10**, **14**–**16**, **18**–**31**), whose structures were elucidated by spectroscopic methods. Among them, compounds **5**, **14**, and **27** showed the highest potency against the two strains of *P. falciparum*, with IC_50_ values between 25.97 and 55.78 µM. In addition, the in silico prediction of pharmacokinetic parameters and drug-likeness using the SwissADME web tool indicated that most of the evaluated compounds (**1**, **3**–**5**, and **14**–**16**) complied with Lipinski’s rule of five.

## 1. Introduction

Malaria remains a global health challenge, mainly in sub-Saharan Africa, where this disease continues to kill thousands of people [1]. Hence, according to the 2024 World Health Organization (WHO) report, there were approximately 269 million cases of malaria affecting people from sub-Saharan countries, accounting for 94% of worldwide cases in 2023 [2]. In sub-Saharan Africa, this disease constitutes one of the major threats [3], especially in Cameroon, where about 3 million cases and more than 3800 deaths were reported in 2021 [4]. The first-line treatment options for this disease are based on the use of artemisinin-based combination therapies (ACTs), as recommended by WHO [5]. In addition, there are two existing malaria vaccines approved by the World Health Organization. Unfortunately, these vaccines have not shown satisfactory effectiveness (only 40% for RTS,S and around 75% for Matrix 21). These concerns, the spreading malaria parasite resistance to the first-line therapies in endemic settings, and the limited effectiveness of available vaccines highlight the urgent need for improved solutions to the malaria plague. Medicinal plants have historically played a central role in the treatment of human diseases, as evidenced by the success stories of quinine and artemisinin. The sub-Saharan African region carries the lion’s share of malaria burden, but is endowed with one of the richest plant biodiversity, from which novel chemical scaffolds can be discovered and developed as alternatives to the current chemotherapeutic agents. In this line, *Holarrhena floribunda,* a plant widely used in West and Central Africa for treating multiple diseases including malaria, dysentery, diarrhea, infertility, abdominal pains, nausea, indigestion, and diabetes [6,7,8] appears as a realistic starting material to search for novel antimalarial pharmacophores. Previous biological and chemical studies on this plant have revealed antibacterial [7], antiplasmodial [8,9,10], antitrypanosomal, cytotoxic [9,10], antioxidant [11], and antimycobacterial [12] activities and led to the isolation of triterpenoids [8], steroidal alkaloids [10], flavonoids [13], and diterpenoids [14].

Owing to the ethnopharmacological profile of *H. floribunda* and the sparse reports on the experimental validation of the antiplasmodial properties of its constituents [8,12], we undertook an antiplasmodial-guided investigation of *H. floribunda* ethanolic extract*,* followed by in silico prediction of the drug-likeness properties of selected constituents. Therefore, the present study reports the identification of seventeen steroidal alkaloids (**1**–**17**) from the alkaloid-rich fraction through Structural Similarity Network Annotation Platform for Mass Spectrometry (SNAP-MS), in combination with fragmentation patterns and data from the literature. In addition, the in silico prediction of pharmacokinetic parameters and target prediction of compounds (**1**, **3**, **5**, **10**, **11** and, **14**–**16**) identified from the most active fraction are also reported herein. Also, the isolation of twenty-one compounds (**3**, **5**, **11**, **14**, **16**, and **18**–**32**), as well as the in vitro antiplasmodial and cytotoxic activities of the extract, fractions, and compounds from the stem bark of this plant, are also discussed herein.

## 2. Materials and Methods

### 2.1. Reagent and Materials

For this study, ethanol was used as an extraction solvent for the plant material. *n*-hexane, methylene chloride and *n*-butanol were used for partitioning. *n*-Hexane, cyclohexane, ethyl acetate and methanol were used as pure or binary mixtures at different polarities for the purification of compounds. Column chromatography was carried out with silica gel 230–400 mesh (Merck, Darmstadt, Germany), 70–230 mesh (Merck, Darmstadt, Germany) or Sephadex LH-20 (Sigma-Aldrich, Munich, Germany) as stationary phase. Thin-layer chromatography (TLC) was performed on Merck pre-coated silica gel (60 F254) aluminum foil (Merck, Darmstadt, Germany), and spots were visualized by inspection under a UV lamp operating at 254 or 365 nm and then by spraying with diluted sulfuric acid (20%) or with Dragendorff reagent before heating at about 100 °C. High-resolution mass spectra were recorded using a QTOF Compact Spectrometer (Bruker Corporation, Bremen, Germany). The ^1^H and ^13^C NMR spectra were recorded on Bruker Avance 600 (^1^H NMR, 600 MHz and ^13^C NMR, 150 MHz). Chemical shifts were reported in *δ* (ppm) using deuterated solvent as an internal standard, while coupling constants (*J*) were measured in Hz.

### 2.2. UHPLC-QTOF-ESI-MS/MS Analyses

High-resolution mass spectra were obtained with a QTOF Spectrometer (Bruker Corporation, Germany) equipped with an electrospray (ESI) source. The spectrometer was operated in the positive mode (mass range: 100–1500, with a scan rate of 1.00 Hz) with automatic gain control to provide high-accuracy mass measurements within 0.40 ppm deviation, using Na formate as calibrant. The following parameters were used for experiments: spray voltage of 4.5 kV and capillary temperature of 220 °C. Nitrogen was used as sheath gas (9 L/min). The MS/MS analyses were operated using a collision-induced dissociation method (CID) with collision energy of 45 eV. The spectrometer was coupled to an Ultimate 3000 (Thermo Fisher, Waltham, MA, USA) UHPLC system equipped with a LC-pump, a Diode Array Detector (DAD) (λm: 190–600 nm), an auto sampler (injection volume 100 μL) and an Accucore C-18 Reversed phase column (50 × 2.1 mm, 2.6 µm, 150 Å) in an oven temperature of 35 °C. The mobile phases consisted of water + 0.1% formic acid (A) and acetonitrile + 0.1% formic acid (B). Separation was carried out at 35 °C, at a flow rate of 0.4 mL/min, used with the following multistep linear gradient: 5% B isocratic for 10 min, 5–60% B for 22 min, 60% B isocratic for 3 min, 60–95% B for 2 min, 95% B isocratic for 1 min, 95–5% B for 1 min. The initial conditions were maintained for 1 min.

### 2.3. In Vitro Antiplasmodial Activity

#### *Plasmodium falciparum* Culture and Growth Inhibition Assay

*P. falciparum* 3D7 (Chloroquine-sensitive) and Dd2 (multidrug-resistant) strains were obtained from the Biodefense and Emerging Infections (BEI) Research Resources (Manassas, VA, USA) and were maintained using a slightly modified Trager and Jensen method (2005) [15]. In fact, parasites were cultured in fresh O^+^ human red blood cells at 3% (*v*/*v*) hematocrit in RPMI 1640 culture media containing glutamax NaHCO_3_ (Gibco, Paisley, UK), supplemented with 25 mM HEPES (Gibco, UK), 1X hypoxanthine (Gibco, Grand Island, NY, USA), 20 µg/mL gentamicin (Gibco, Shanghai, China), and 0.5% Albumax II (Gibco, Invitrogen, New York, NY, USA). Prior to the drug efficacy test, parasites were synchronized at the ring stage by sorbitol treatment and cultured through one cycle (48 h).

Stock solutions (100 mg/mL (Extracts and fractions) and 10 mg/mL (compounds) were prepared in 100% DMSO and serially diluted (5-fold) in complete RPMI 1640 prior to activity studies. The diluted intermediate solutions were co-cultured with parasite cultures (1% parasitemia and 2% hematocrit) in 96-well plates to a final drug concentration ranging from 50 to 0.08 µM for compounds and 100 to 0.16 µg/mL for extracts and fractions. The final dimethylsulfoxide (DMSO) concentration per 100 μL culture per well was less than 0.5% for all the tested samples. Chloroquine and artemisinin at a range of 1–0.0016 µM each were used as a negative growth control (positive test control), while the solvent-treated culture (<0.5% DMSO) was used as a positive growth control (negative test control). Following 72 h of incubation at 37 °C in a 5% CO_2_ incubator, parasite growth was assessed by a SYBR green I-based DNA quantification assay. A total of 100 μL of SYBR Green I buffer {[6 μL of 10,000 × SYBR Green I (Invitrogen) + 600 µL of red blood cells lysis buffer [Tris (25 mM; pH 7.5)] + 360 µL of EDTA (7.5 mM) + 19.2 µL of parasites lysis solution [saponin (0.012%; *w*/*v*)] and 28.8 µL of Triton X − 100 (0.08%; *v*/*v*)]} was added to each well and incubated in the dark at 37 °C for 1 h. Fluorescence was measured using an infinite M200 plate reader with excitation and emission wavelengths at 485 and 538 nm, respectively. Mean half-maximal inhibitory concentrations (IC_50_ values) were derived by plotting percent growth against log drug concentration and fitting the response data to a variable slope sigmoidal curve function using GraphPad Prism v8.0. The antiplasmodial assays were performed in triplicate.

### 2.4. Plant Material

The stem bark of *H. floribunda* was collected in Kon-Yambetta (GPS coordinate: latitude: 4.83333, longitude: 11.06674°49′60″ N, 11°4′0″ E) in Mbam et Inoubou Division, Centre region of Cameroon, in April 2022. The sample was identified by comparison with an available sample recorded under the voucher specimen N° 49821 HNC by Mr. Nana Victor, a botanist of the National Herbarium of Cameroon.

#### Extraction and Isolation

The stem bark of *H. floribunda* was chopped into small pieces, air dried and ground to give 4.5 kg of fine powder. The powder was macerated at room temperature (about 28 °C) using ethanol and freed from solvent to afford 155.2 g of crude extract. The crude extract (145.6 g) was dissolved in 500 mL of acidified water (HCl, 3 < pH < 4) and subjected to the liquid–liquid partition, using *n*-hexane (1 L × 3) and methylene chloride (1 L × 3) to give *n*-hexane (50.6 g), methylene chloride (9.7 g) fractions and the acidic aqueous phase. The acidic aqueous phase was then basified using 25% ammonia solution (NH_4_OH) (9 < pH < 10) and partitioned with methylene chloride (1 L × 3) to give 24.5 g of alkaloid-rich fraction. The *n*-hexane fraction (49.0 g) was subjected to column chromatography and eluted with the mixture of *n*-hexane/CH_2_Cl_2_ of increasing polarity [(9:1–7:3, *v*/*v*), CH_2_Cl_2_/MeOH (10:0–0:10, *v*/*v*)] to afford three main subfractions F1–F3. Subfraction F1 (20.3 g) underwent series of column chromatography eluted with *n*-hexane/EtOAc of increasing polarity (9:1–10:0, *v*/*v*) to yield compounds **18** (100.2 mg, *n*-hexane/EtOAc, 9.5:0.5, *v*/*v*), **21** (15.2 mg, *n*-hexane/EtOAc, 9:1, *v*/*v*), and the mixture of compounds **19**, and **20** (40.3 mg, *n*-hexane/EtOAc, 8.5:1.5, *v*/*v*). The purification of the subfraction F2 (14.7 g) with a silica gel column yielded compounds **26** (7.3 mg, *n*-hexane/EtOAc, 9:1, *v*/*v*), **27** (5.2 mg, *n*-hexane/EtOAc, 8:2, *v*/*v*), and **30** (103.0 mg, *n*-hexane/EtOAc, 7:3, *v*/*v*). F3 was a complex mixture and was not studied.

In addition, the CH_2_Cl_2_ fraction (9.7 g) was subjected to CC over silica gel and eluted with cyclohexane/EtOAc (8:2–2:8, *v*/*v*), EtOAc/MeOH (10:0–0:10) of increasing polarities to give 150 fractions (with a collection volume of 200 mL per fraction). These fractions were gathered based on their TLC profiles to give 3 main subfractions (G1–G3). The silica gel column chromatography of the subfraction G1 (2.5 g) gave a mixture of compounds **24** and **25** (50 mg, *n*-hexane/EtOAc, 7.5:2.5, *v*/*v*) and compound **22** (15.2 mg, *n*-hexane/EtOAc, 7:3, *v*/*v*). Furthermore, subfraction G2 (1.5 g) was subjected to CC over silica to afford compound **23** (10.1 mg, *n*-hexane/EtOAc, 6:4, *v*/*v*). The subfraction G3 (3.1 g) was subjected over silica gel and eluted with cyclohexane/EtOAc by the gradient of increasing polarity (8:2–5.5:4.5, *v*/*v*) to afford compound **32** (150.0 mg, cyclohexane/EtOAc, 7.5:3.5, *v*/*v*), and subfractions G31 (50.0 mg) and G32 (25.5 mg). Subfraction G31 (50.0 mg) eluted with cyclohexane/EtOAc of increasing polarities (7.5:2.5–3:7, *v*/*v*) led to isolation of compounds **31** (10.5 mg) and **26** (7.3 mg). The subfraction G32 (25.2 mg) was subjected to CC over Sephadex LH-20 to yield compound **29** (10.2 mg).

The CH_2_Cl_2_ alkaloid-rich fraction (22.0 g) was subjected to silica gel column chromatography and eluted with a gradient of increasing polarity with CH_2_Cl_2_/acetone/NH_4_OH (100:0:0.1–0:100:0.1, *v*/*v*/*v*) to give four subfractions (ALK1–ALK4). Subfraction ALK1 (2.1 g) was subjected to column chromatography and eluted with CH_2_Cl_2_/MeOH/NH_4_OH of increasing polarity (98:2:0.1–40:60:0.1, *v*/*v*/*v*) to afford compounds **14** (5.3 mg, 40:60, *v*/*v*) and **15** (4.8 mg, 98:2, *v*/*v*). ALK2 (4.2 g) was subjected to silica gel column and eluted with the mixture of acetone/MeOH/NH_4_OH (9:1:0.1–8.5:1.5:0.1, *v*/*v*/*v*) to give three sub-fractions (ALK21-ALK23). The sub-fraction ALK21 (0.4 g) was subjected to CC over Sephadex LH-20 gel and eluted with MeOH/H_2_O (85:15, *v*/*v*) to afford compounds **5** (12.3 mg) and **11** (4.8 mg). ALK3 (6.4 g) was purified through CC over silica gel to give two subfractions ALK31 and ALK32. A silica gel column chromatography of ALKA31 (0.95 g) afforded compounds **3** (5.0 mg, EtOAc/MeOH/NH_4_OH, 9:1:0.1, *v*/*v*/*v*) and **16** (3.4 mg, EtOAc/MeOH/NH_4_OH, 8.5:1.5:0.1, *v*/*v*/*v*). ALK4 was not studied because of its complexity.

### 2.5. UHPLC/MS/MS Data Processing

Mass spectrometry data were processed using Mzmine (version 4.3.0) and MSConvert (version 3.0.24267-b37d261) prior to uploading into the Structural Similarity Network Annotation Platform for Mass Spectrometry (SNAP-MS) database, available at www.npatlas.org/discover/snapms (accessed on 5 January 2025). The SNAP-MS parameters were set as follows: reference database, COCONUT; adduct, [M + H]^+^; mass error tolerance, 10 ppm; maximum cluster size, 5000; minimum compound group size, 3; minimum NPAtlas annotation cluster size, 3; and minimum result edges, 10,000. The analysis generated a Cytoscape-compatible file, which was visualized using Cytoscape (version 3.10.2) to obtain the molecular network. Filtering of the SNAP-MS network was performed using Sirius (version 5.8.6) and by comparison with the literature data.

### 2.6. SwissADME, SwissTarget Prediction and In Silico Oral Toxicity

In Silico prediction of pharmacokinetic and drug-likeness parameters remains very useful in drug discovery, as it accelerates development, reduces costs, minimizes risks, and helps prioritize promising candidates [16]. In this study, pharmacokinetic properties and drug-likeness were evaluated using SwissADME web tool (http://www.swissadme.ch/index.php, accessed on 20 March 2025). The target prediction of compounds that fulfilled drug-likeness criteria with favorable pharmacokinetic profiles was performed using the SwissTargetPrediction platform (http://www.swisstargetprediction.ch/ accessed on 20 March 2025). In silico oral toxicity was assessed using the ProTox-II tool (https://tox.charite.de/protox3/ accessed on 13 June 2025).

## 3. Results and Discussion

### 3.1. UHPLC-ESI-QTOF-MS/MS Identification of Compounds

**Figure 1:**
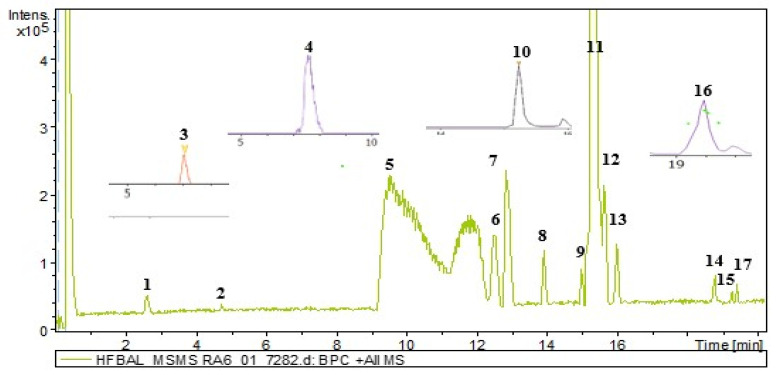
Base Peak Chromatogram (BPC) of the alkaloid fraction from the stem bark of *H. floribunda*.

**Figure 2:**
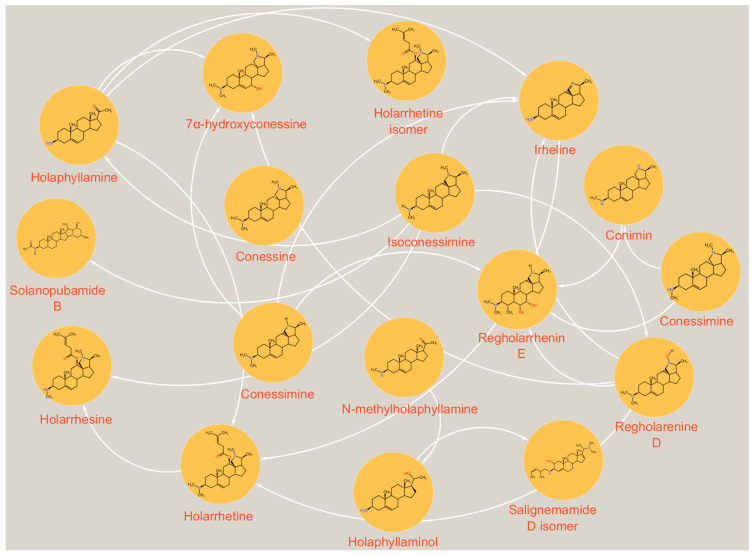
SNAP-MS networking of the alkaloid fraction of the stem bark of *H. floribunda*.

**Figure 3:**
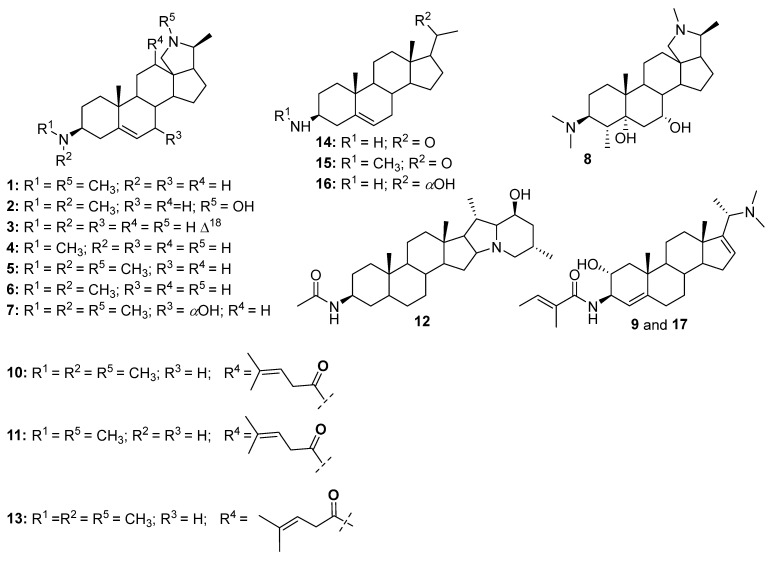
Structures of steroidal alkaloids identified from the stem bark of *H. floribunda* (**1**–**17**).

The alkaloid-rich fraction of the stem bark of *H. floribunda* was analyzed by UHPLC-ESI-Q-TOF-MS/MS (Figure 1). Peaks absent from the base peak chromatogram (BPC) of the alkaloid-rich fraction were extracted and subjected to MS/MS fragmentation (Figure 1). Seventeen steroidal alkaloids (**1**–**17**) were identified by interpreting their fragmentation pathways (see Supporting Information), in comparison with the literature data and with the aid of a freely accessible SNAP-MS platform (www.npatlas.org/discover/snapms, accessed on 28 August 2025), which generated the corresponding subnetworks (Figure 2). The identified steroidal alkaloids were classified into two subclasses including the conanine-type [isoconessimine (**1**), regholarrhenine D (**2**), irheline (**3**), conimin/conamine (**4**), conessine (**5**), conessimine (**6**), 7*α*-hydroxylconessine (**7**), regholarrhenine E (**8**), holarrhetine (**10**), holarrhesine (**11**), solanopubamide B (**12**), and holarrhetine isomer (**13**)] and the pregnene-type [salignemamide D (**9**), holaphyllamine (**14**), holaphyllaminol (**15**), *N-*methylholaphyllamine (**16**), and Salignemamide D isomer (**17**)] (Table 1, Figure 3).

### 3.2. Isolation of Compounds

**Figure 4:**
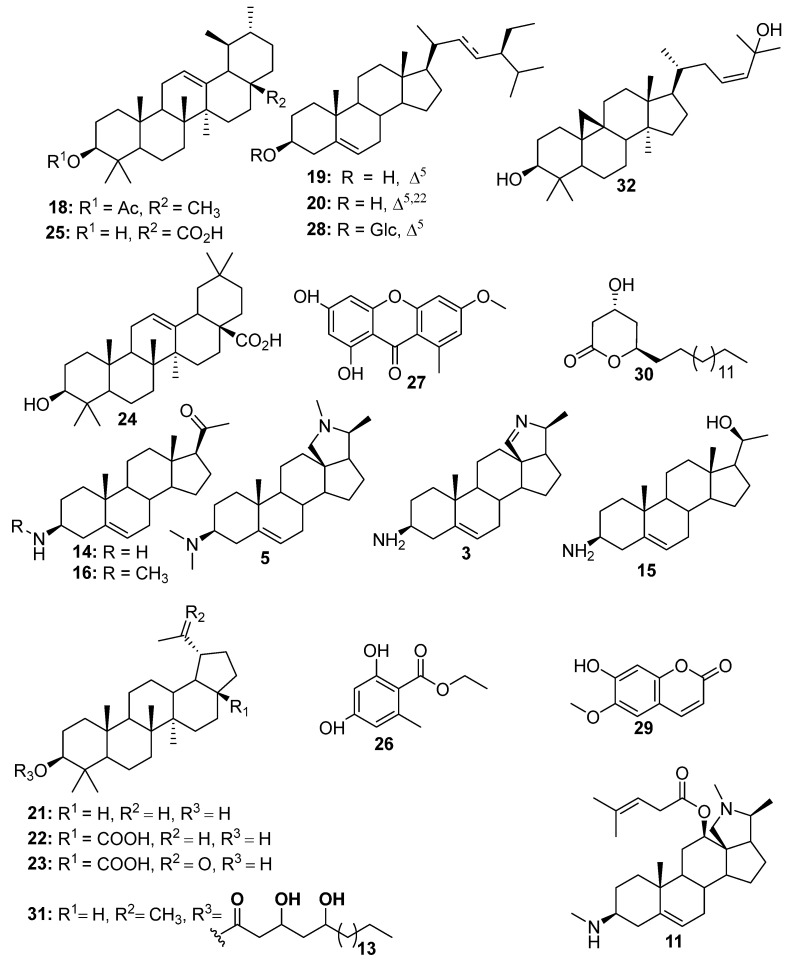
Structures of isolated compounds from the stem bark of *H. floribunda*.

In a view to further confirm the chemical composition of the active alkaloid-rich fraction, it underwent a series of column chromatography over silica gel and Sephadex LH-20 to afford six steroidal alkaloids, including irheline (**3**) [18], conessine (**5**) [17], holarhesine (**11**) [17], holaphyllamine (**14**) [10], holaphyllaminol (**15**) [18], and the mixture of *N*-methylholaphyllamine (**16**) [18] and irheline (**3**) [18] (Figure 4).

In addition, the *n-*hexane fraction displayed moderate in vitro antiplasmodial activity against the chloroquine-sensitive and multi-resistant strains of *Plasmodium falciparum* 3D7 (*Pf*3D7) and Dd2 (*Pf*Dd2), respectively. This fraction was purified by a series of column chromatography over silica gel and Sephadex LH-20 to afford lupeol (**21**) [26] and *α*-amyrin acetate (**18**) [27], a mixture of stigmasterol (**20**) and *β*-sitosterol (**19**) [28], ethylorsalinate (**26**) [29], the aliphatic lactone 3-*β*-hydroxyicosan-1,5-*β*-olide (**30**) [30], and lichexanthone (**27**) [31] (Figure 4).

The CH_2_Cl_2_ fraction was inactive against the chloroquine-sensitive (3D7) and multi-resistant (Dd2) strains of *P. falciparum*. Nevertheless, in a view to contribute to the chemotaxonomic knowledge on the plant, this fraction was purified investigated and led to the isolation of betulinic acid (**22**) [32], platanic acid (**23**) [33], oleanolic acid (**24**) [34], ursolic acid (**25**) [35], lupeol 3,5-dihydroxyeicosanoate (**31**) [36], cycloart-23*Z*-en-3*β*,25-diol (**32**) [37], *β*-sitosterol-3-*O*-*β*-D-glucopyranosyl (**28**) [28], and scopoletin (**29**) [38] (Figure 4).

### 3.3. Chemotaxonomic Significance

To the best of our knowledge, ethylorsalinate (**26**) and 3-*β*-hydroxyicosan-1,5-*β*-olide (**30**) were isolated for the first time from the Apocynaceae family. Lichexanthone (**27**), scopoletin (**29**), and platanic acid (**23**) are reported in this genus for the first time. However, they were already isolated from other plants of the Apocynaceae family. Oleanolic acid and ursolic acid were already reported in *H. curtisii* King and Gamble [27], while conessine (**5**), holarhesine (**11**), holaphyllamine (**14**), and *N*-methylholaphyllamine (**16**) were also reported in *H. africana* [10]. Irehline (**3**) and holaphyllaminol (**15**) have been reported in *H. pubescens* [18]. Among the putatively identified compounds, **9** and **12** are reported for the first time in the Apocynaceae family. Nevertheless, they have been reported in the Buxaceae and Solanaceae families [23,25]. The other herein identified steroidal alkaloids (**1**–**16**) were already reported in the genus *Holarrhena*. Thus, the present work stands as a significant contribution to the chemotaxonomic knowledge on *H. floribunda*.

### 3.4. Biological Contribution

As far as the in vitro antiplasmodial activity is concerned, the crude ethanol extract of the stem bark of *H. floribunda* was tested in vitro and showed good to moderate activity toward the *Pf*3D7 and *Pf*Dd2 strains with IC_50_ values of 14.12 ± 3.10 and 23.32 ± 2.39 µg/mL, respectively [39]. The *n*-hexane soluble fraction from the crude ethanol extract showed moderate activity against the *Pf*3D7 and *Pf*Dd2 with IC_50_ values of 36.40 ± 1.60 and 43.54 ± 2.74 µg/mL, respectively [39]. The methylene chloride soluble fraction from the crude extract was inactive (IC_50_ > 100 µg/mL) against both strains. The alkaloid-rich fraction showed good in vitro antiplasmodial activity, with IC_50_ values of 6.55 ± 0.60 and 8.27 ± 0.70 µg/mL against *Pf*3D7 and *Pf*Dd2, respectively (Table 2) [39]. These results could be justified by the antagonistic effect and/or the low concentration of some constituents in the ethanol crude extract of the stem bark of *H. floribunda.* The in vitro antiplasmodial activity of the alkaloid-rich fraction of this plant is reported herein for the first time, to the best of our knowledge. Even though all the fractions did not display an antiplasmodial activity against the assessed strain of *Plasmodium*, the antiplasmodial potentials of *H. floribunda* extracts were reported by Fotie and collaborators in 2006 against *Pf*D6 and *Pf*W2.

Conessine (**5**) showed moderate activity against the *Pf*3D7 and *Pf*Dd2 strains with IC_50_ values of 25.97 and 28.51 µM, respectively. In addition, amongst the assessed compounds, lichexanthone (**27**) showed moderate activity towards the aforementioned strains with IC_50_ values of 33.41 and 36.46 µM, respectively. Furthermore, holaphyllamine (**14**) showed marginal activity with an IC_50_ value of 49.53 and 55.78 µM on *Pf*3D7 and *Pf*Dd2, respectively. However, compound **14** exhibited good cytotoxicity against the human cervix carcinoma KB-3-1 cancer cell lines with an IC_50_ value of 9.8 µM as compared to cryptophycin (IC_50_ = 1.3 × 10^−5^ μM), according to the National Cancer Institute [40].

The in vitro antiplasmodial activity of some identified and isolated steroids (**1**, **3**–**5**, **10**, **11**, **15**, and **16**) against *Plasmodium falciparum* FCB1 and K1 strains had been reported in previous studies. Indeed, these compounds showed good in vitro antiplasmodial activity, with IC_50_ values ranging from 0.97 to 11.7 µM against the chloroquine-resistant FCB1 and multi-resistant K1 strains of *P. falciparum*, respectively (Table 3) [17,18,41].

### 3.5. In Silico Pharmacokinetic Parameters, Drug-likeness Properties and Targets Prediction of Some Identified Compounds

Given the promising in vitro antiplasmodial activity of the alkaloid-rich fraction from the stem bark extract of *H. floribunda*, selected alkaloids identified or isolated (**1**, **3, 4**, **5**, **10**, **11**, **14**, **15**, and **16**) were subjected to computational analysis using the SwissADME predictor to evaluate their physicochemical, pharmacokinetic, and drug-likeness properties (Table 4). Target prediction was also performed for compounds fulfilling drug-likeness criteria using the SwissTarget predictor. The analysis revealed that all selected compounds displayed high gastrointestinal absorption and a bioavailability score of 0.55, in agreement with previously reported bioavailability data [42]. Most of the compounds were moderately or poorly soluble in water. Amongst the tested compounds, only compound **14** complied fully with Lipinski’s rule of five, while all compounds satisfied Veber’s and Egan’s rules. Overall, compound **14** emerged as the most promising candidate, showing no Lipinski violations and favorable predicted pharmacokinetic parameters. Among the compounds evaluated in the in silico study, several, including conessine (**5**), holarrhesine (**10**), hollaphyllamine (**14**), and *N*-methylholaphyllamine (**16**), were predicted to exhibit good brain penetration. Moreover, all the tested compounds showed high gastrointestinal absorption.

**Figure 5:**
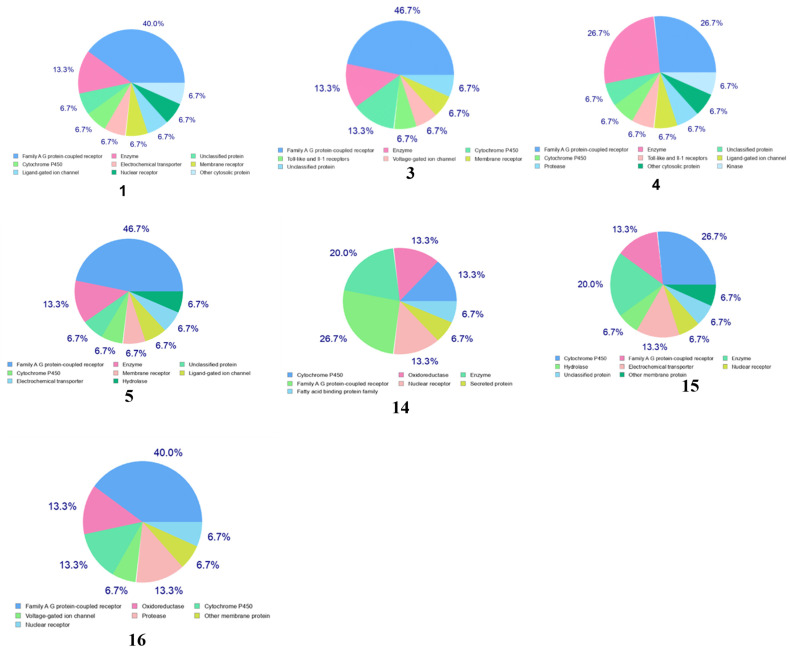
Pie chart of target prediction of compounds (**5**, **14**, and **16**).

To assess the potential interaction of the compounds with protein targets, those fulfilling drug-likeness criteria (compounds **1**, **3**, **4**, **5**, **14**, and **16**) were subjected to target prediction using the SwissTarget Predictor. The prediction results, illustrated in the pie chart (Figure 5), indicated that compound **1** was most likely to inhibit enzymes (13.3%), family A G protein-coupled receptors (40%), and cytochrome P450 enzymes (6.7%). Similarly, compound **3** was predicted to inhibit enzymes (13.3%), family A G protein-coupled receptors (46.7%), and cytochrome P450 enzymes (13.3%). Compound **4** was predicted to inhibit enzymes (26.7%), family A G protein-coupled receptors (26.7%), and cytochrome P450 enzymes (6.7%). Also, compound **15** was predicted to inhibit cytochrome P450 enzymes (26.7%), hydrolases (6.7%), family A G protein-coupled receptors (13.3%), and enzymes (20%). Compound **14** was predicted to inhibit cytochrome P450 enzymes (13.3%), family A G protein-coupled receptors (26.7%), enzymes (20%), oxidoreductases (13.3%), and nuclear receptors (13.3%). Compound **16** was predicted to inhibit cytochrome P450 enzymes (13.3%), family A G protein-coupled receptors (40%), proteases (13.3%), and oxidoreductases (13.3%) (Figure 5). Most of the selected compounds are not substrates for P-pg and CYP (Cytochromes P-450 isoenzymes).

### 3.6. In Silico Oral Toxicity Prediction

The toxicity profile of a compound is a critical parameter in drug discovery [43]. Compounds **5**, **14**, and **27**, which showed the highest activity against *P. falciparum*, were further subjected to an in silico toxicity evaluation to assess their safety. Compound **27** was predicted to be the least toxic, with an LD_50_ of 3200 mg/kg and classified as toxicity class V [44]. Compounds **5** and **27** exhibited LD_50_ values of 750 and 390 mg/kg, respectively, and were classified as toxicity class IV [44]. In addition, compounds **5** and **14** were predicted to be devoid of hepatotoxicity, nephrotoxicity, and cardiotoxicity (Table 5), although they showed potential respiratory toxicity with prediction scores ranging from 0.73 to 0.77. Similarly, compound **27** was predicted to lack hepatotoxicity but to display potential nephrotoxicity, cardiotoxicity, and respiratory toxicity (Table 5).

## 4. Conclusions

In summary, this study adopted a bioguided approach to identify antiplasmodial pharmacophores, which could serve as starting points for the development of novel antimalarial agents. The results highlight the promising activity of certain fractions of *H. floribunda* against both chloroquine-sensitive and multidrug-resistant strains of *P. falciparum*. Chemical analysis of this plant enabled detailed characterization of its alkaloid-rich fraction, revealing a diverse group of steroidal alkaloids with favorable drug-like properties as predicted by in silico assessments. The isolation of key active compounds further underscores the therapeutic potential inherent in the stem bark. Importantly, the observed safety margins suggest that these compounds have promise for further development. Overall, this work advances our understanding of the chemical constituents responsible for the plant’s traditional use in malaria treatment and supports its potential as a source for future antimalarial drug development.

## Figures and Tables

**Table 1:** Compounds identified from the stem bark of *H. floribunda*.

N°	RT (min)	[M + H]^+^ Exp	[M + H]^+^ Theo	MS/MS Patterns	Names	References
**1**	2.6	343.3113	343.3108	312, 269	Isoconessimine (**1**)	[17]
**2**	4.7	359.3059	359.3057	341, 191	Regholarrhenine D (**2**)	[18]
**3**	7.0	313.2684	313.2638	/	Irheline (**3**)	[18]
**4**	7.5	329.2949	329.2951	269	Conimin/conamine (**4**)	[19]
**5**	9.3	357.3275	357.3264	312, 269	Conessine (**5**)	[10]
**6**	12.5	343.3163	343.3108	298, 269	Conessimine (**6**)	[17]
**7**	12.8	373.3259	373.3214	312, 269	7*α*-hydroxyconessine (**7**)	[20]
**8**	14.0	405.3402	405.3476	312, 267	Regholarrhenine E (**8**)	[21]
**9**	15.0	441.3476	441.3476	327, 267, 157	Salignemamide D (**9**)	[22]
**10**	15.2	469.3793	469.3789	455, 424, 343	Holarrhetine (**10**)	[17]
**11**	15.3	455.3653	455.3632	267, 341	Holarrhesine (**11**)	[17]
**12**	15.5	457.3781	457.3789	441, 427	Solanopubamide B (**12**)	[23]
**13**	16.0	469.3778	469.3789	455, 441, 424	Holarrhetine isomer (**13**)	/
**14**	18.7	316.2674	316.2635	161	Holaphyllamine (**14**)	[24]
**15**	19.2	318.2796	318.2791	161, 261	Holaphyllaminol (**15**)	[24]
**16**	19.3	330.2789	330.2791	261, 161, 316	*N-*methylholaphyllamine (**16**)	[10]
**17**	19.4	441.3476	441.3476	327, 267	Salignemamide D isomer (**17**)	[25]

Salignemamide D (**9**) and Solanopubamide B (**12**) are reported here for the first time from the genus *Holarrhena*. However, they were previously isolated from *Solanum pubescens* (Solanaceae) and *Sarcococca saligna* (Buxaceae), respectively [21,25].

**Table 2:** In Vitro antiplasmodial and cytotoxicity results of extract, fractions, and compounds from the stem bark of *H. floribunda*.

Extract/Fractions/ Compounds	IC_50_/*Pf*3D7	IC_50_)/*Pf*Dd2	RI: [IC_50_*Pf*Dd2/IC_50_*Pf*3D7]	CC_50_ on Vero Cells	CC_50_ on KB-3-1
	IC_50_ Values [Compound and Reference Drug (µM) and Extracts and Fractions (µg/mL)]				
EtOH crude extract	14.11 ± 3.06	23.32 ± 2.39	1.65	>500 µg/mL	ND
*n*-hexane fraction	36.36 ± 1.59	43.54 ± 2.74 µg/mL	1.19	>500 µg/mL	ND
CH_2_Cl_2_ fraction	>100	>100	-	>500 µg/mL	ND
*n*-butanol fraction	NT	NT	-	ND	ND
Alkaloid fraction	6.54 ± 0.570	8.27 ± 0.650	1.26	162.9 ± 3.970 µg/mL	NT
conessine (**5**)	25.97 ± 0.43	28.51 ± 0.704	1.09	NT	NT
Irheline (**3**)	>50	>50	-	NT	NT
Holarrhesine (**11**)	NT	NT	-	NT	NT
Holaphyllamine (**14**)	49.53 ± 4.830	55.78 ± 9.750 µM	1.12	NT	9.8 µM
Holaphylaminol (**15**)	NT	NT	-	NT	NT
Betulinic acid (**22**)	NT	NT	-	NT	NT
Ethylorsalinate (**26**)	>50	>50	-	NT	NT
Lichexanthone (**27**)	33.41 ± 1.200	36.46 ± 1.39	1.09	NT	NT
Chloroquine *	0.045 ± 0.003	0.73 ± 0.090	16.22	NT	NT
Artemisinin *	0.035 ± 0.000	0.025 ± 0.006	0.71	NT	NT
Cryptophycin *	-	-	-	-	1.3 × 10^−5^ μM

IC_50_ = half inhibitory concentration, CC_50_ = cytotoxic concentration 50%, * = Reference drug, NT = Not Tested, RI = Resistance Index.

**Table 3:** In vitro antiplasmodial activity of some identified and isolated steroidal alkaloids reported in the literature.

Names	IC_50_ (µM)	References
Isoconessimine (**1**)	3.39 ± 079 (*Pf* FCB1)	[17]
Irheline (**3**)	1.2 (*Pf* K1)	[18]
Conimin (**4**)	8.0 (*Pf* K1)	
Conessine (**5**)	1.04 ± 014 (*Pf* FCB1)	[17]
Holarrhetine (**10**)	1.13 ± 0.32 (*Pf* FCB1)	
Holarrhesine (**11**)	0.97 ± 0.11 (*Pf* FCB1)	
*N-*methylholaphyllamine (**16**)	10.6 (*Pf* K1)	[18]
Holaphyllaminol (**15**)	11.7 (*Pf* K1)	
Chloroquine *	0.13 ± 0.03	[17]

IC_50_ = half inhibitory concentration, *Pf: Plasmodium falciparum*, * = Reference drug.

**Table 4:** Drug-likeness, physicochemical parameters and lead-likeness of some alkaloids identified from the root and the stem bark of *H. floribunda*.

	Holarrhesine (11)	Irheline (3)	Holarrhetine (10)	Holaphyllaminol (15)	Holaphyllamine (14)	Conessine (5)	*N*-methylholaphyllamine (16)	Isoconessimine (1)	Conimin (4)
**Physicochemical Property**									
MW (g/mol)	454.69	312.49	468.71	317.51	315.49	356.59	329.52	342.56	328.53
**Pharmacokinetics**									
GI absorption	High	High	High	High	High	High	High	High	High
BBB permeant	Yes	Yes	Yes	Yes	No	Yes	Yes	Yes	Yes
P-gp substrate	No	No	No	Yes	No	No	No	No	No
CYP1A2 inhibitor	No	No	No	No	No	No	No	No	No
CYP2C19 inhibitor	No	No	No	No	No	No	No	No	No
CYP2C9 inhibitor	No	No	No	No	No	No	No	No	No
CYP2D6 inhibitor	No	No	No	No	No	No	No	No	No
CYP3A4 inhibitor	No	No	No	No	No	No	No	No	No
**Lipophilicity**									
Log *K*_p_ (skin permeation)	−5.24 cm/s	−6.15 cm/s	−4.99 cm/s	−5.20 cm/s	−5.41 cm/s	−5.03 cm/s	−5.14 cm/s	−5.27 cm/s	−5.52 cm/s
Log *S* (ESOL)	−5.73	−3.60	−6.11	−4.44	−4.22	−5.04	−4.57	−4.66	−4.28
Solubility	8.45 × 10^−4^ mg/mL; 1.86 × 10^−6^ mol/L	7.88 × 10^−2^ mg/mL; 2.52 × 10^−4^ mol/L	3.60 × 10^−4^ mg/mL; 7.69 × 10^−7^ mol/L	1.16 × 10^−2^ mg/mL; 3.64 × 10^−5^ mol/L	1.88 × 10^−2^ mg/mL; 5.96 × 10^−5^ mol/L	3.25 × 10^−3^ mg/mL; 9.11 × 10^−6^ mol/L	8.93 × 10^−3^ mg/mL; 2.71 × 10^−5^ mol/L	7.43 × 10^−3^ mg/mL; 2.17 × 10^−5^ mol/L	1.72 × 10^−2^ mg/mL; 5.24 × 10^−5^ mol/L
Class	Moderately soluble	Soluble	Poorly soluble	Moderately soluble	Moderately soluble	Moderately soluble	Moderately soluble	Moderately soluble	Moderately soluble
Log *S* (Ali)	−6.03	−3.36	−6.33	−4.96	−3.90	−4.72	−4.80	−4.43	−4.12
Solubility	4.27 × 10^−4^ mg/mL; 9.38 × 10^−7^ mol/L	1.38 × 10^−1^ mg/mL; 4.40 × 10^−4^ mol/L	2.19 × 10^−4^ mg/mL; 4.67 × 10^−7^ mol/L	3.45 × 10^−3^ mg/mL; 1.09 × 10^−5^ mol/L	3.94 × 10^−2^ mg/mL; 1.25 × 10^−4^ mol/L	6.79 × 10^−3^ mg/mL; 1.90 × 10^−5^ mol/L	5.21 × 10^−3^ mg/mL; 1.58 × 10^−5^ mol/L	1.38 × 10^−2^ mg/mL; 4.04 × 10^−5^ mol/L	2.47 × 10^−2^ mg/mL; 7.51 × 10^−5^ mol/L
**Drug-likeness**									
Lipinski	Yes; 1 violation: MLOGP > 4.15	Yes; 0 violation	Yes; 1 violation: MLOGP > 4.15	Yes; 1 violation: MLOGP > 4.15	Yes, 0 violation	Yes; 1 violation: MLOGP > 4.15	Yes; 1 violation: MLOGP > 4.15	Yes; 1 violation: MLOGP > 4.15	Yes; 1 violation: MLOGP > 4.15
Ghose	No; 2 violations: MR > 130, atoms > 70	Yes	No; 2 violations: MR > 130, atoms > 70	Yes	Yes	Yes	Yes	Yes	Yes
Veber	Yes	Yes	Yes	Yes	Yes	Yes	Yes	Yes	Yes
Egan	Yes	Yes	Yes	Yes	Yes	Yes	Yes	Yes	Yes
Muegge	No; 1 violation: XLOGP3 > 5	Yes	No; 1 violation: XLOGP3 > 5	Yes	Yes	Yes	Yes	Yes	Yes
**Medicinal Chemistry**									
Bioavailability Score	0.55	0.55	0.55	0.55	0.55	0.55	0.55	0.55	0.55
Lead-likeness	No; 2 violations: MW > 350, XLOGP3 > 3.5	Yes	No; 2 violations: MW > 350, XLOGP3 > 3.5	No; 1 violation: XLOGP3 > 3.5	No; 1 violation: XLOGP3 > 3.5	No; 2 violations: MW > 350, XLOGP3 > 3.5	No; 1 violation: XLOGP3 > 3.5	No; 1 violation: XLOGP3 > 3.5	No; 1 violation: XLOGP3 > 3.5

MW: molecular weight, Log s: aqueous solubility (scale: <−10 < Poorly < −6 < Moderately <−4 < soluble < −2 very < 0<Highly); BBB: Blood–brain barrier; Lipinsky (Criteria: MW ≤ 500; MLOGP ≤ 4.15; N or O ≤ 10; NH or OH ≤5); Ghose (Criteria: 160 ≤ MW ≤ 480; −0.4 ≤ WLOGP ≤ 5.6; 40 ≤ MR ≤ 130; 20 ≤ atoms ≤ 70); Veber (criteria: rotatable bonds ≤ 10, TPSA ≤ 140); Egan (criteria: WLOGP ≤ 5.88, TPSA ≤ 131.6); Muegge, Num. ring ≤ 7, Num. carbon > 4, Num. heteroatom > 1, num. rotatable bonds ≤ 15, H-bond acc ≤ 10, H-bond don. ≤ 5200 ≤ MW ≤ 600, −2 ≤ XLOGP ≤ 5); Lead-likeness: 250 ≤ MW ≤ 350, XLOGP ≤ 3.5, Num. rotatable bonds ≤ 7), GI absorption: gastrointestinal absorption.

**Table 5:** In Silico acute toxicity of bioactive compounds.

Targets Compounds	Hepatotoxicity (SP)	Nephrotoxicity (SP)	Cardiotoxicity (SP)	Respiratory Toxicity (SP)	LD_50_ (mg/kg)
Conesine (**5**)	Inactive (0.89)	Inactive (0.94)	Inactive (0.81)	Active (0.77)	750
Holaphyllamine (**14**)	Inactive (0.75)	Inactive (0.92)	Inactive (0.78)	Active (0.73)	390
Lichexanthone (**27**)	Inactive (0.75)	active (0.61)	Active (0.53)	Active (0.75)	3200

SP: Score of the prediction; Average for active compounds: Hepatotoxicity: 0.82; Nephrotoxicity: 0.75; Cardiotoxicity: 0.86; Respiratory toxicity: 0.78; Class I: fatal if swallowed (LD50 ≤ 5 mg/kg); Class II: fatal if swallowed (5 mg/kg < LD50 ≤ 50 mg/kg); Class III: toxic if swallowed (50 mg/kg < LD50 ≤ 300 mg/kg); Class IV: harmful if swallowed (300 mg/kg < LD50 ≤ 2000 mg/kg); Class V: may be harmful if swallowed (2000 mg/kg < LD50 ≤ 5000 mg/kg); Class VI: non-toxic (LD50 > 5000 mg/kg).

## Data Availability

Data are contained within the article and Supplementary Materials.

## Supplementary Materials

The following supporting information can be downloaded at: https://www.mdpi.com/article/10.3390/biom15101415/s1, Figures S1–S37: ^1^H and ^13^C NMR spectra of compounds **3**, **5**, **11**, **14**–**16** and **18**–**32**; Figure S38: Base Peak Chromatogram (BPC) of the alkaloid fraction from the stem bark of *H. floribunda*; Figures S39–S55: MS/MS spectra of compounds **1**, **2**, **4**–**11** and **14**–**16;** Scheme S1–S14: MS/MS fragmentation of compounds **1**, **2**, **4**–**14**, and **16**.

## Abbreviations

The following abbreviations are used in this manuscript:

TLC	Thin Layer Chromatography
UHPLC	Ultra-High Performance Liquid Chromatography
QTOF-ESI-MS/MS	Quadrupole Time Of Flight Electrospray Ionization Tandem Mass Spectrometry
SNAP-MS	Structural Similarity Network Annotation Platform for Mass Spectrometry
ADMET	Absorption, Desorption, Metabolism, Excretion and Toxicity
