# Supplementary material for: UHPLC-QTOF-ESI-MS/MS, SNAP-MS Identification, In Silico Prediction of Pharmacokinetic Properties of Constituents from the Stem Bark of Holarrhena floribunda (G. Don) T. Durand and Schinz (Apocynaceae)
Source: Biomolecules. 2025 Oct 4;15(10):1415. doi: 10.3390/biom15101415 (PMC12564223; doi:10.3390/biom15101415)
Supplement: Supplementary file 1 [file biomolecules-15-01415-s001.zip › biomolecules-3873852-supplementary.pdf]

Supporting material

Article

UHPLC-QTOF-ESI-MS/MS SNAP-MS identification, *in silico* prediction of pharmacokinetic properties of constituents from the stem bark of *Holarrhena floribunda* (G. Don) T. Durand and Schinz (Apocynaceae)

Franck Landry Djila Possi[1,2], MC Jesus Kinyok[2], Joseph Eric Mbasso Tameko[2], Bel Youssouf G. Mountessou[2], Johanne Kevine Jumeta Dongmo[1,2], Mariscal Brice Tchatat Tali[3], Appolinaire Kene Dongmo[4,5,*], Fabrice Fekam Boyom[3], Jean Jules Kezetas Bankeu[2], Norbert Sewald[4], Jean Rodolphe Chouna[5,*], Bruno Ndjakou Lenta[2,4,*]

[1] Department of Organic Chemistry, Faculty of Science, University of Yaoundé 1, P.O. Box 812, Yaoundé, Cameroon, possidjila@yahoo.com; jumetakevine@yahoo.fr

[2] Department of Chemistry, Higher Teacher Training College, University of Yaoundé 1, P.O. Box 47, Yaoundé, Cameroon; possidjila@yahoo.com; jesus-kinyok.mc@univ-yaounde1.cm; tamekombasso@yahoo.fr; mountessou@yahoo.com; jumetakevine@yahoo.fr; bk_jeanjules@yahoo.fr; lentabruno@yahoo.fr (B. N. L)

[3] Department of Biochemistry, Faculty of Science, University of Yaoundé 1, Yaoundé 812, Cameroon; b.tchatat@yahoo.com; fabrice.boyom@fulbrightmail.org

[4] Organic and Bioorganic Chemistry, Faculty of Chemistry, Bielefeld University, P.O. Box 100131, D-33501 Bielefeld, Germany; appolinaire.dongmo@uni-bielefeld.de; norbert.sewald@uni-bielefeld.de; bruno.lenta_ndjakou@uni-bielefeld.de

[5] Department of Chemistry, Faculty of Science, University of Dschang, P.O. Box 67, Dschang, Cameroon; appolinaire.dongmo@yahoo.fr; jean.chouna@univ-dschang.org

***** Correspondence: appolinaire.dongmo@uni-bielefeld.de or appolinaire.dongmo@yahoo.fr (A. K. D.); Tel.: +49 152 1295 0875

List of figures

Figure S1: ^{1}H NMR spectrum of compound **3** (CD_2Cl_2, 600 MHz) 5
Figure S2: ^{13}C NMR spectrum of compound **3** (CD_2Cl_2, 150 MHz) 5
Figure S3: ^{1}H NMR spectrum of compound **5** ($CDCl_3$, 600 MHz) 6
Figure S4: ^{13}C NMR spectrum of compound **5** ($CDCl_3$, 150 MHz) 6
Figure S5: ^{1}H NMR spectrum of compound **11** (CD_2Cl_2, 600 MHz) 7
Figure S6: ^{13}C NMR spectrum of compound **11** (CD_2Cl_2, 150 MHz) 7
Figure S7: ^{1}H NMR spectrum of compound **14** ($CDCl_3$/CD_3OD, 600 MHz) 8
Figure S8: ^{13}C NMR spectrum of compound **14** ($CDCl_3$/CD_3OD, 150 MHz) 8
Figure S9: ^{1}H NMR spectrum of compounds **3** and **15** ($CDCl_3$, 600 MHz) 9
Figure S10: ^{13}C NMR spectrum compounds **3** and **15** ($CDCl_3$, 150 MHz) 9
Figure S11: ^{1}H NMR spectrum of compound **16** ($CDCl_3$/CD_3OD, 600 MHz) 10
Figure S12: ^{13}C NMR spectrum of compound **16** ($CDCl_3$/CD_3OD, 150 MHz) 10
Figure S13: ^{1}H NMR spectrum of compound **18** ($CDCl_3$, 600 MHz) 11
Figure S14: ^{13}C NMR spectrum of compound **18** ($CDCl_3$, 150 MHz) 11
Figure S15: ^{1}H NMR spectrum of compounds **19** and **20** ($CDCl_3$, 600 MHz) 12
Figure S16 : ^{13}C NMR spectrum of compounds **19** and **20** ($CDCl_3$, 150 MHz) 12
Figure S17: ^{1}H NMR spectrum of compound **21** ($CDCl_3$, 600 MHz) 13
Figure S18: ^{13}C NMR spectrum of compound **21** ($CDCl_3$, 600 MHz) 13
Figure S19: ^{1}H NMR spectrum of compound **22** (Pyridine-d_5, 600 MHz) 14
Figure S20: ^{1}H NMR spectrum of compound **22** (Pyridine-d_5, 600 MHz) 14
Figure S21: ^{1}H NMR spectrum of compound **23** (Pyridine-d_5, 600 MHz) 15
Figure S22: ^{13}C NMR spectrum of compound **23** (Pyridine-d_5, 150 MHz) 15
Figure S23: ^{1}H NMR spectrum of compounds **24** and **25** (Pyridine-d_5, 600 MHz) 16
Figure S24: ^{1}H NMR spectrum of compound **26** ($CDCl_3$, 600 MHz) 16
Figure S25: ^{13}C NMR spectrum of compound **26** ($CDCl_3$, 150 MHz) 17
Figure S26: ^{1}H NMR spectrum of compound **27** ($CDCl_3$, 600 MHz) 17
Figure S27: ^{13}C NMR spectrum of compound **27** ($CDCl_3$, 150 MHz) 18
Figure S28: ^{1}H NMR spectrum of compound **28** (DMSO-d_6, 600 MHz) 18
Figure S29: ^{13}C NMR spectrum of compound **28** (DMSO-d_6, 600 MHz) 19
Figure S30: ^{1}H NMR spectrum of compound **29** (CD_2Cl_2, 600 MHz) 19
Figure S31: ^{13}C NMR spectrum of compound **29** (CD_2Cl_2, 150 MHz) 20
Figure S32: ^{1}H NMR spectrum of compound **30** (CD_2Cl_2, 600 MHz) 20
Figure S33: ^{13}C NMR spectrum of compound **30** (CD_2Cl_2, 150 MHz) 21
Figure S34 : ^{1}H NMR spectrum of compound **31** (CD_2Cl_2, 600 MHz) 21
Figure S35: ^{13}C NMR spectrum of compound **31** (CD_2Cl_2, 150 MHz) 22
Figure S36: ^{1}H NMR spectrum of compound **32** (CD_2Cl_2, 600 MHz) 22
Figure S37: ^{13}C NMR spectrum of compound **32** (CD_2Cl_2, 150 MHz) 23
Figure S38: Base Peak Chromatogram (BPC) of the alkaloid fraction from the stem bark of *H. floribunda* 23
Figure S39: MS/MS fragmentation pattern of isoconessimine (**1**) 24
Figure S40: MS/MS spectrum of regholarrhenin D (**2**) 24
Figure S41: MS/MS fragmentation pattern of irheline (**3**) 25
Figure S42: MS/MS spectrum of conimin (**4**) 25
Figure S43 : MS/MS spectrum of conessine (**5**) 27
Figure S44 : MS/MS spectrum of conessimine (**6**) 28

Figure S45: MS/MS spectrum of 7α-hydroxyconessine (**7**) 28
Figure S46: MS/MS spectrum of regholarrhenine E **(8)**.. 29
Figure S47: MS/MS spectrum of salignemamide D (**9**)... 30
Figure S48 : MS/MS spectrum of holarrhetine (**10**) 31
Figure S49: MS/MS spectrum of holarrhesine (**11**) 32
Figure S50: MS/MS spectrum of solanopubamide B (**12**)... 33
Figure S51: MS/MS spectrum of holarrhetine isomer (**13**) 34
Figure S52 : MS/MS spectrum of holaphyllamine (**14**).. 36
Figure S53: MS/MS spectrum of *N*-methylholaphyllamine (**16**)... 37
Figure S54: MS/MS fragmentation pattern of holaphyllaminol (**15**)...................................... 38
Figure S55: MS/MS fragmentation pattern of salignemamide D isomer (**17**)........................ 38
Figure S56: Structures of isolated compounds from the stem bark of *H. floribunda* 39

List of schemes

Scheme S1: MS/MS fragmentation of isoconessimine (**1**) 24
Scheme S2: MS/MS fragmentation of regholarrhenine D (**2**) 25
Scheme S3: MS/MS fragmentation of conimin (**4**) 26
Scheme S4: MS/MS fragmentation of conessine (**5**).................... 27
Scheme S5: MS/MS fragmentation of conessimine (**6**) 28
Scheme S6: MS/MS fragmentation of 7*α*-hydroxyconessine (7) 29
Scheme S7: MS/MS fragmentation of regholarrhenine E (**8**) 30
Scheme S8: MS/MS fragmentation of salignemamide D (**9**) 31
Scheme S9 : MS/MS fragmentation of holarrhetine (**10**) 32
Scheme S10 : MS/MS fragmentation of holarrhesine (**11**).................... 33
Scheme S11: MS/MS fragmentation of solanopubamide B (**12**) 34
Scheme S12: MS/MS fragmentation of holarrhetine isomer (**13**).................... 35
Scheme S13: MS/MS fragmentation of holaphyllamine (**14**) 36
Scheme S14: MS/MS fragmentation of *N*-methylholaphyllamine (**16**) 37

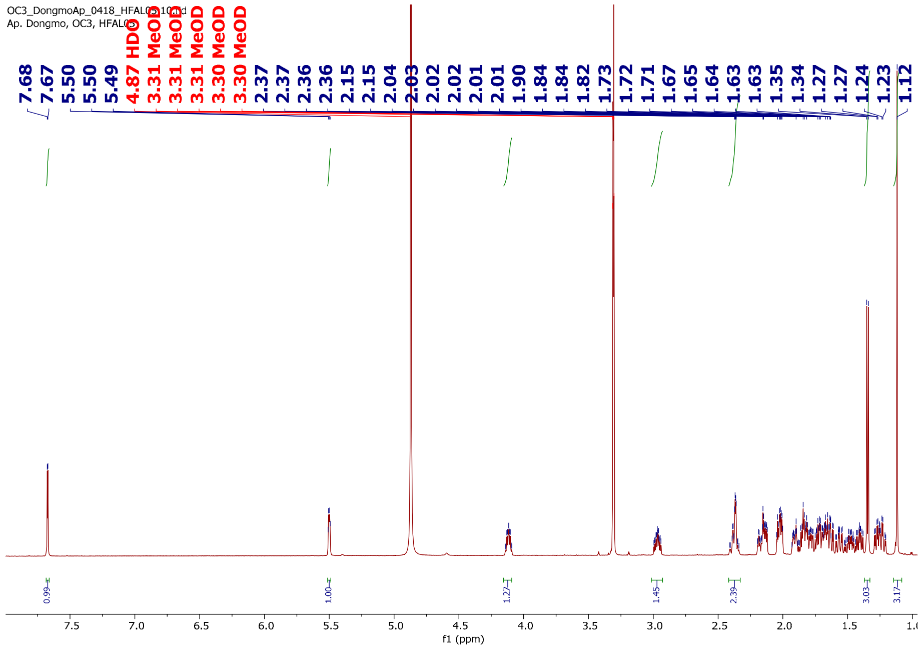

Figure S1: ^{1}H NMR spectrum of compound **3** (CD_2Cl_2, 600 MHz)

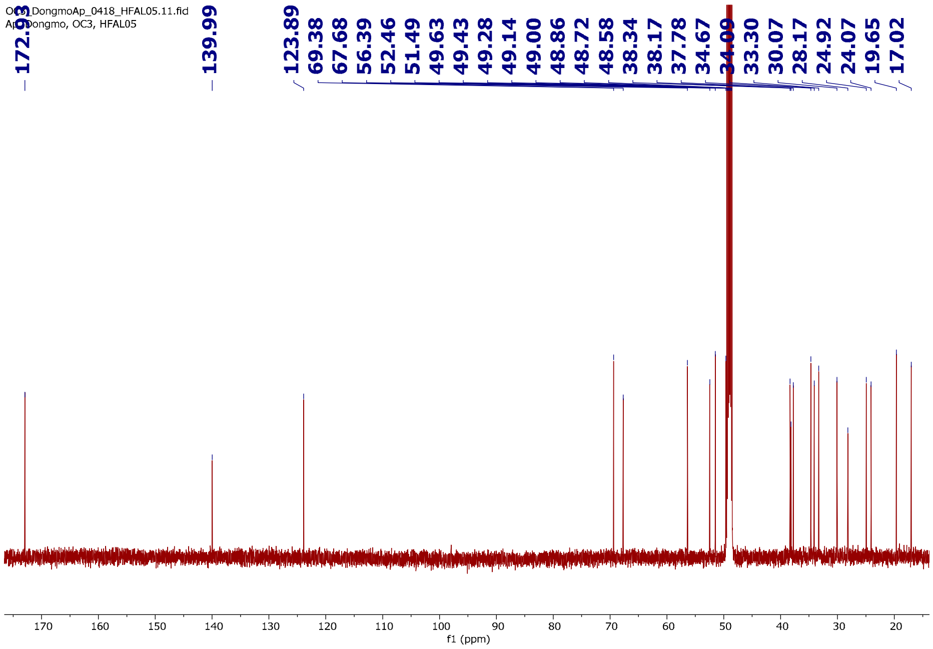

Figure S2: ^{13}C NMR spectrum of compound **3** (CD_2Cl_2, 150 MHz)

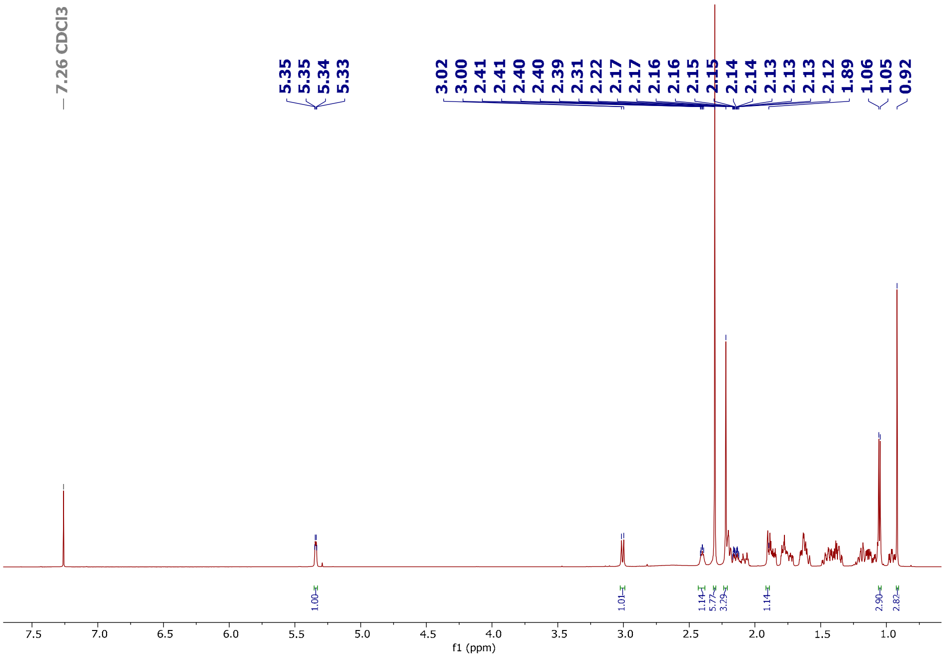

Figure S3: ^{1}H NMR spectrum of compound **5** ($CDCl_3$, 600 MHz)

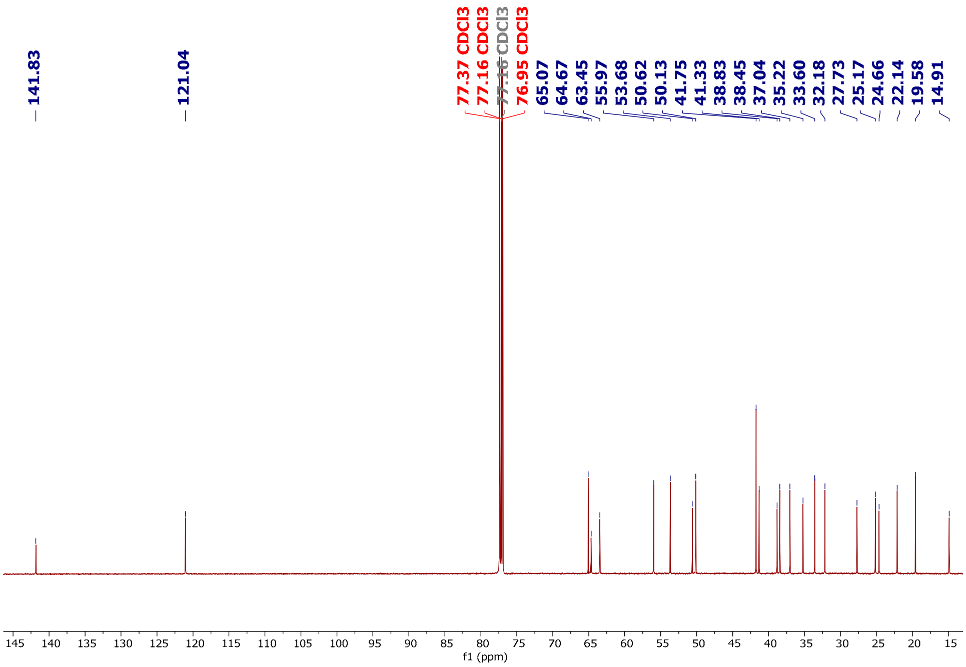

Figure S4: ^{13}C NMR spectrum of compound **5** ($CDCl_3$, 150 MHz)

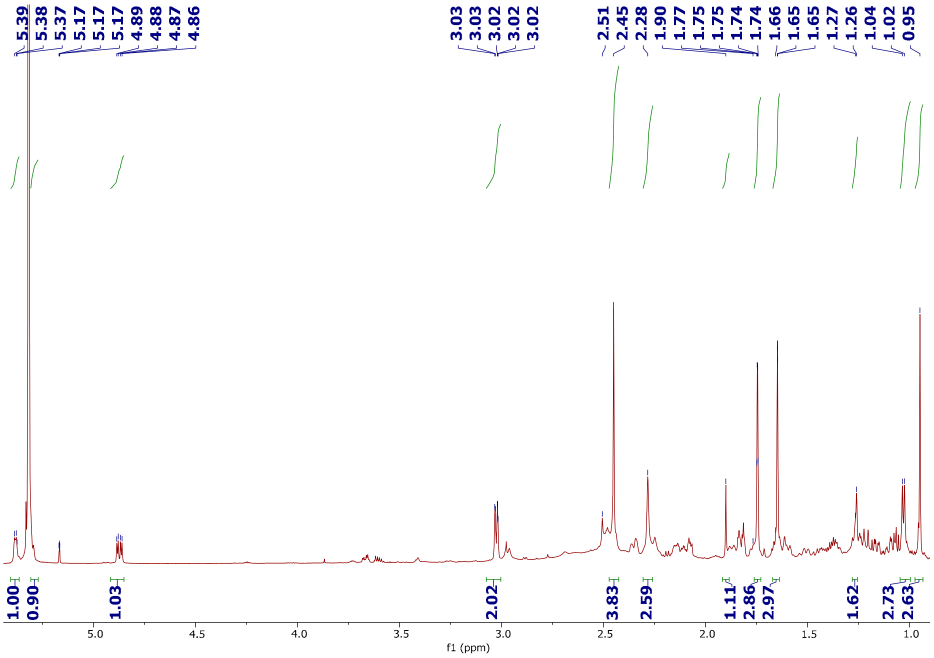

Figure S5: ^{1}H NMR spectrum of compound **11** (CD_2Cl_2, 600 MHz)

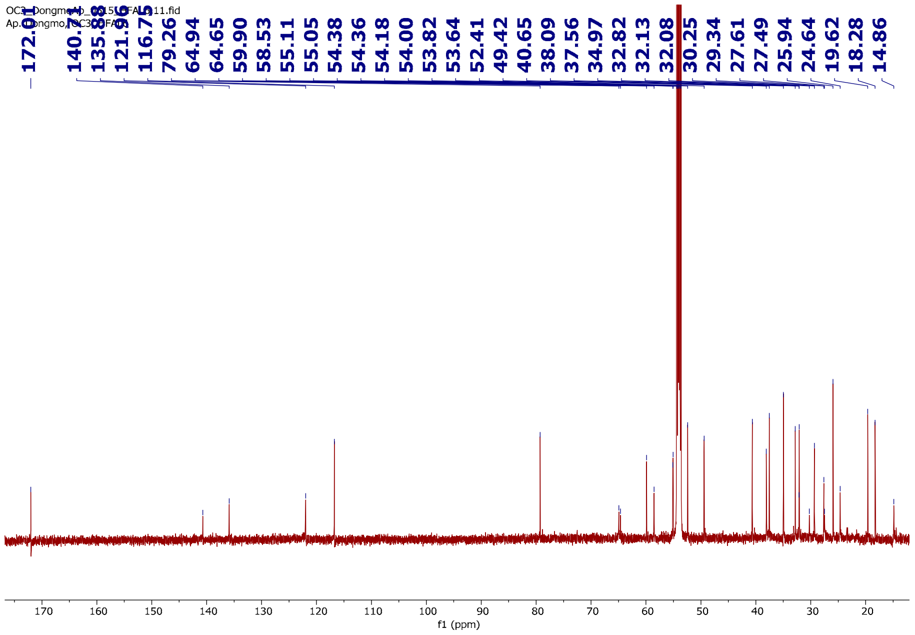

Figure S6: ^{13}C NMR spectrum of compound **11** (CD_2Cl_2, 150 MHz)

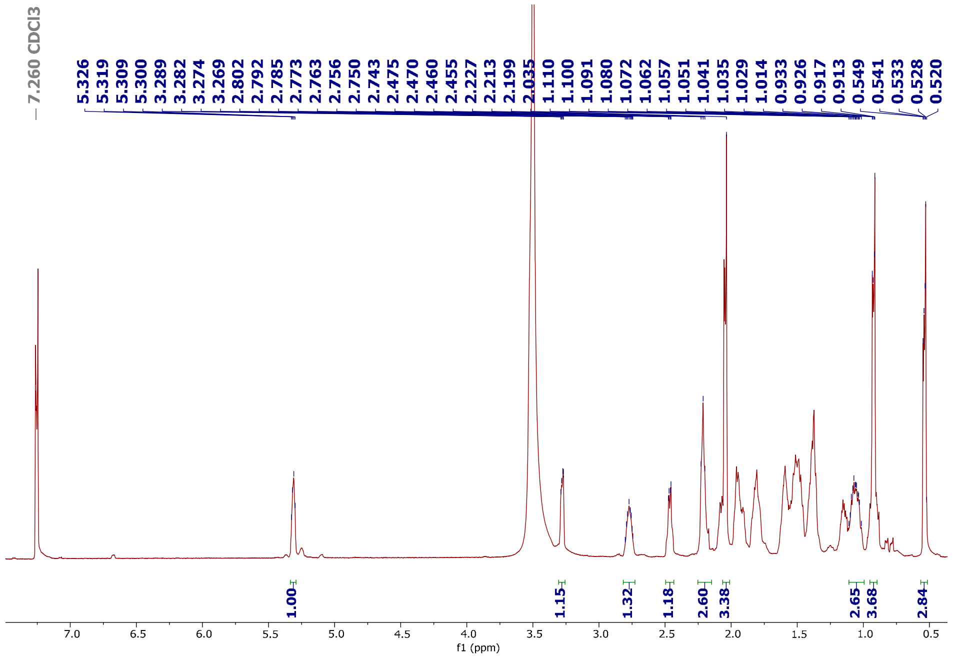

Figure S7: ^{1}H NMR spectrum of compound **14** ($CDCl_3/CD_3OD$, 600 MHz)

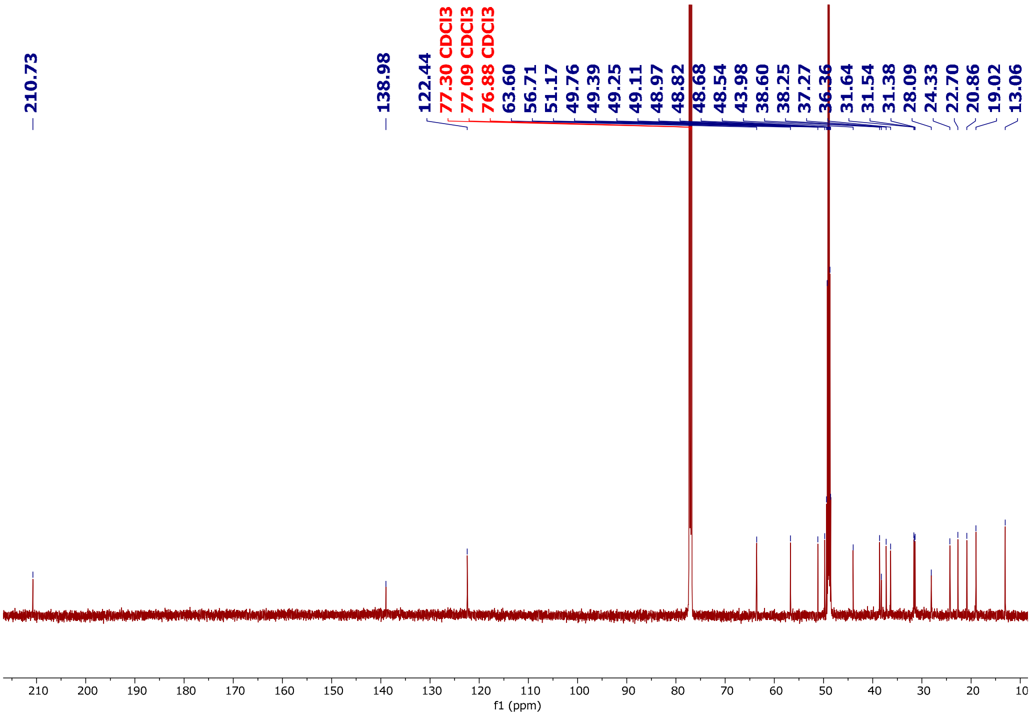

Figure S8: ^{13}C NMR spectrum of compound **14** ($CDCl_3/CD_3OD$, 150 MHz)

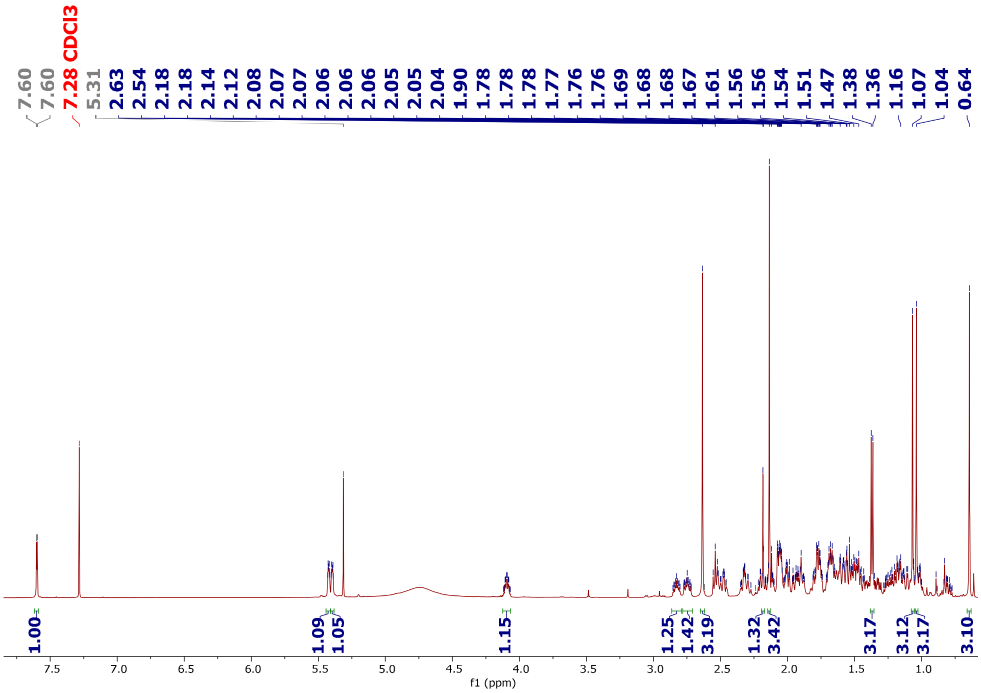

Figure S9: ^{1}H NMR spectrum of compounds **3** and **15** ($CDCl_3$, 600 MHz)

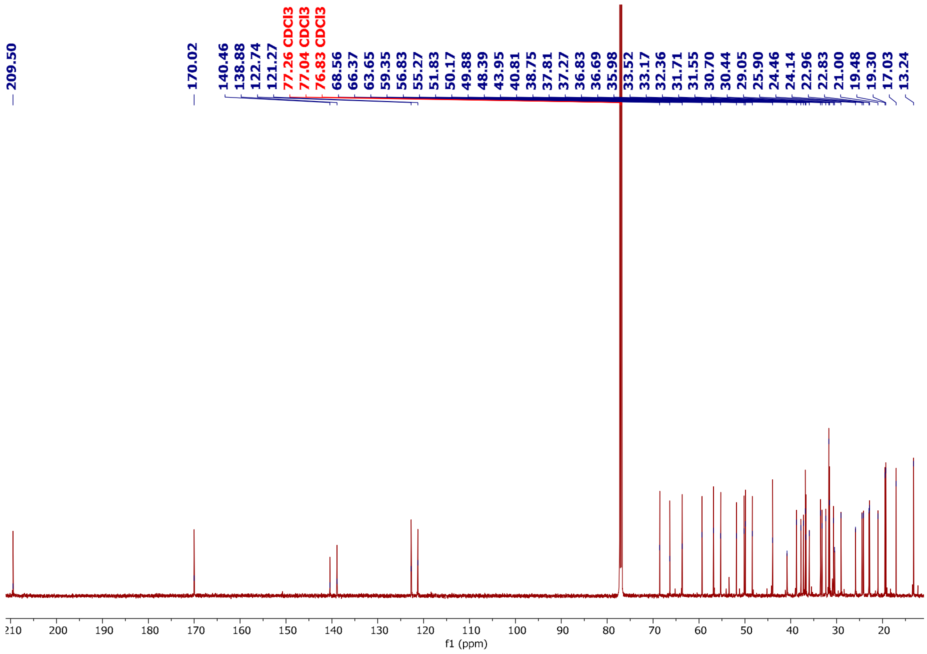

Figure S10: ^{13}C NMR spectrum compounds **3** and **15** ($CDCl_3$, 150 MHz)

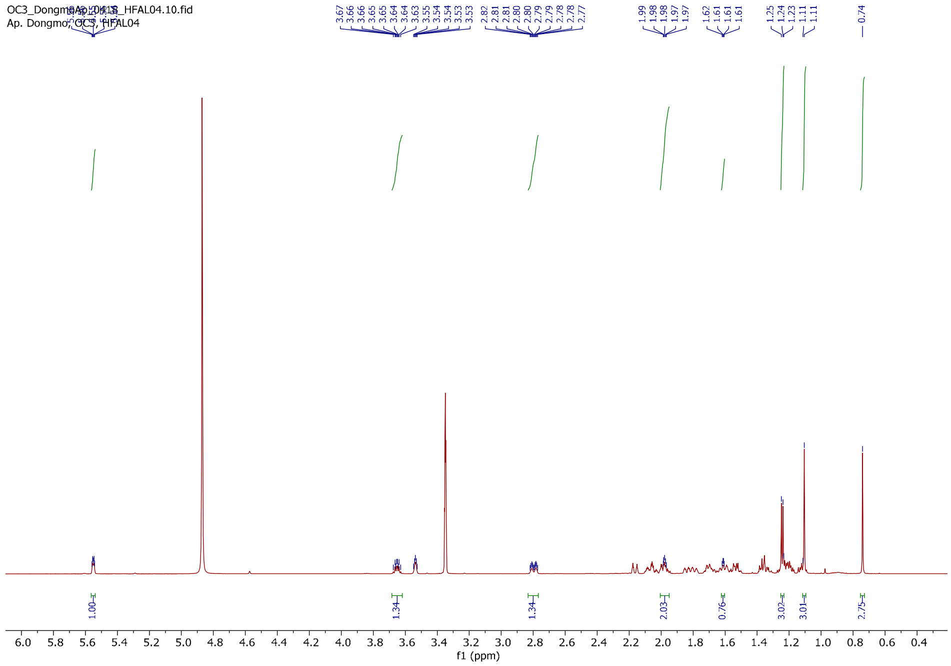

Figure S11: ^{1}H NMR spectrum of compound **16** ($CDCl_3/CD_3OD$, 600 MHz)

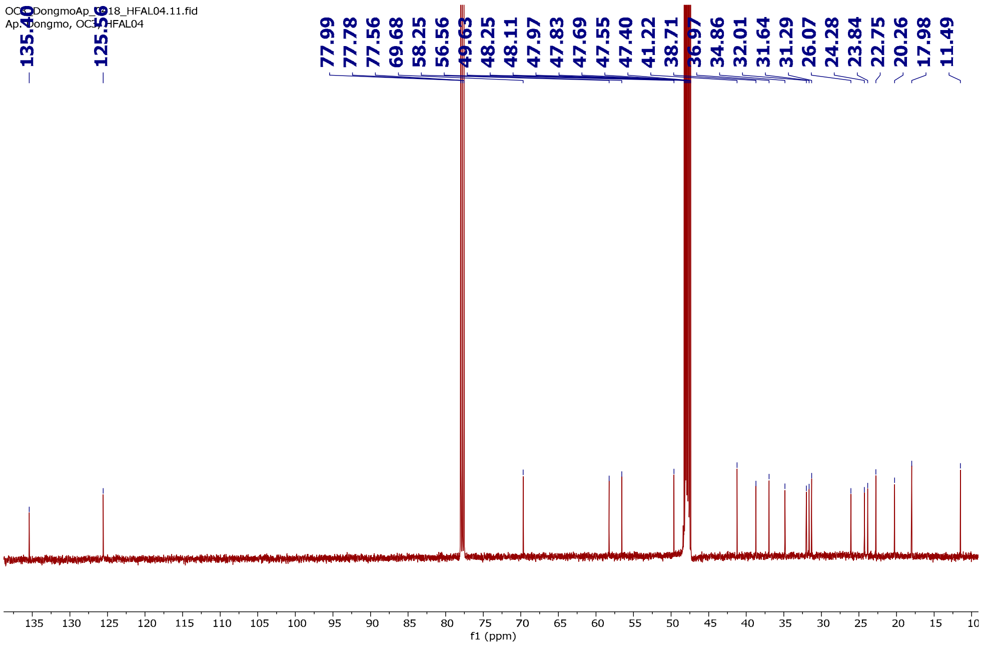

Figure S12: ^{13}C NMR spectrum of compound **16** ($CDCl_3/CD_3OD$, 150 MHz)

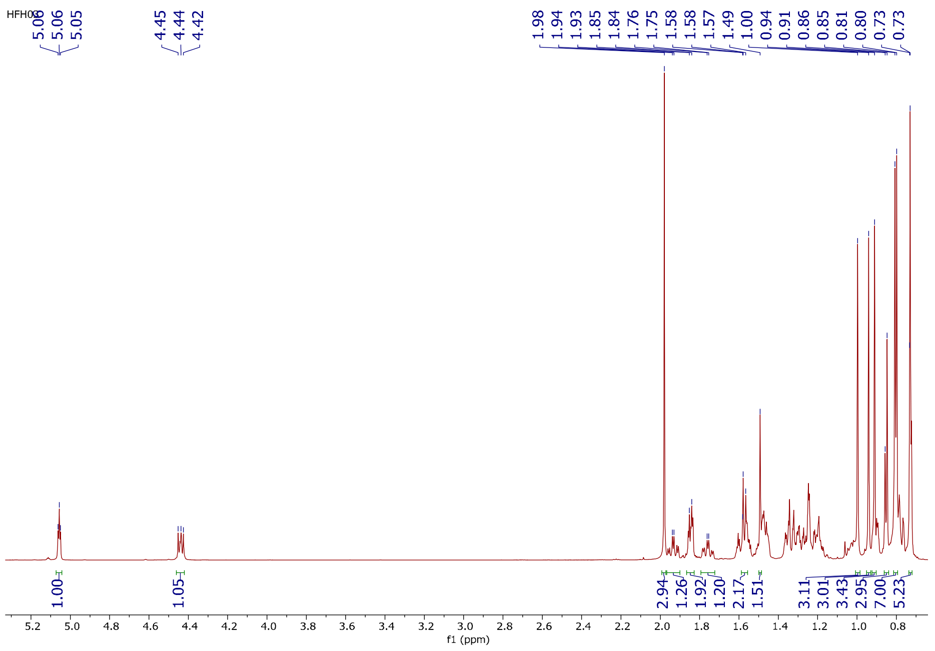

Figure S13: ^{1}H NMR spectrum of compound **18** ($CDCl_3$, 600 MHz)

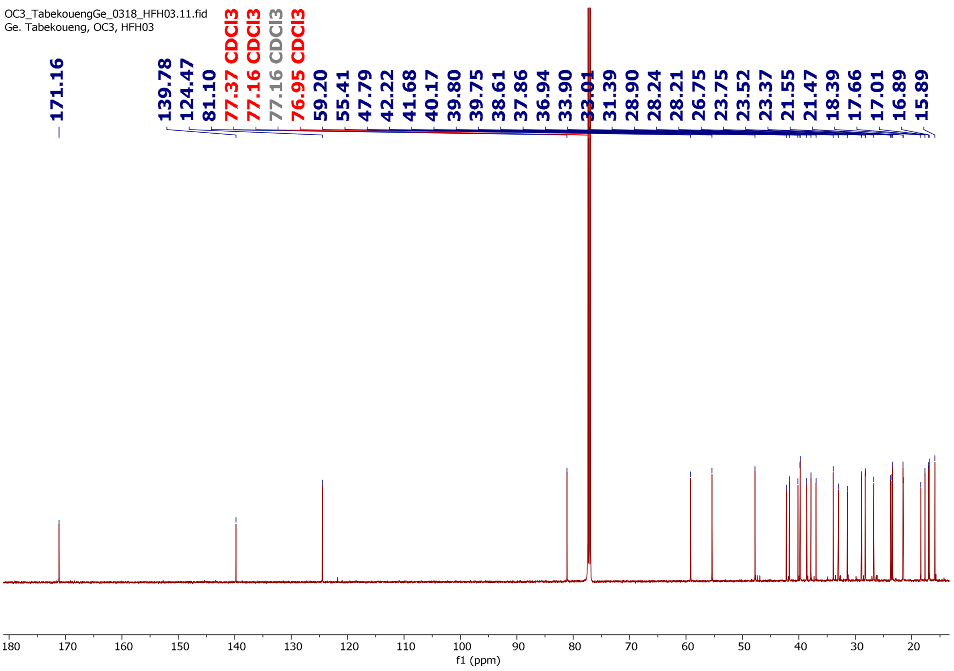

Figure S14: ^{13}C NMR spectrum of compound **18** ($CDCl_3$, 150 MHz)

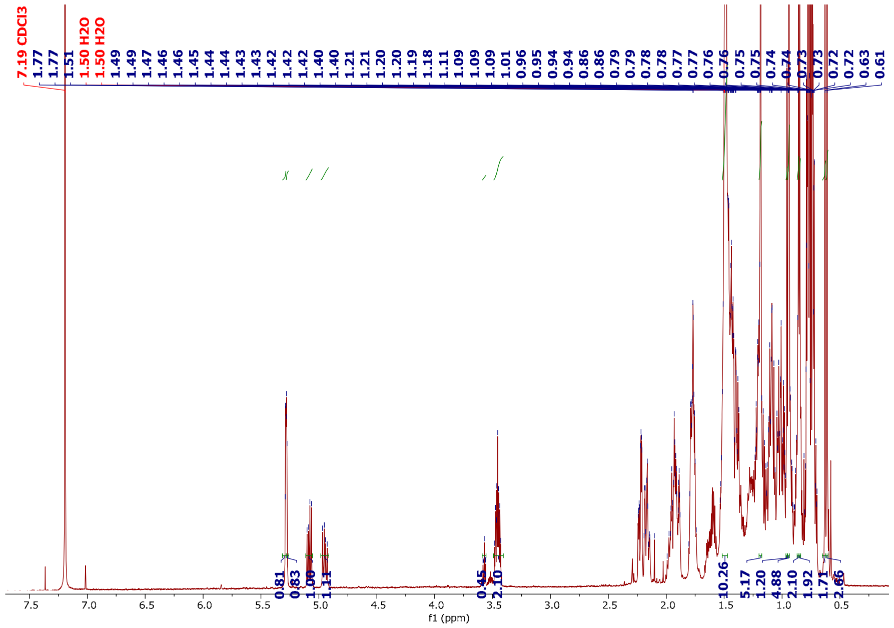

Figure S15: ^{1}H NMR spectrum of compounds **19** and **20** ($CDCl_3$, 600 MHz)

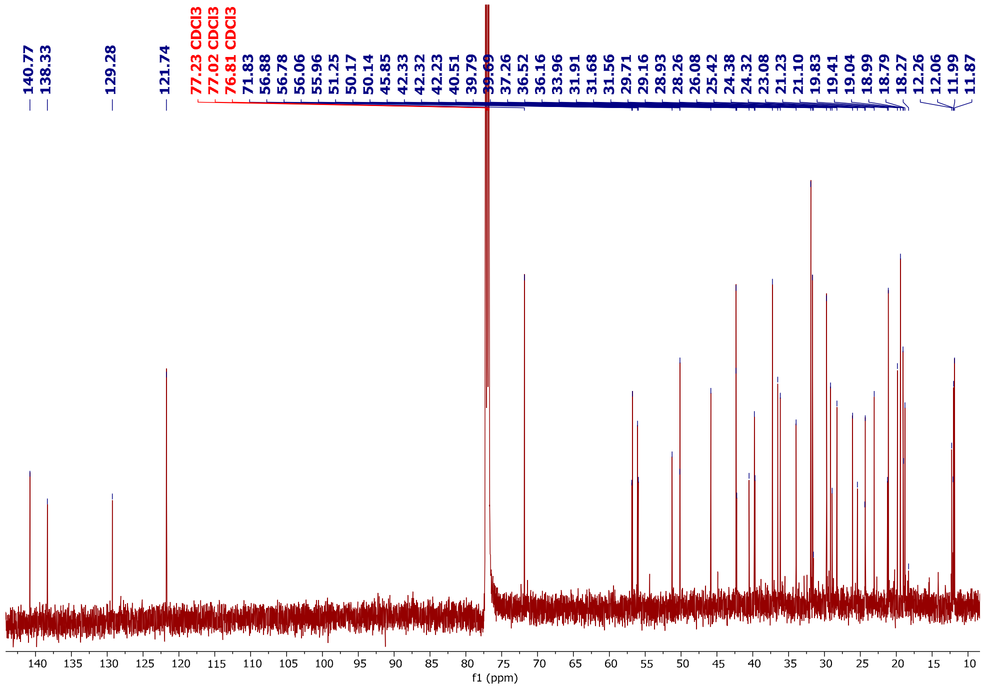

Figure S16 : ^{13}C NMR spectrum of compounds **19** and **20** ($CDCl_3$, 150 MHz)

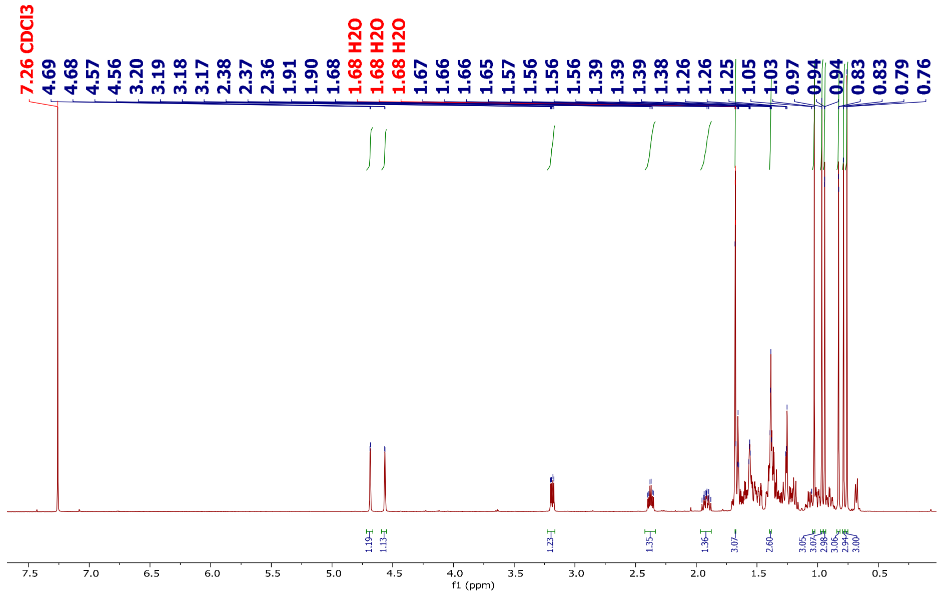

Figure S17: ^{1}H NMR spectrum of compound **21** ($CDCl_3$, 600 MHz)

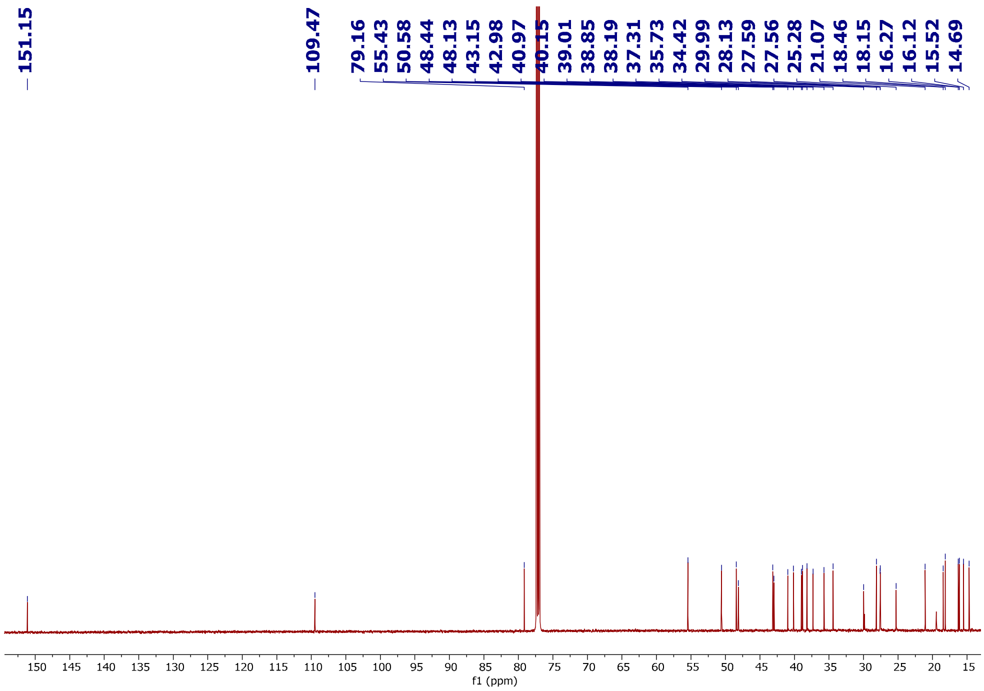

Figure S18: ^{13}C NMR spectrum of compound **21** ($CDCl_3$, 600 MHz)

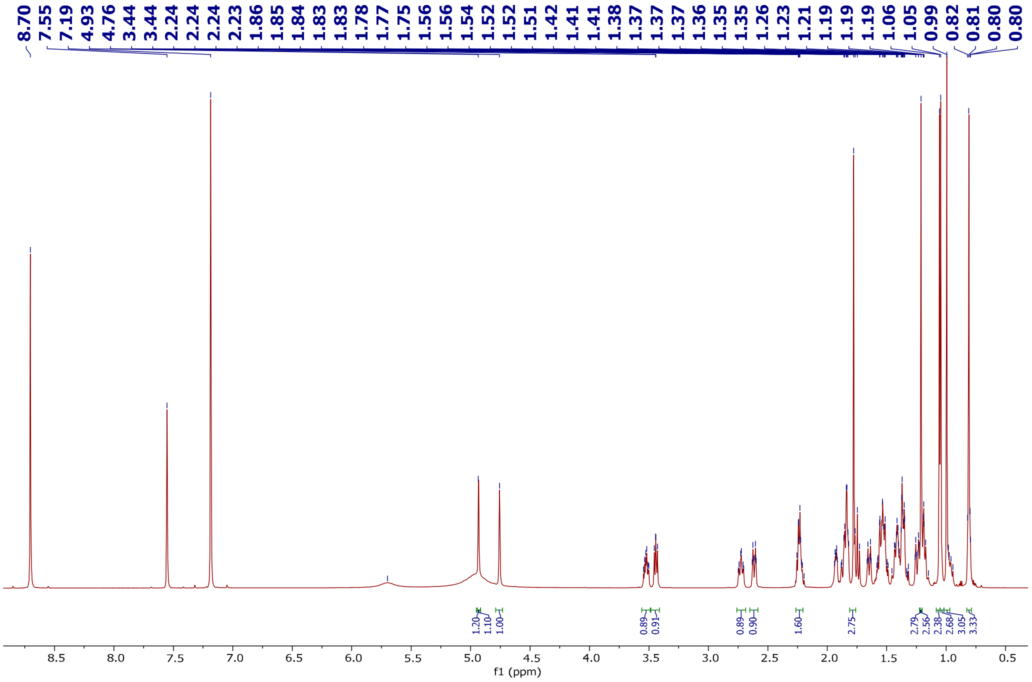

Figure S19: ^{1}H NMR spectrum of compound **22** (Pyridine-d_5, 600 MHz)

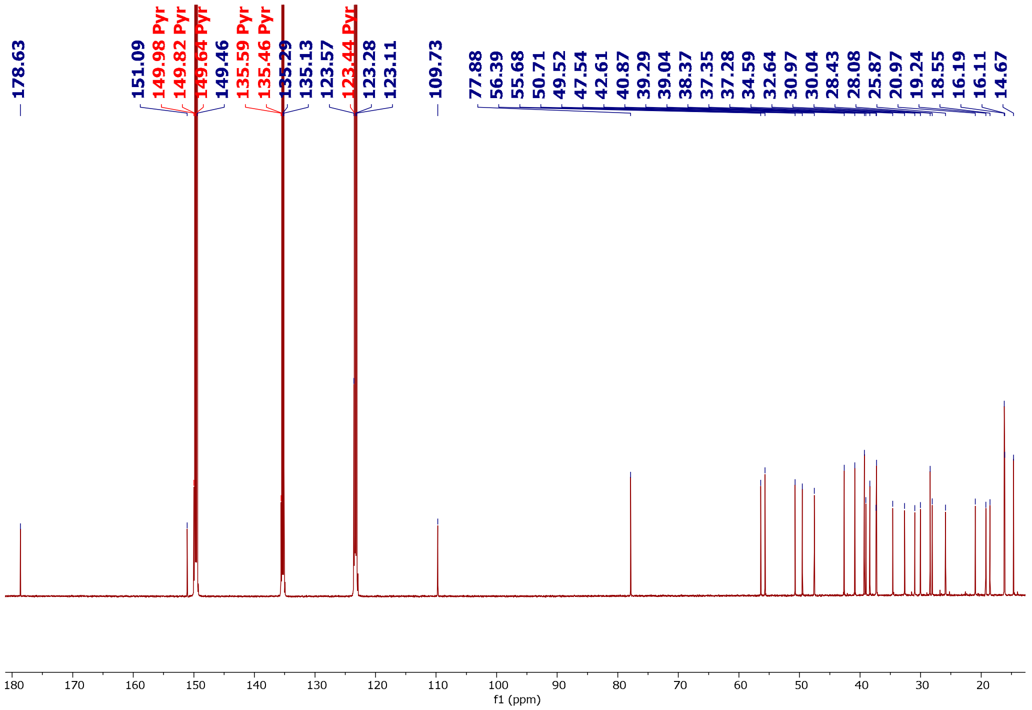

Figure S20: ^{1}H NMR spectrum of compound **22** (Pyridine-d_5, 600 MHz)

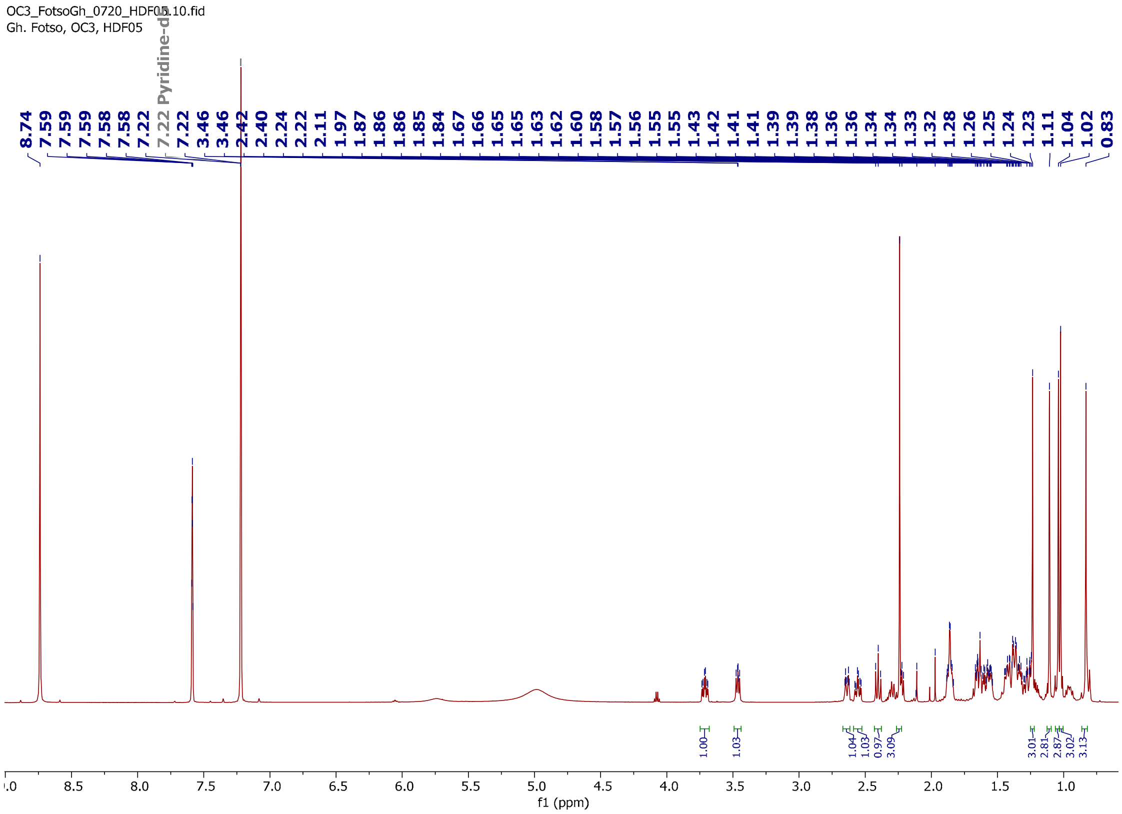

Figure S21: ^{1}H NMR spectrum of compound **23** (Pyridine-d_5, 600 MHz)

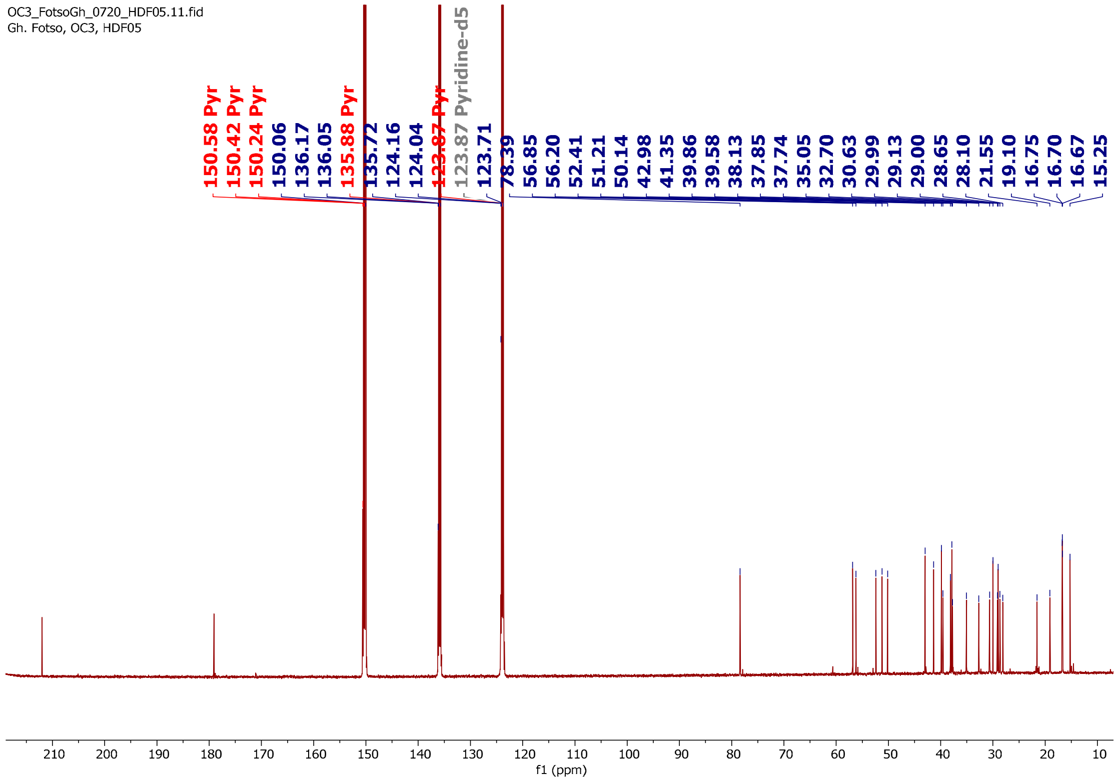

Figure S22: ^{13}C NMR spectrum of compound **23** (Pyridine-d_5, 150 MHz)

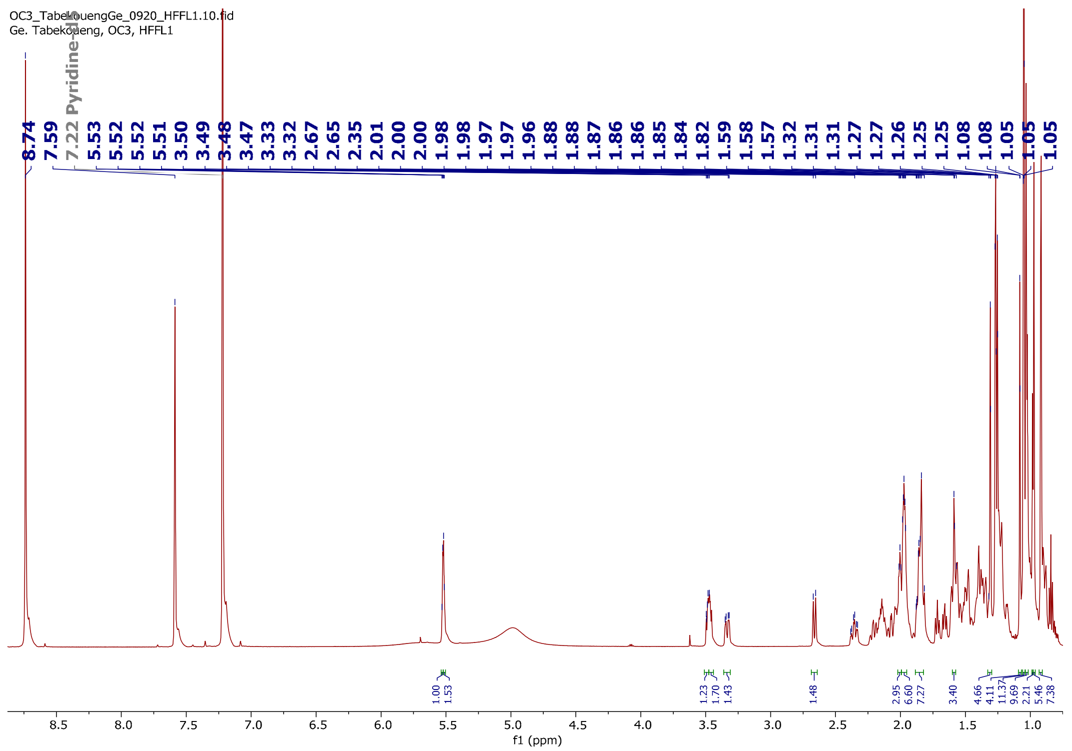

Figure S23: ^{1}H NMR spectrum of compounds **24** and **25** (Pyridine-d_5, 600 MHz)

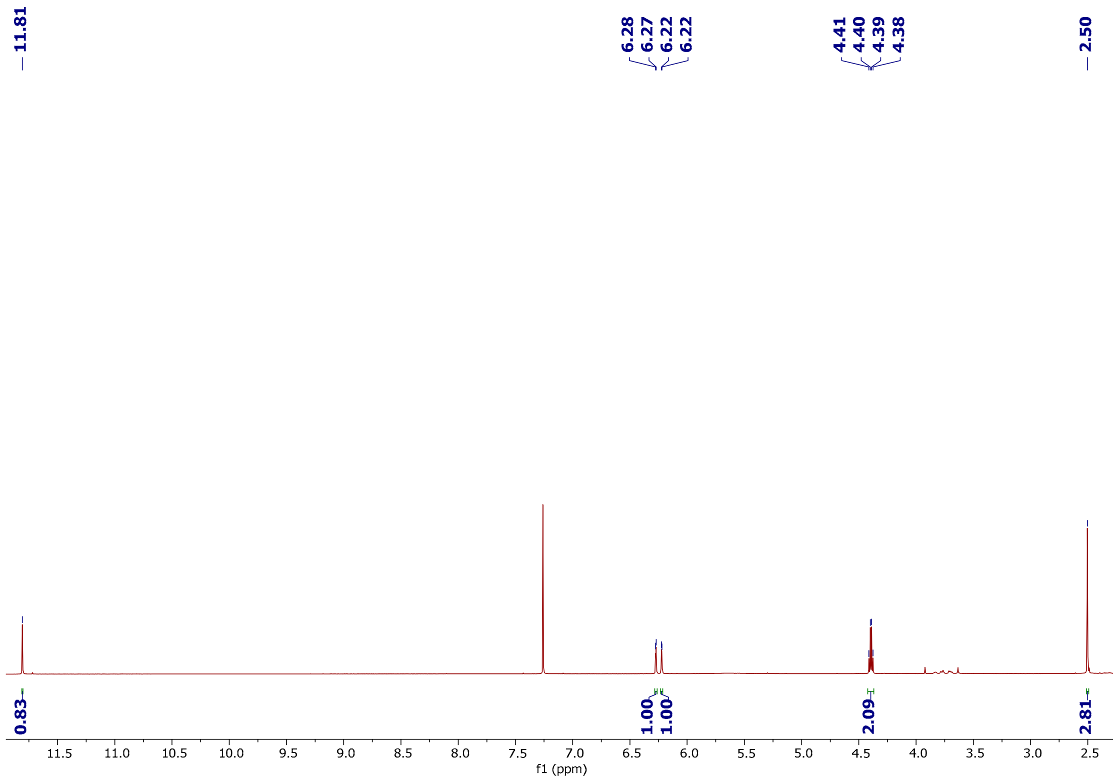

Figure S24: ^{1}H NMR spectrum of compound **26** ($CDCl_3$, 600 MHz)

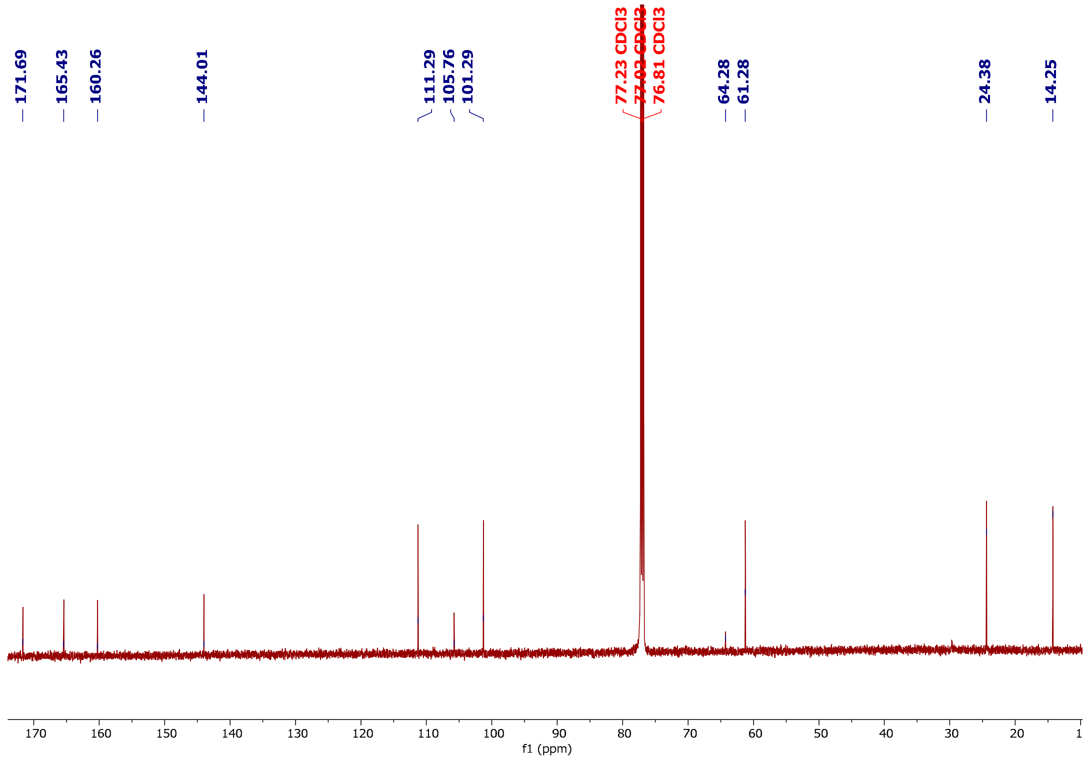

Figure S25: ^{13}C NMR spectrum of compound **26** ($CDCl_3$, 150 MHz)

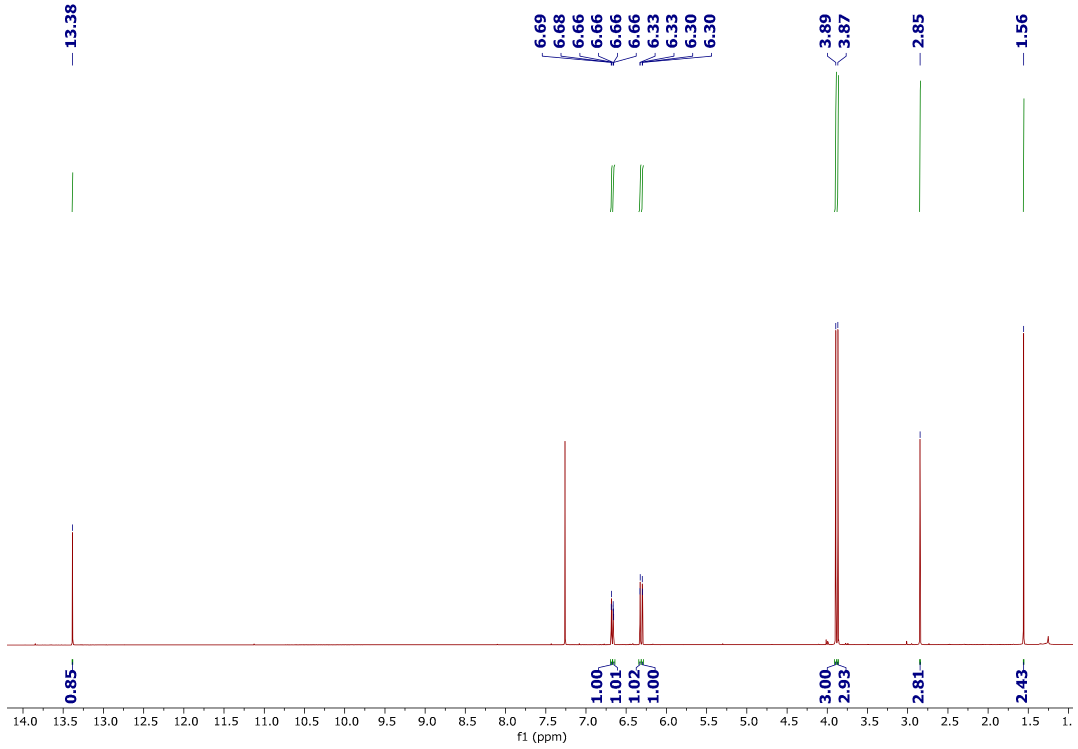

Figure S26: ^{1}H NMR spectrum of compound **27** ($CDCl_3$, 600 MHz)

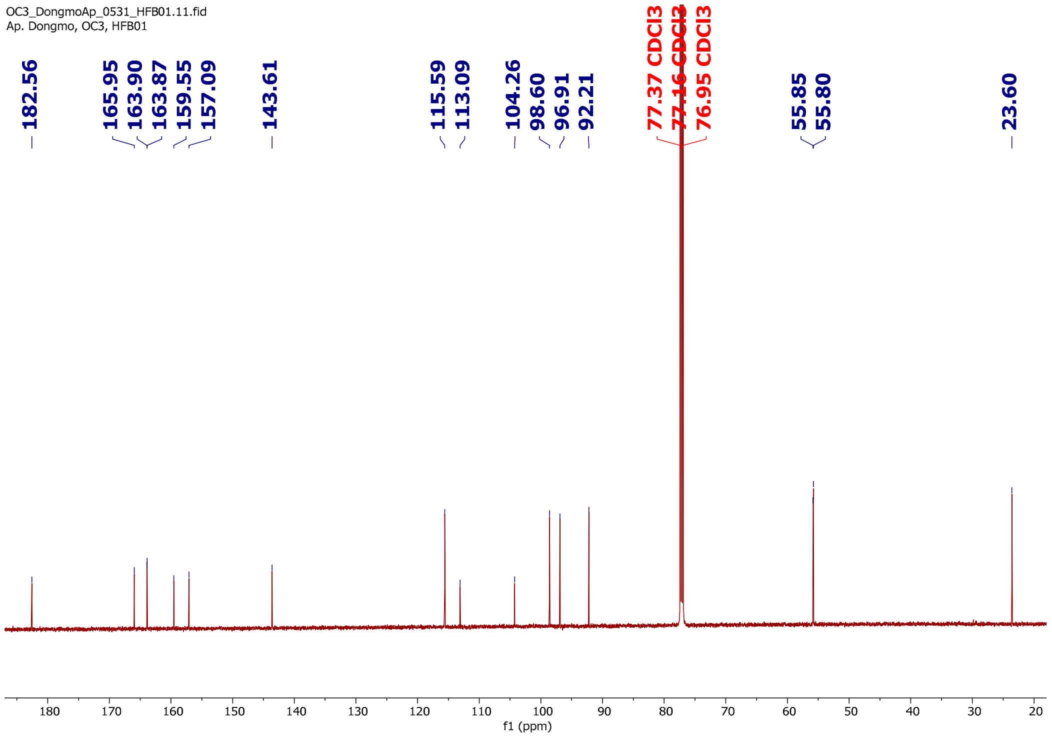

Figure S27: ^{13}C NMR spectrum of compound **27** ($CDCl_3$, 150 MHz)

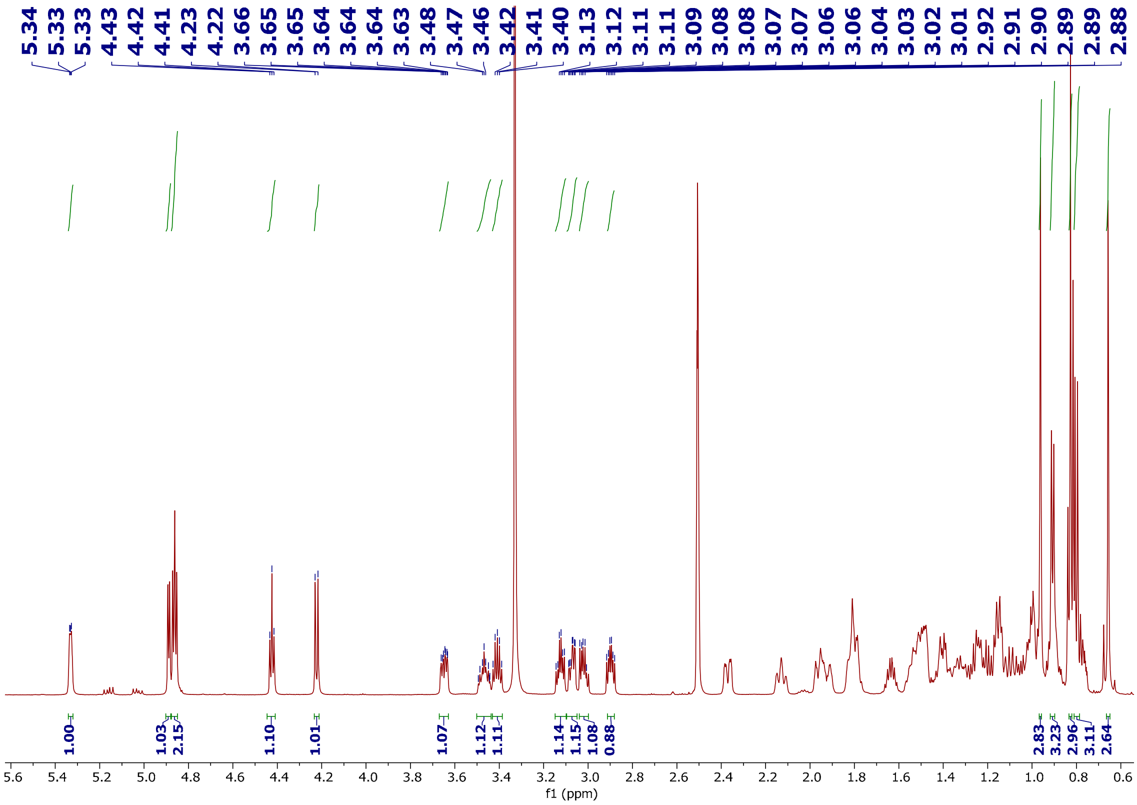

Figure S28: ^{1}H NMR spectrum of compound **28** (DMSO-d_6, 600 MHz)

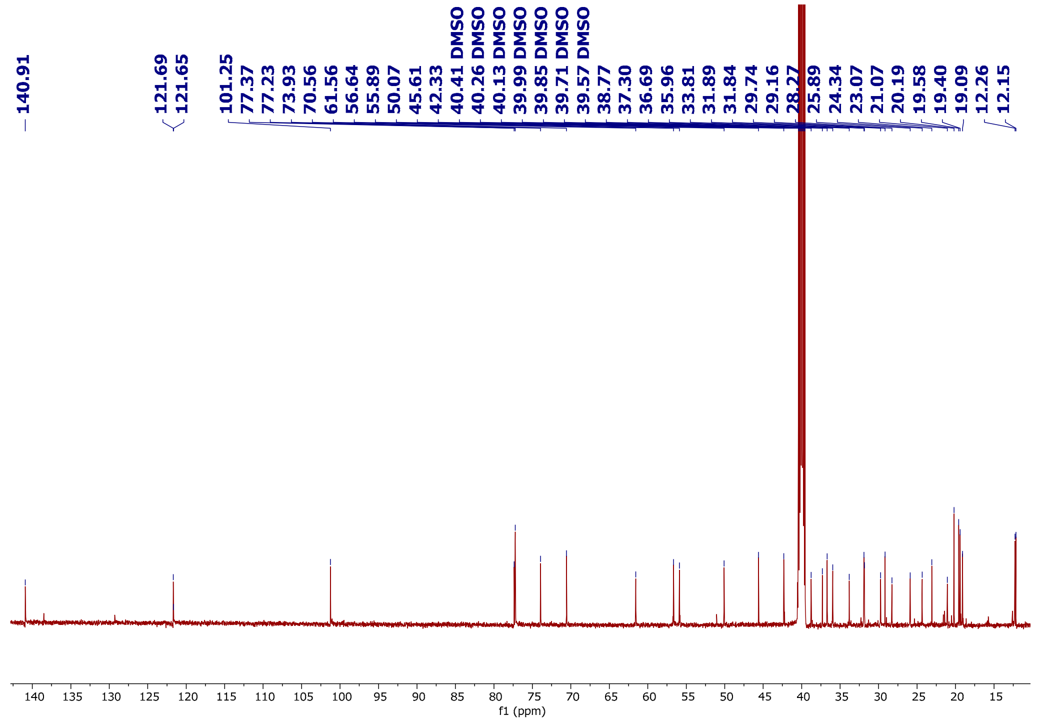

Figure S29: ^{13}C NMR spectrum of compound **28** (DMSO-d_6, 600 MHz)

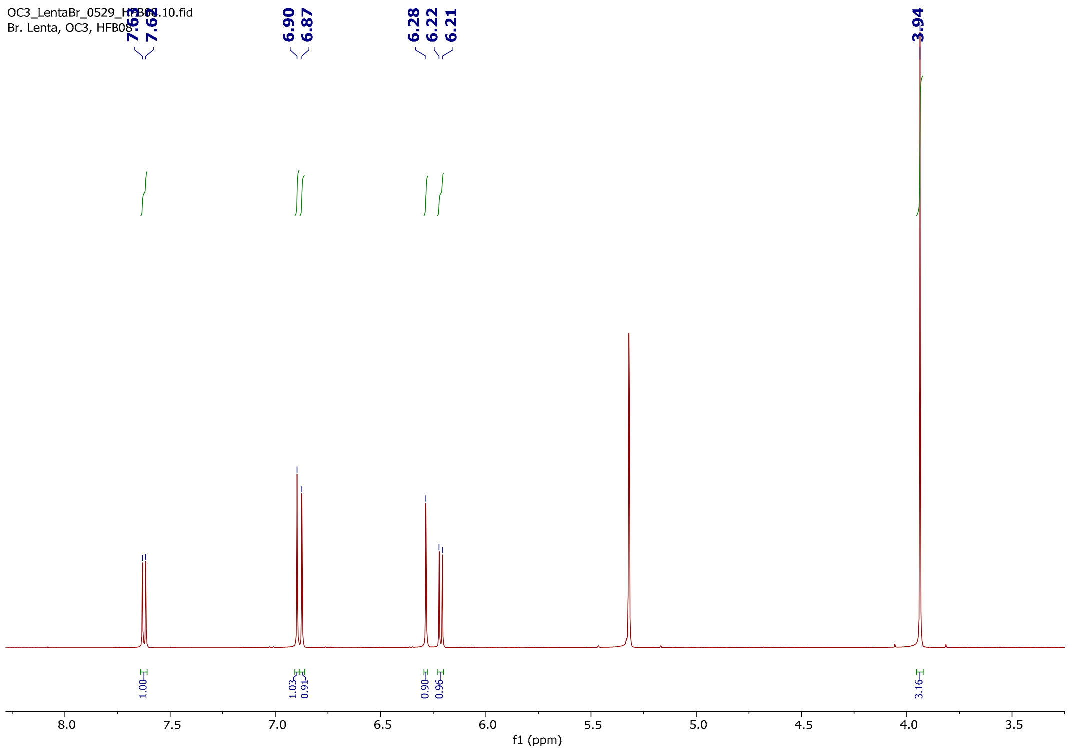

Figure S30: ^{1}H NMR spectrum of compound **29** (CD_2Cl_2, 600 MHz)

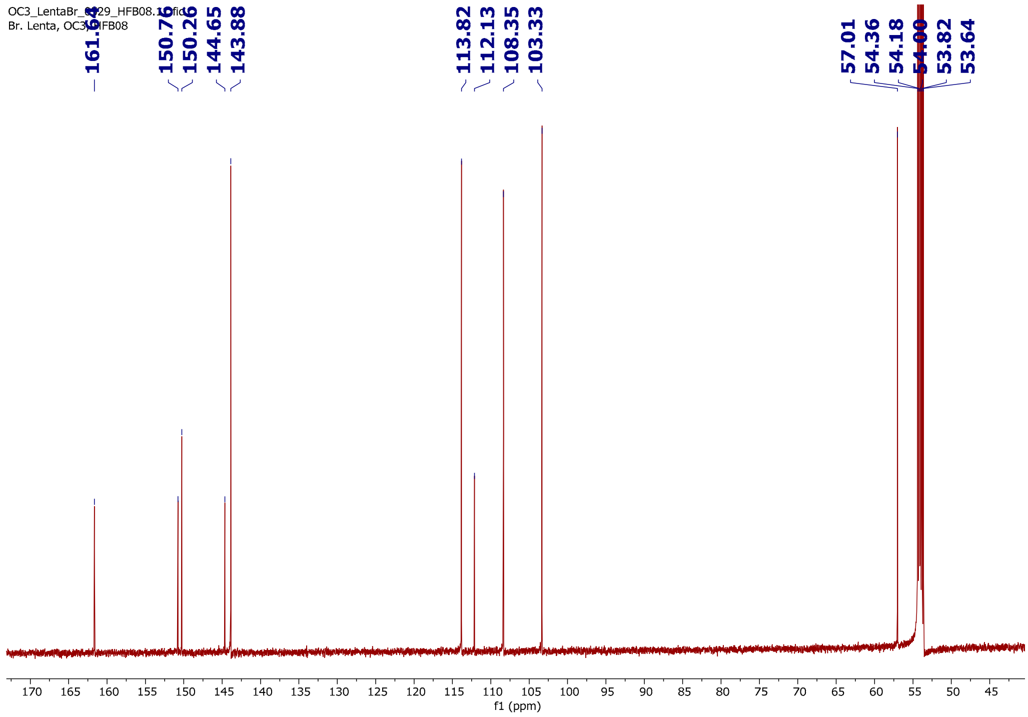

Figure S31: ^{13}C NMR spectrum of compound **29** (CD_2Cl_2, 150 MHz)

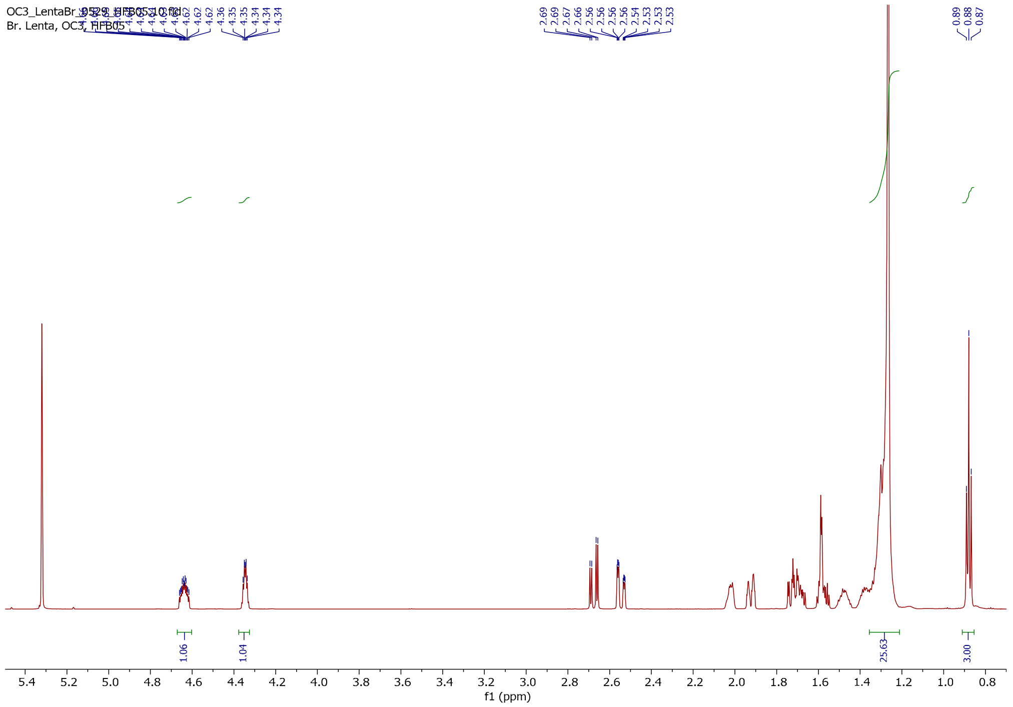

Figure S32: ^{1}H NMR spectrum of compound **30** (CD_2Cl_2, 600 MHz)

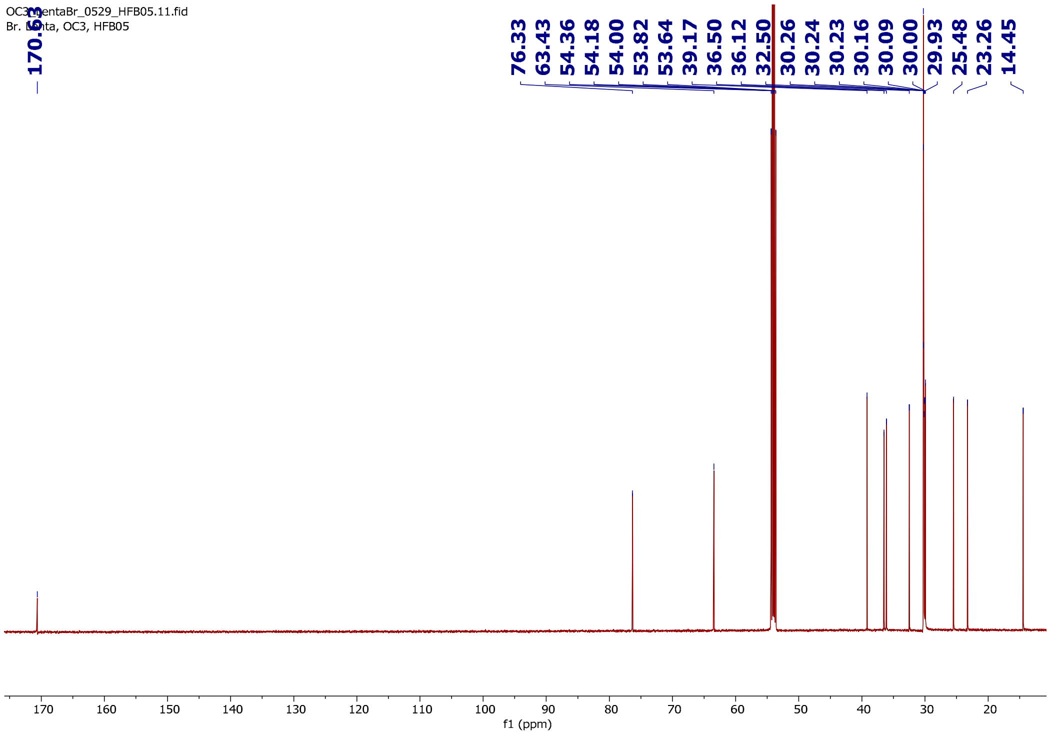

Figure S33: ^{13}C NMR spectrum of compound **30** (CD_2Cl_2, 150 MHz)

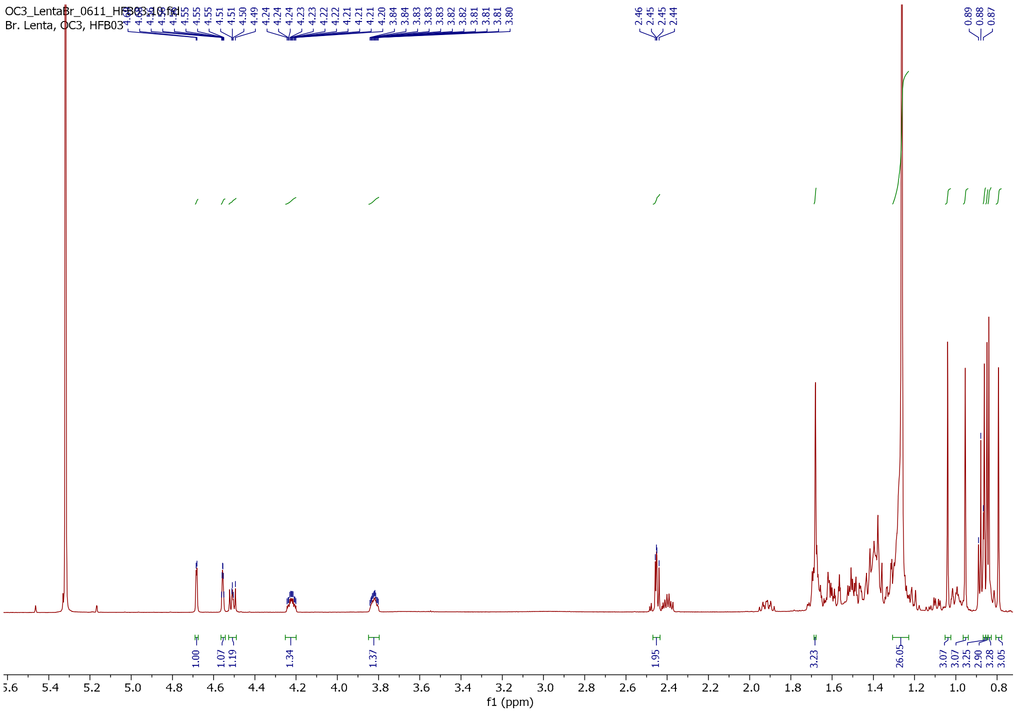

Figure S34 : ^{1}H NMR spectrum of compound **31** (CD_2Cl_2, 600 MHz)

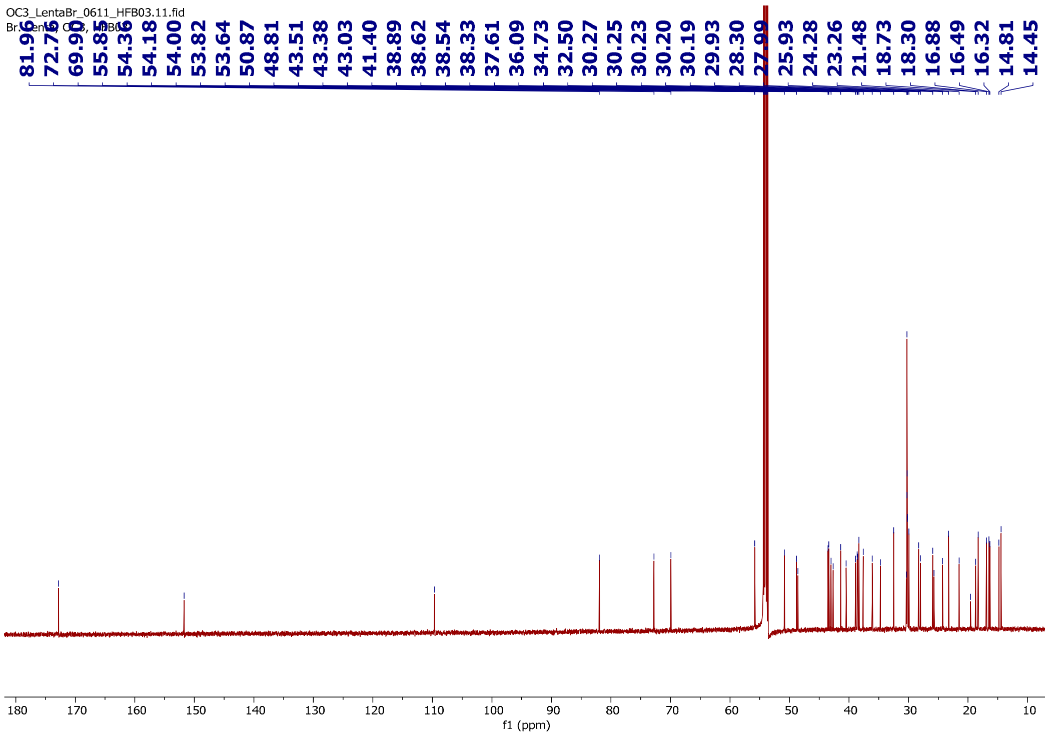

Figure S35: ^{13}C NMR spectrum of compound **31** (CD_2Cl_2, 150 MHz)

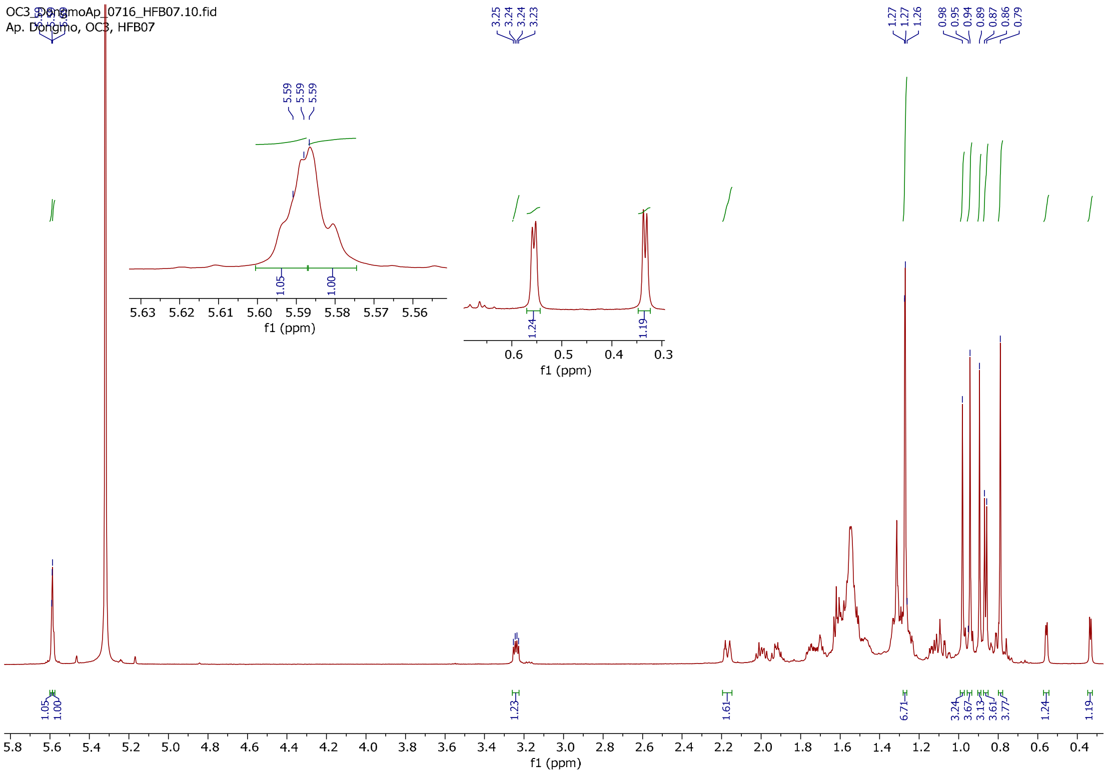

Figure S36: ^{1}H NMR spectrum of compound **32** (CD_2Cl_2, 600 MHz)

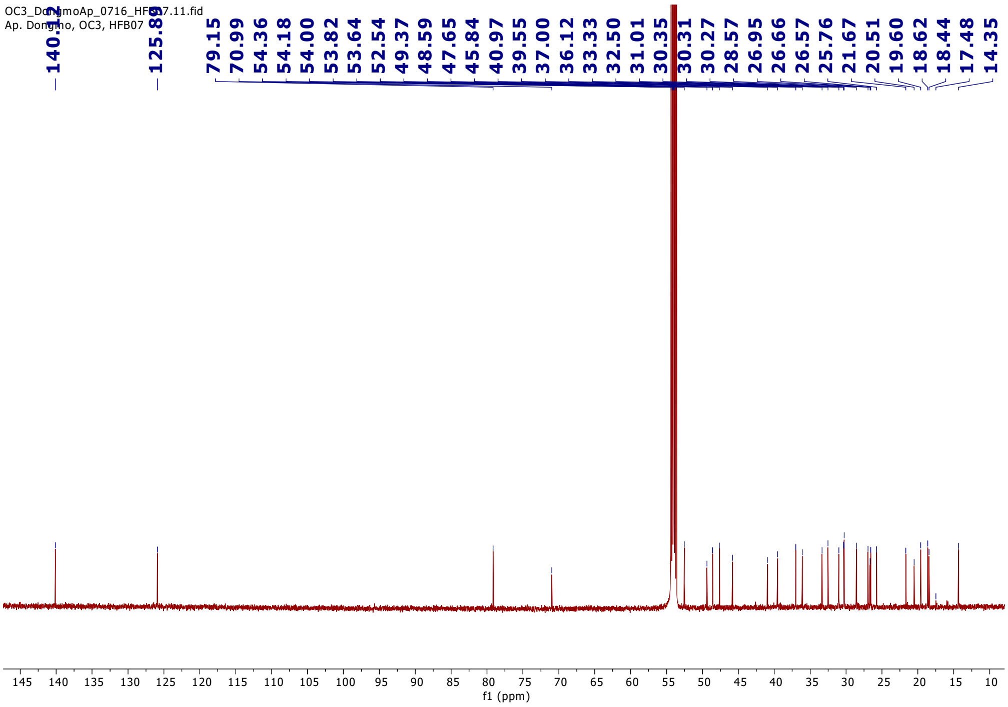

Figure S37: ^{13}C NMR spectrum of compound **32** (CD_2Cl_2, 150 MHz)

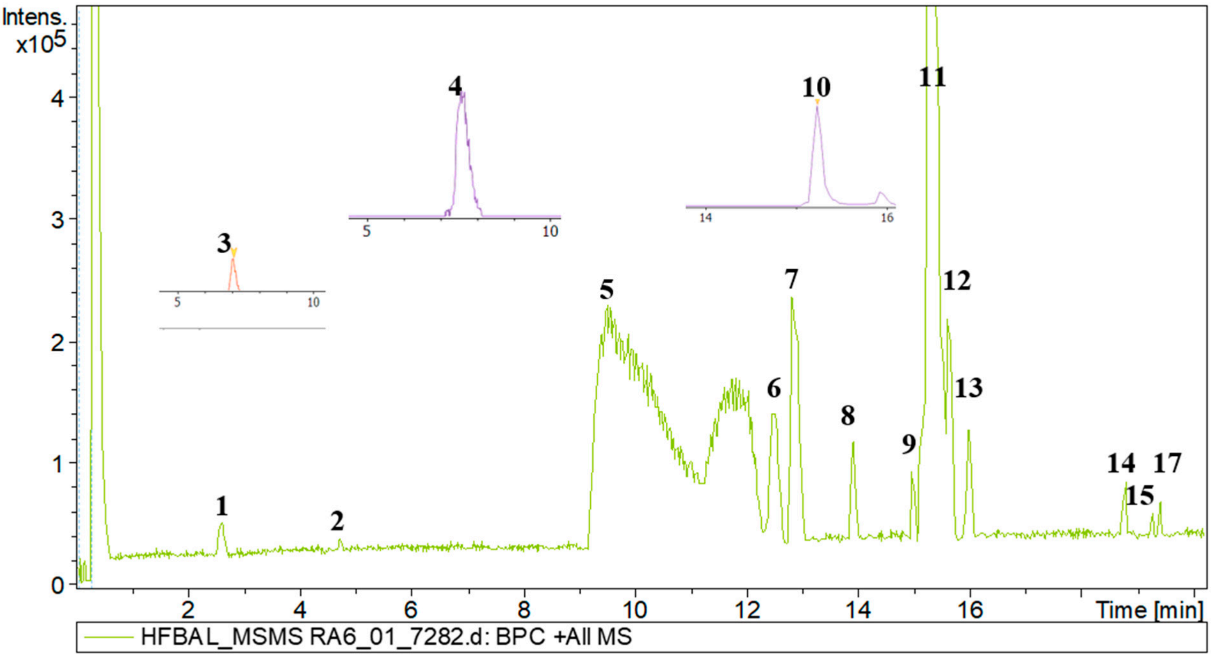

Figure S38: Base Peak Chromatogram (BPC) of the alkaloid fraction from the stem bark of *H. floribunda*

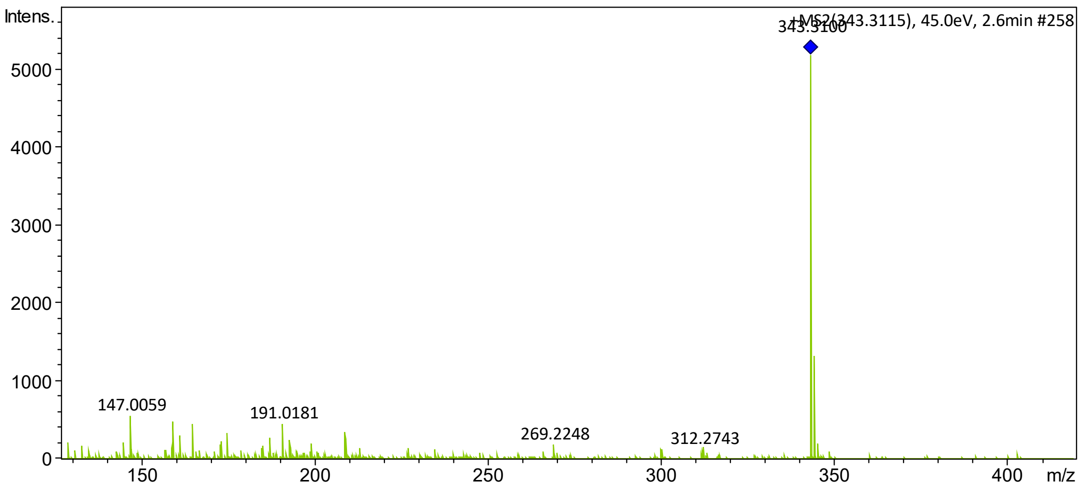

Figure S39: MS/MS fragmentation pattern of isoconessimine (**1**)

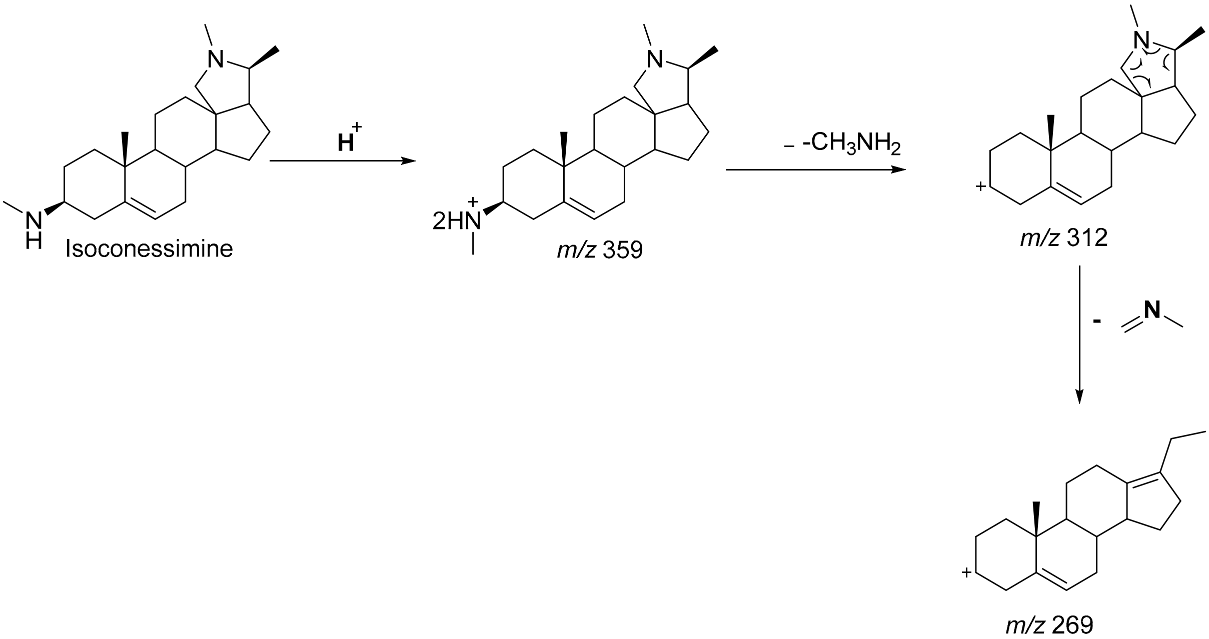

Scheme S1: MS/MS fragmentation of isoconessimine (**1**)

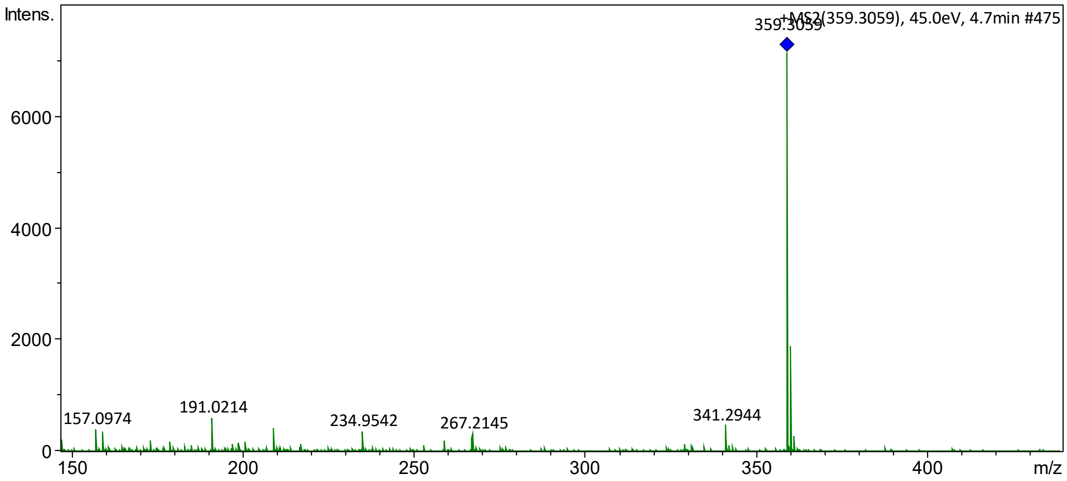

Figure S40: MS/MS spectrum of regholarrhenin D (**2**)

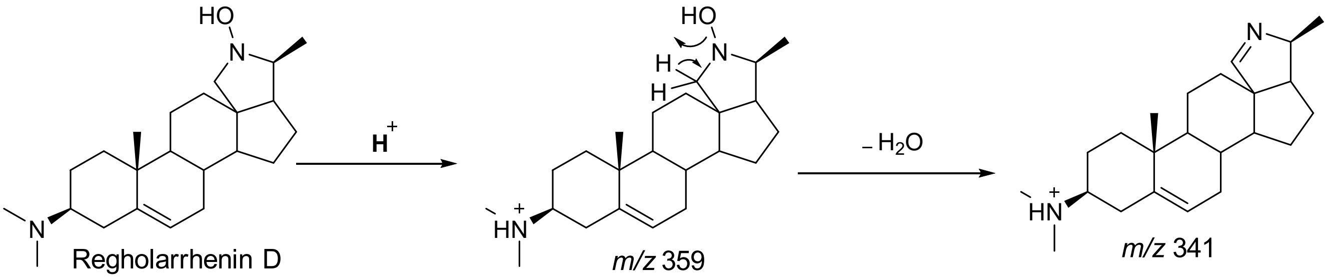

Scheme S2: MS/MS fragmentation of regholarrhenine D (**2**)

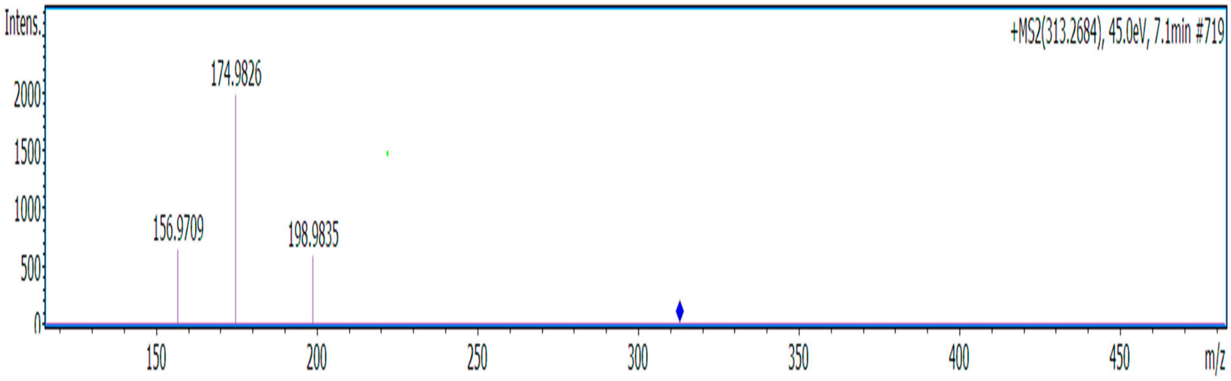

Figure S41: MS/MS fragmentation pattern of irheline (**3**)

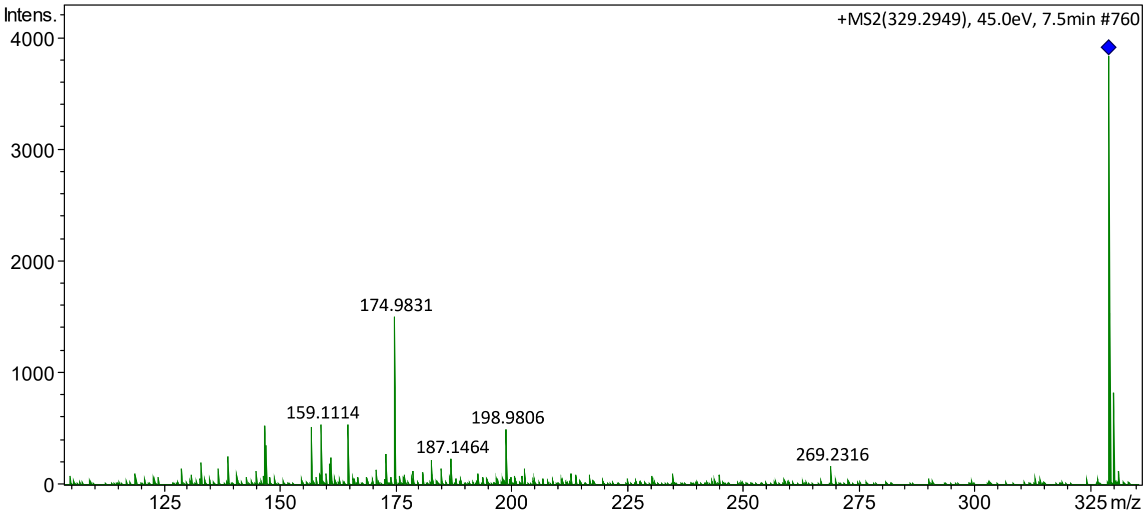

Figure S42: MS/MS spectrum of conimin (**4**)

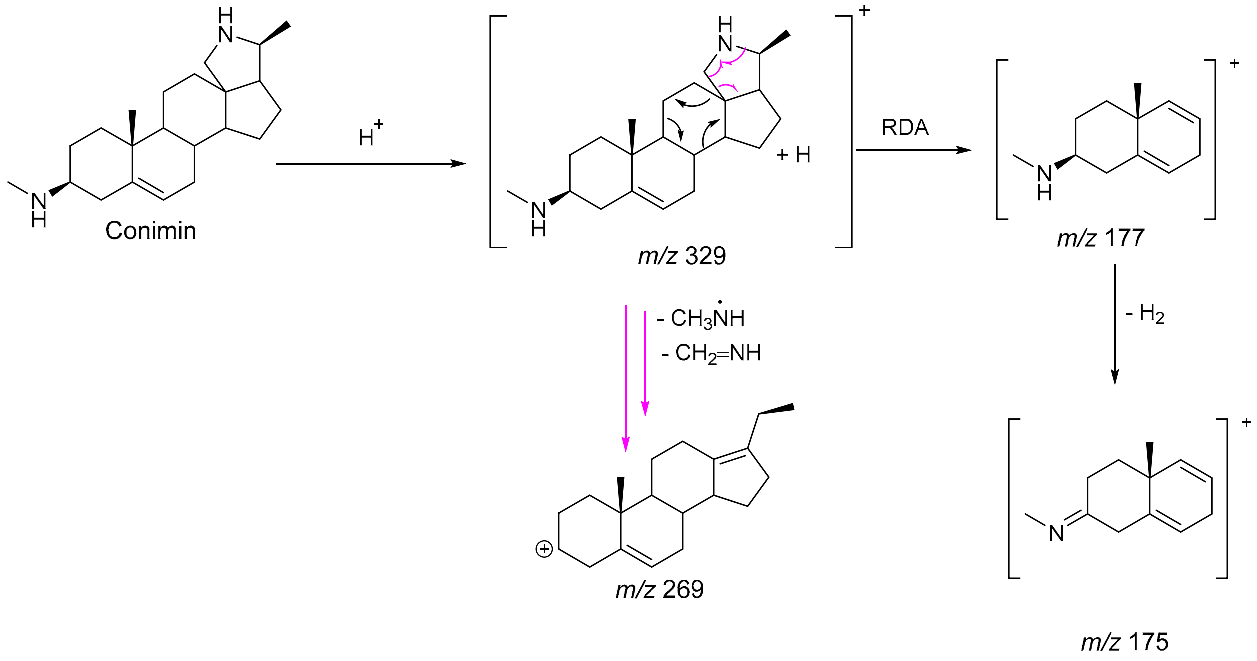

Scheme S3: MS/MS fragmentation of conimin (**4**)

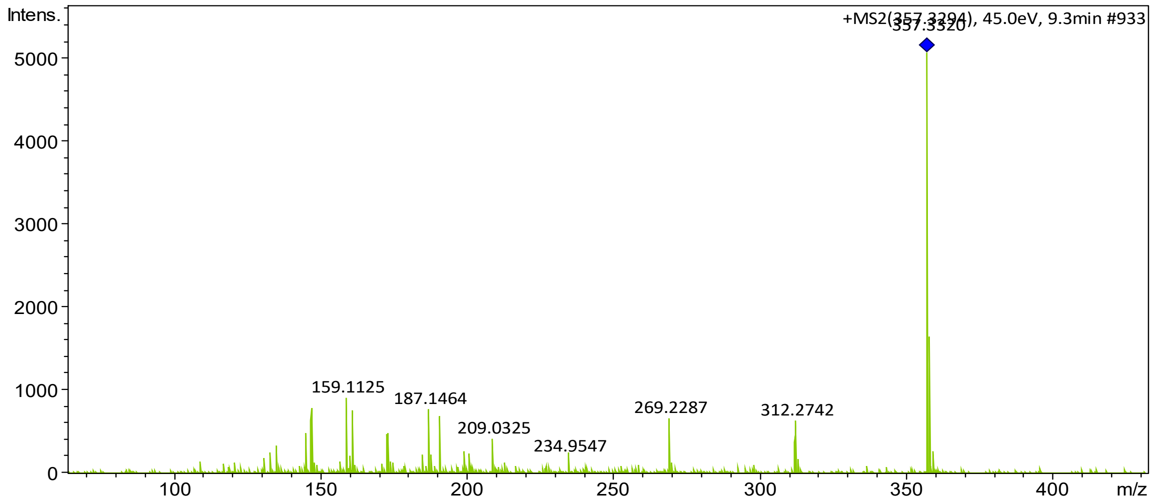

Figure S43 : MS/MS spectrum of conessine (**5**)

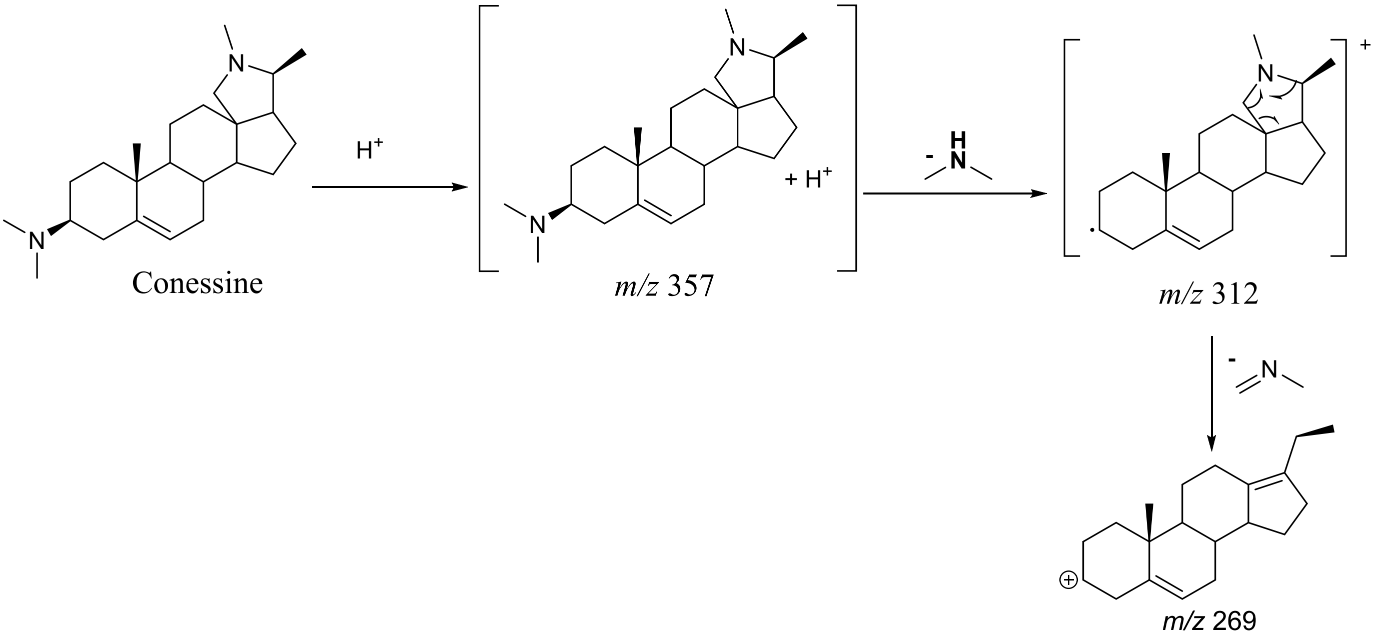

Scheme S4: MS/MS fragmentation of conessine (**5**)

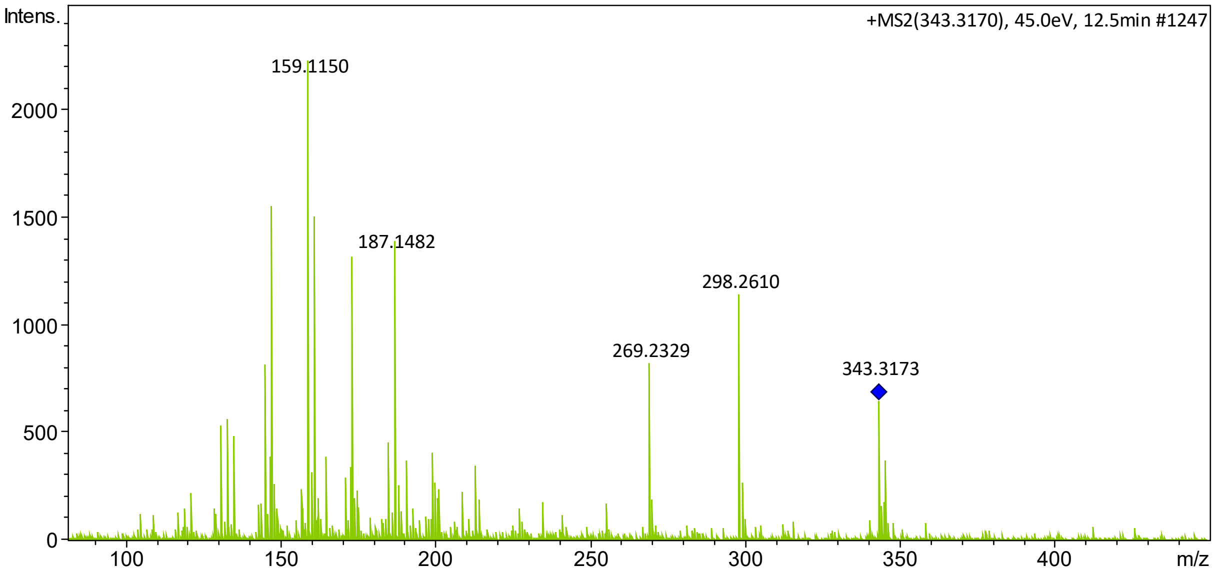

Figure S44 : MS/MS spectrum of conessimine (**6**)

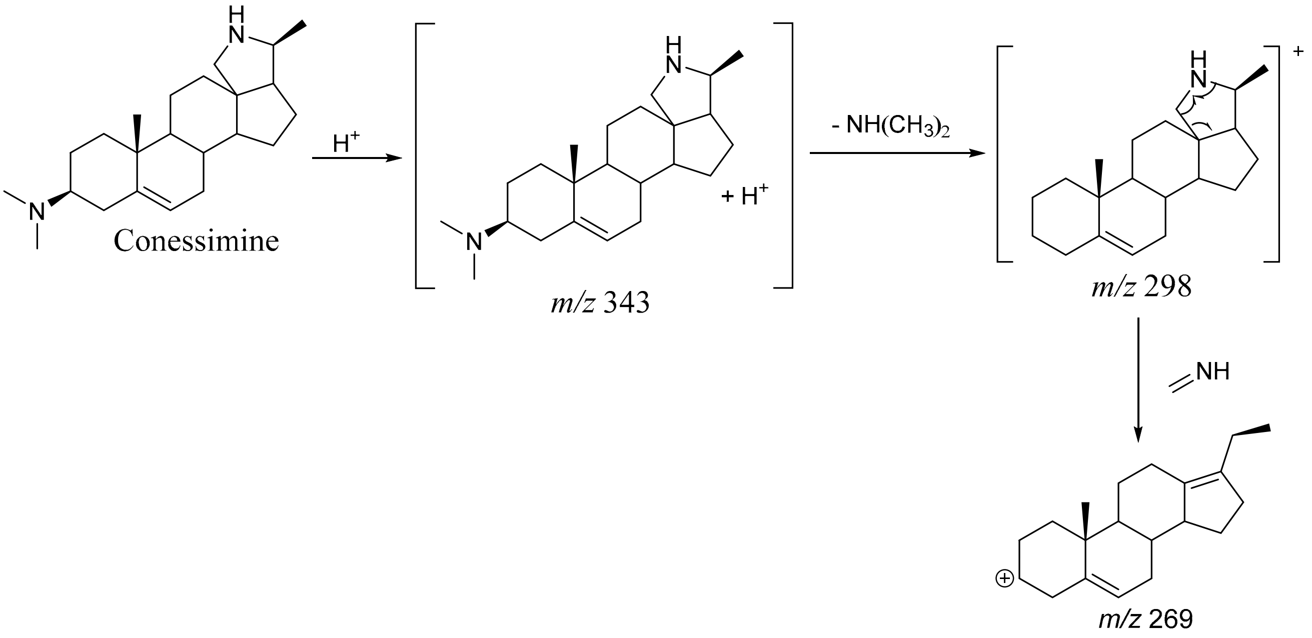

Scheme S5: MS/MS fragmentation of conessimine (**6**)

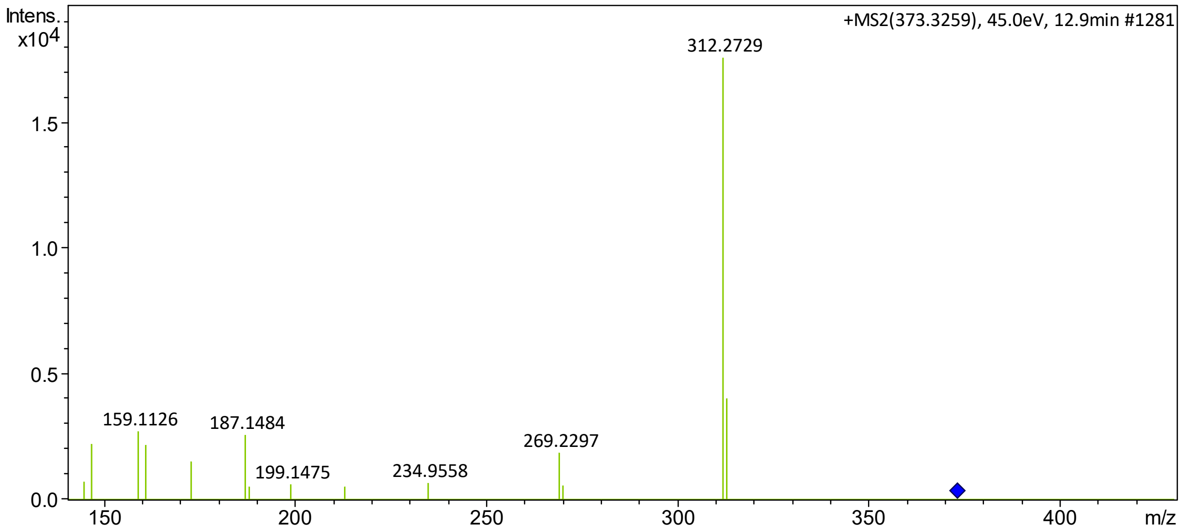

Figure S45: MS/MS spectrum of 7α-hydroxyconessine (**7**)

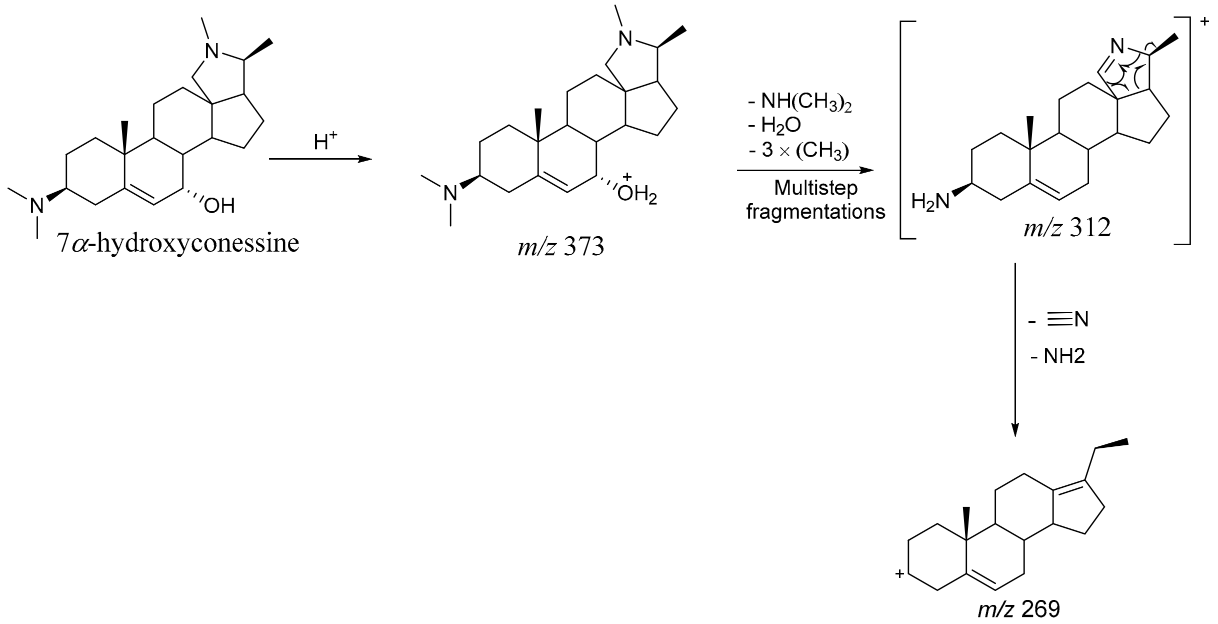

Scheme S6: MS/MS fragmentation of 7α-hydroxyconessine (7)

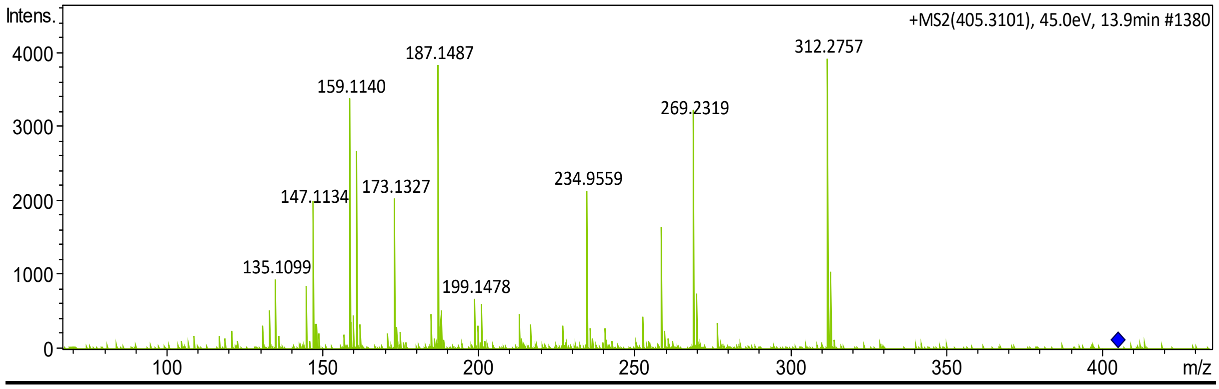

Figure S46: MS/MS spectrum of regholarrhenine E **(8)**

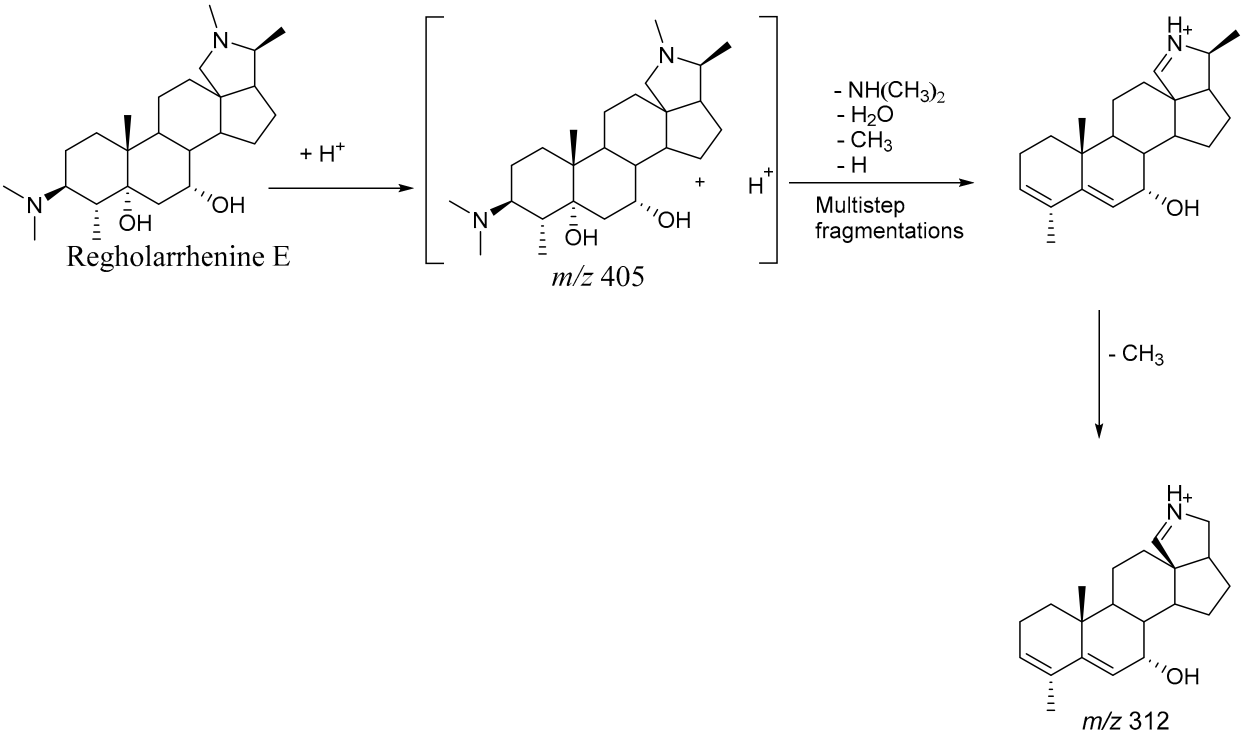

Scheme S7: MS/MS fragmentation of regholarrhenine E (**8**)

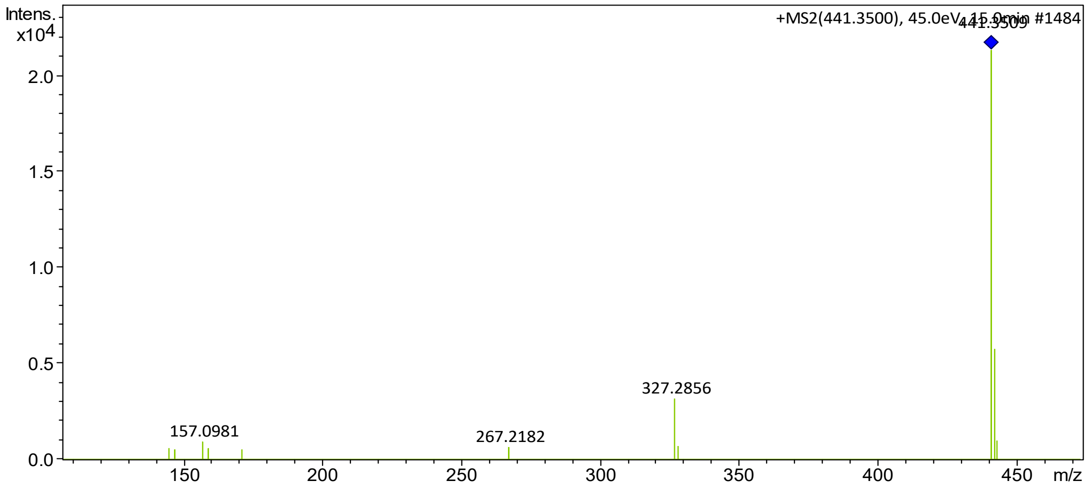

Figure S47: MS/MS spectrum of salignemamide D (**9**)

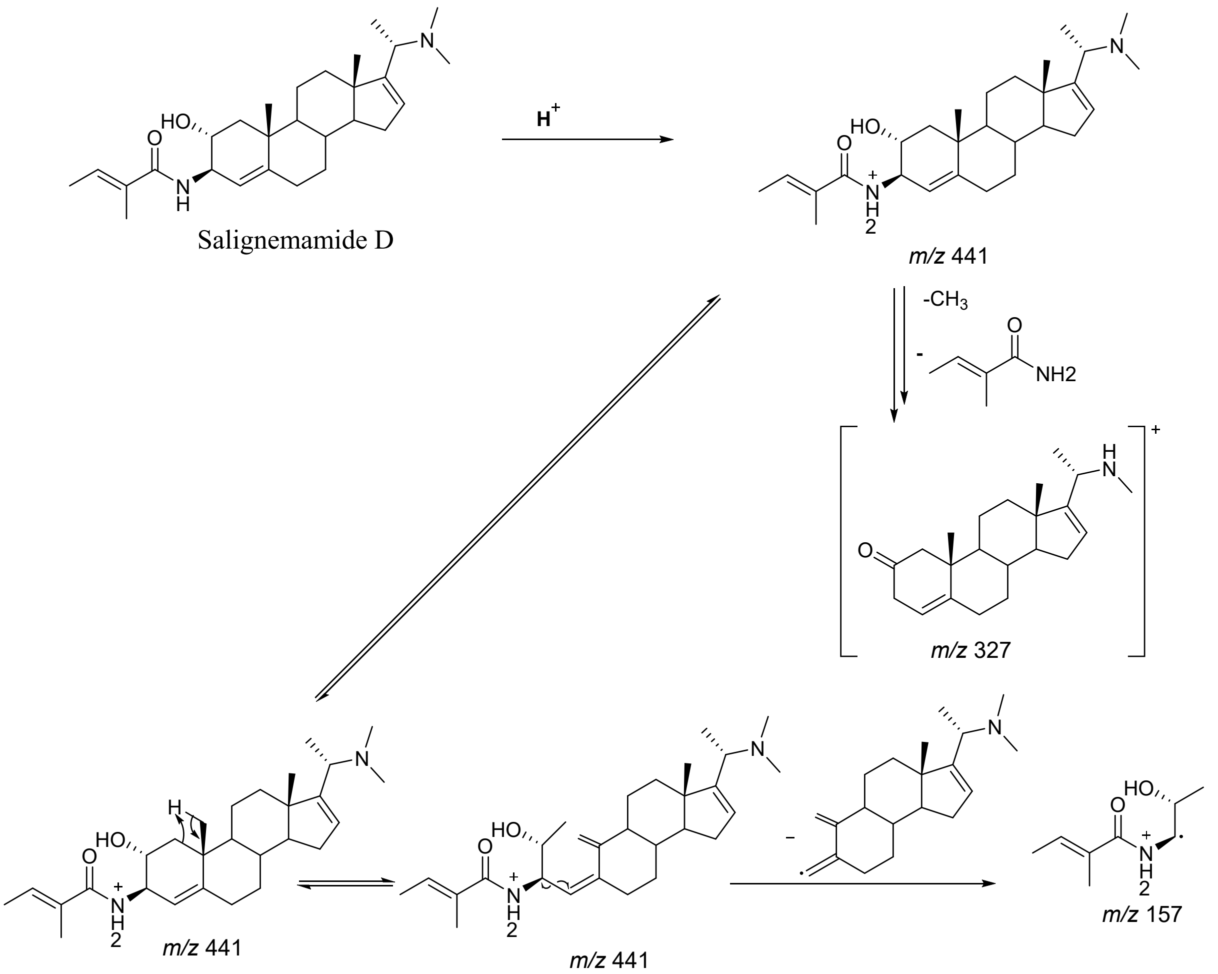

Scheme S8: MS/MS fragmentation of salignemamide D (**9**)

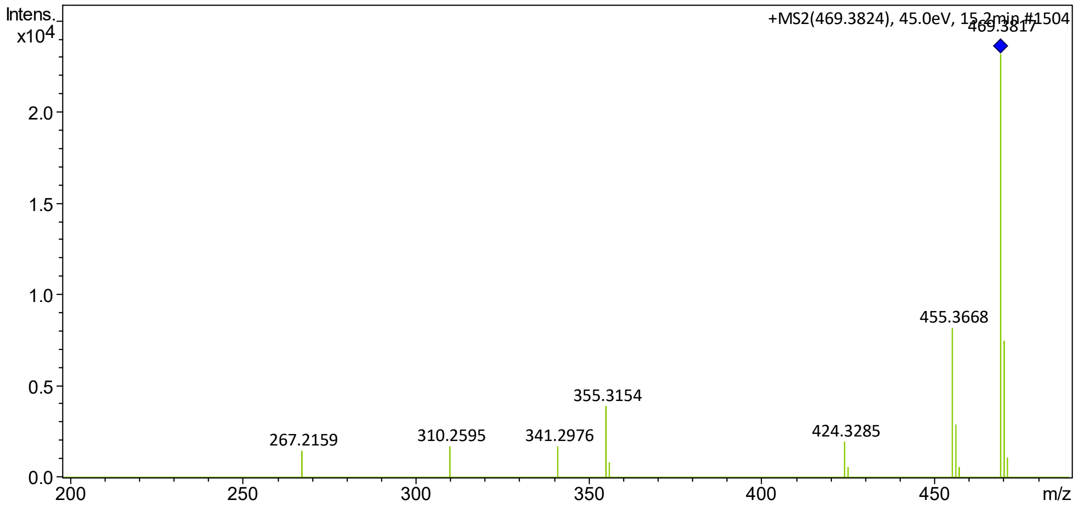

Figure S48 : MS/MS spectrum of holarrhetine (**10**)

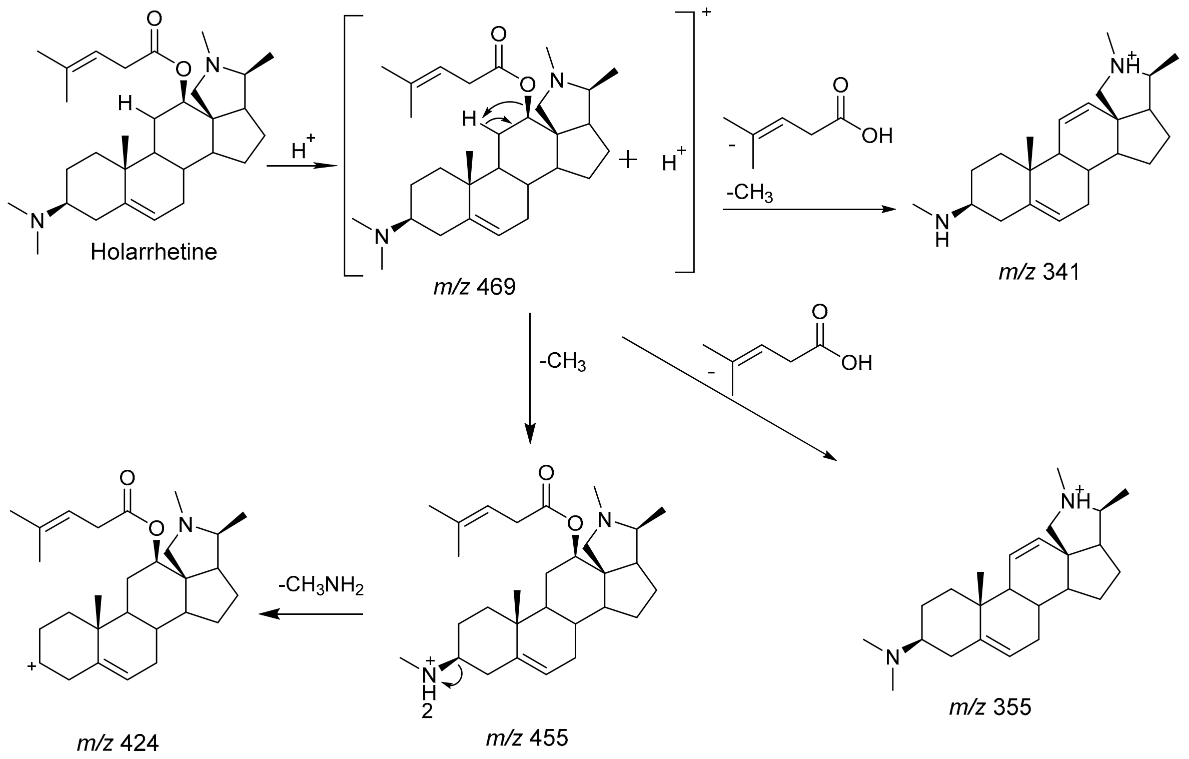

Scheme S9 : MS/MS fragmentation of holarrhetine (**10**)

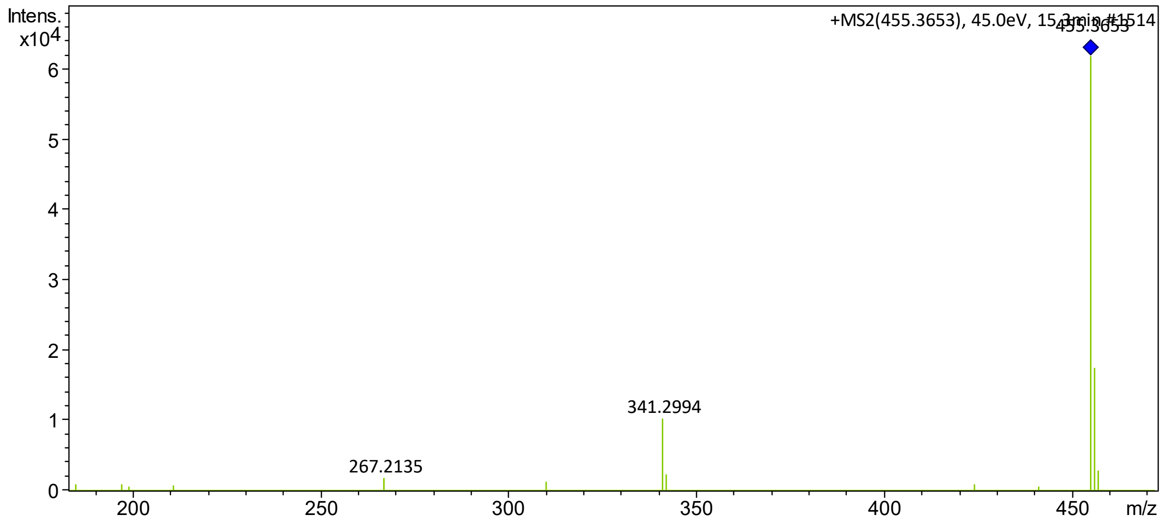

Figure S49: MS/MS spectrum of holarrhesine (**11**)

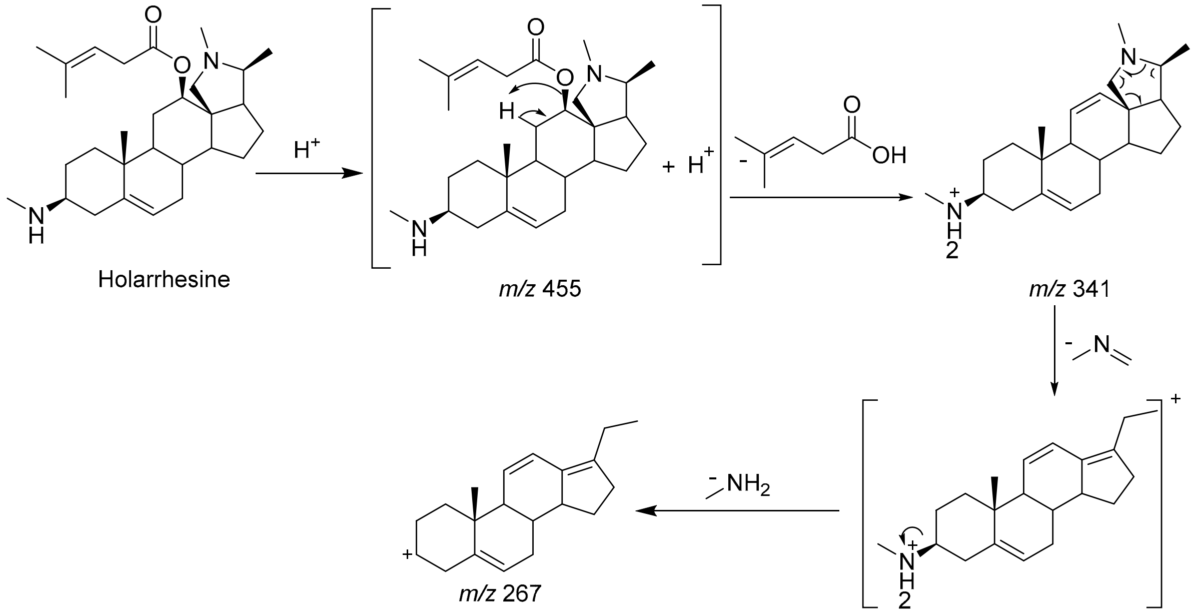

Scheme S10 : MS/MS fragmentation of holarrhesine (**11**)

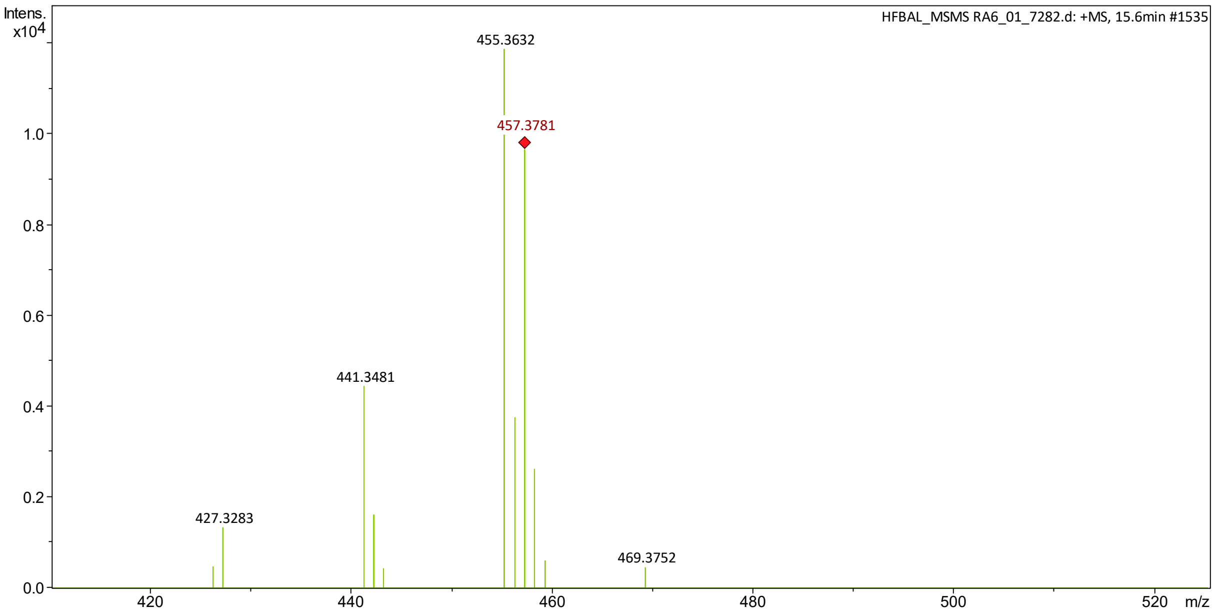

Figure S50: MS/MS spectrum of solanopubamide B (**12**)

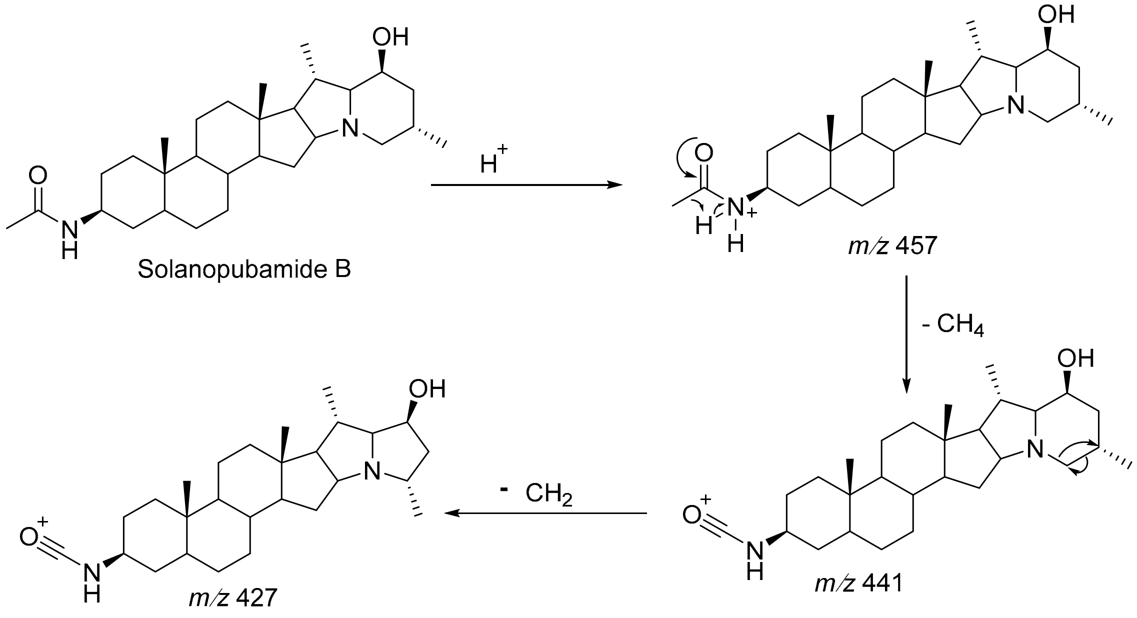

Scheme S11: MS/MS fragmentation of solanopubamide B (**12**)

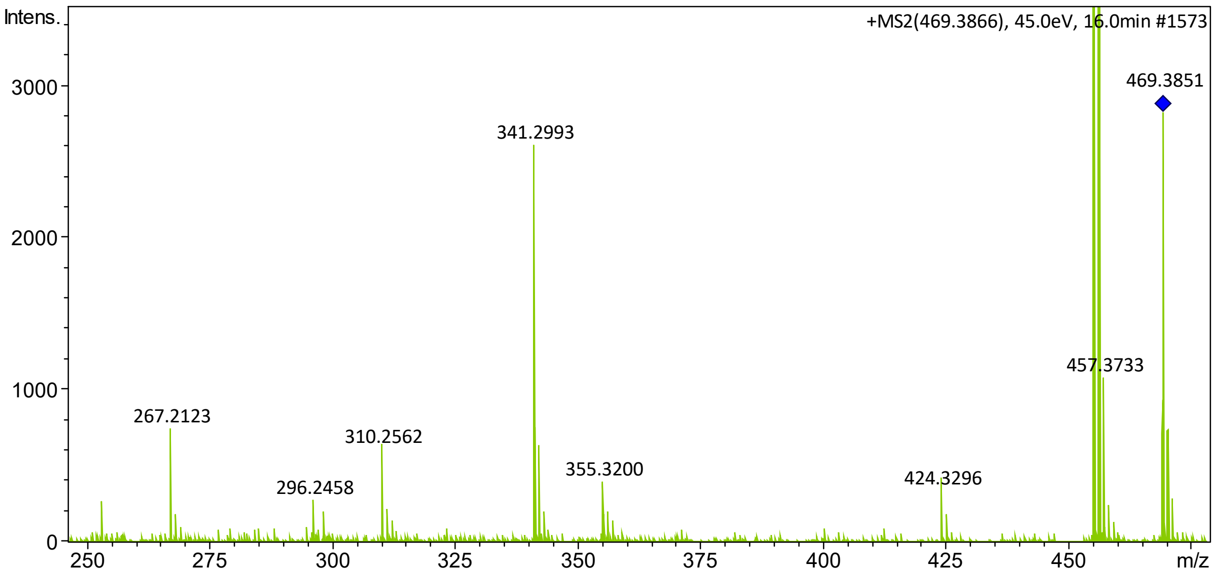

Figure S51: MS/MS spectrum of holarrhetine isomer (**13**)

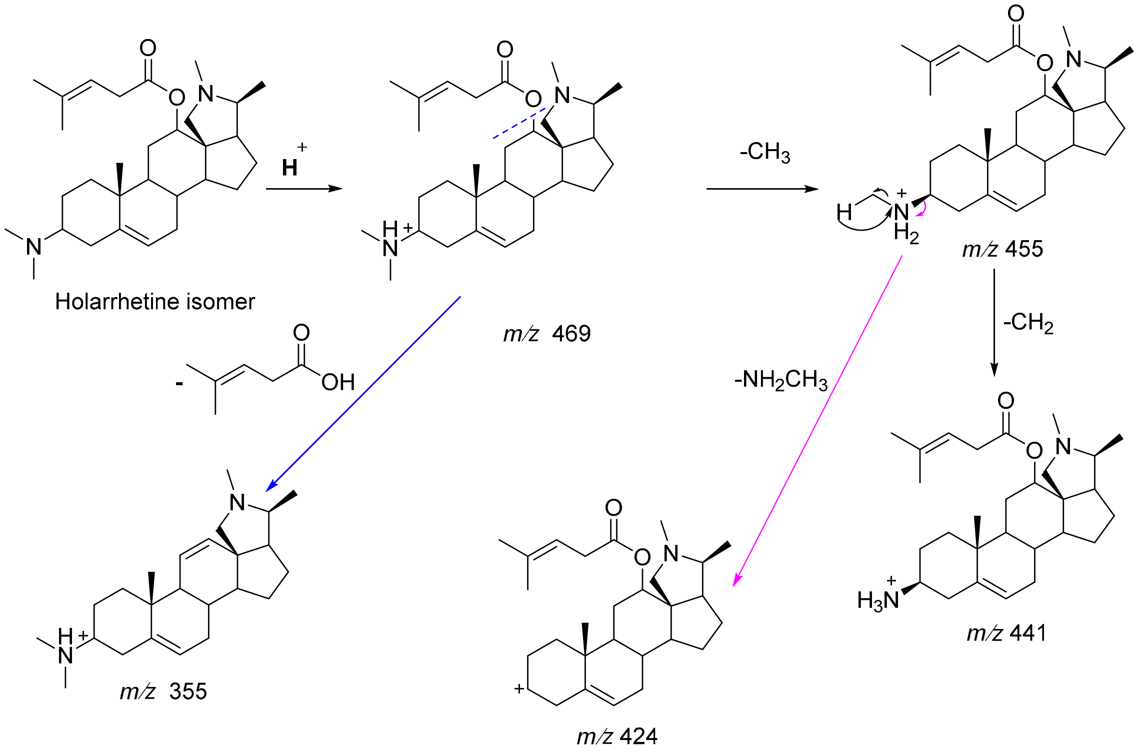

Scheme S12: MS/MS fragmentation of holarrhetine isomer (**13**)

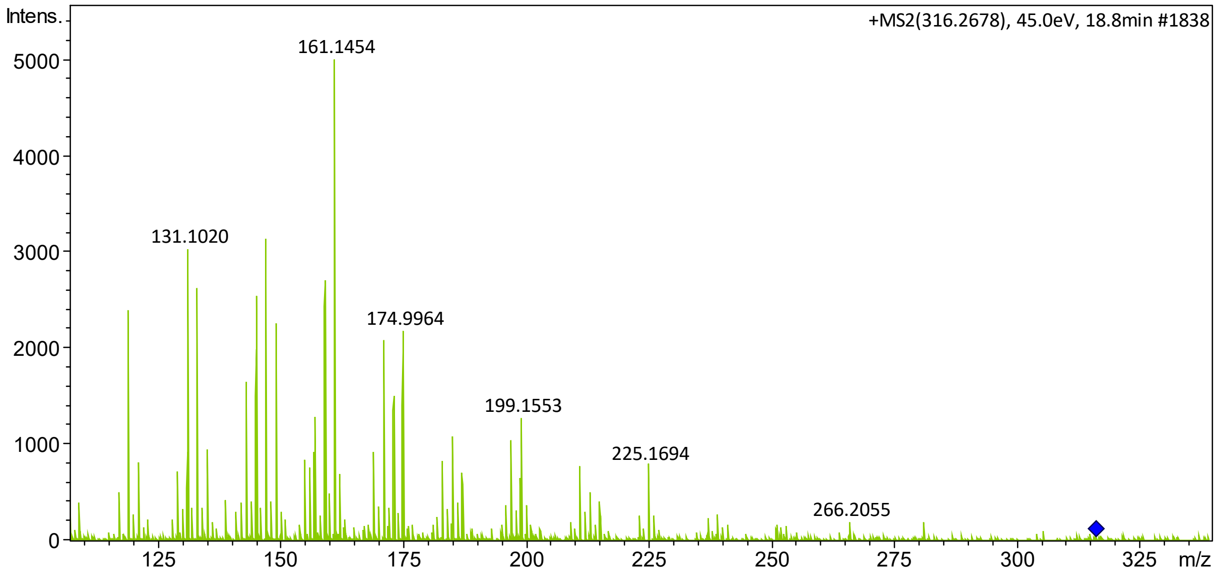

Figure S52 : MS/MS spectrum of holaphyllamine (**14**)

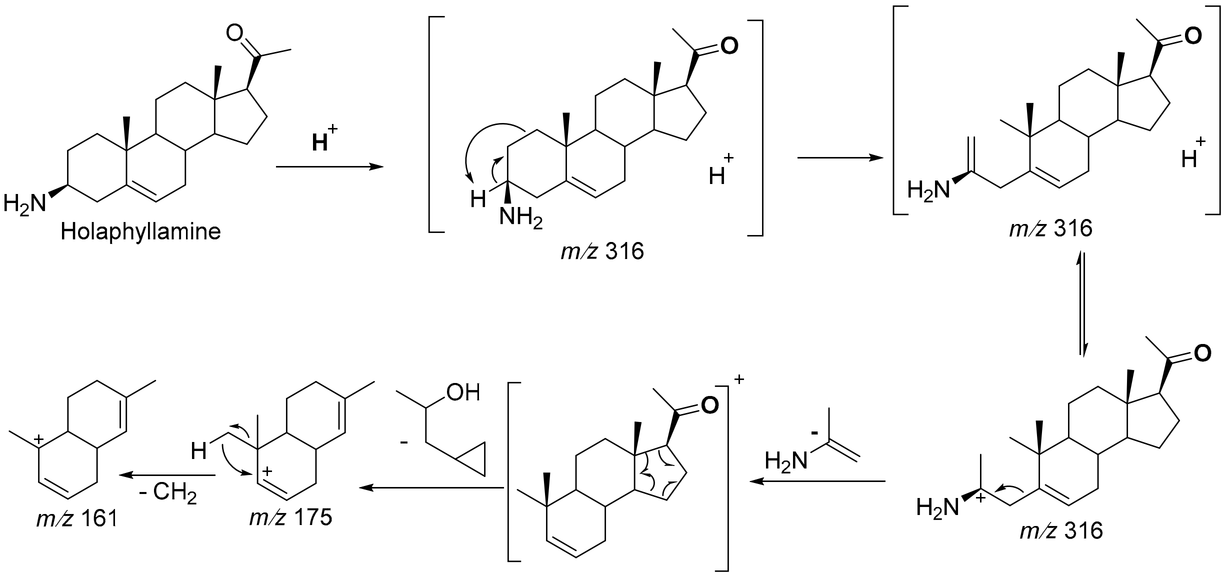

Scheme S13: MS/MS fragmentation of holaphyllamine (**14**)

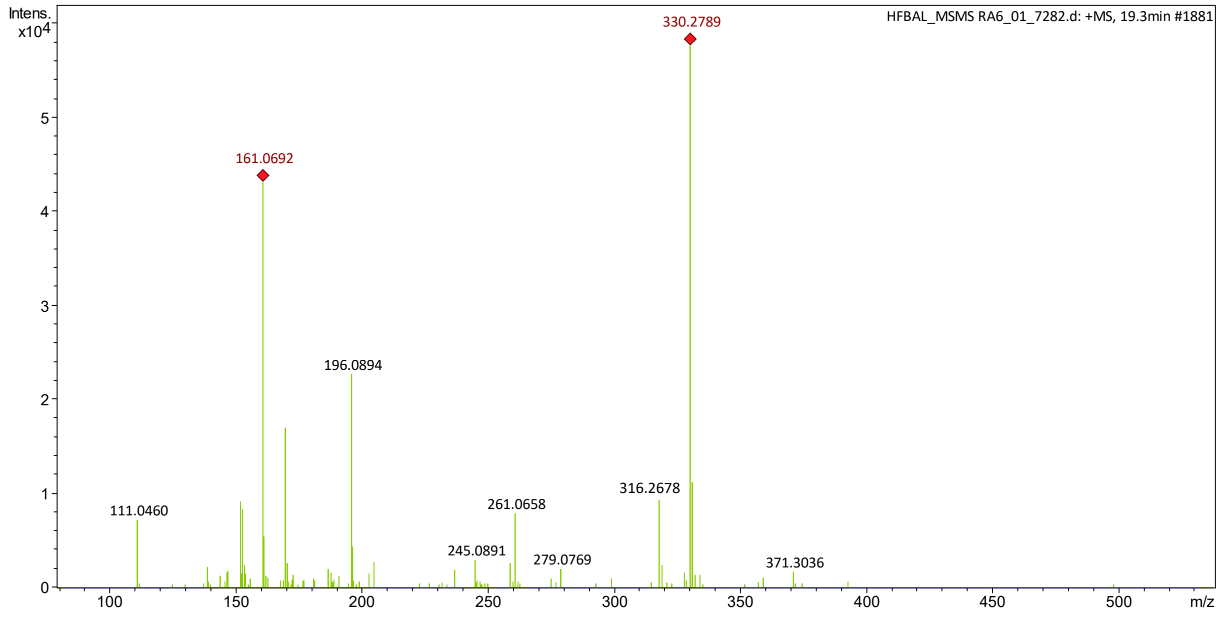

Figure S53: MS/MS spectrum of *N*-methylholaphyllamine (**16**)

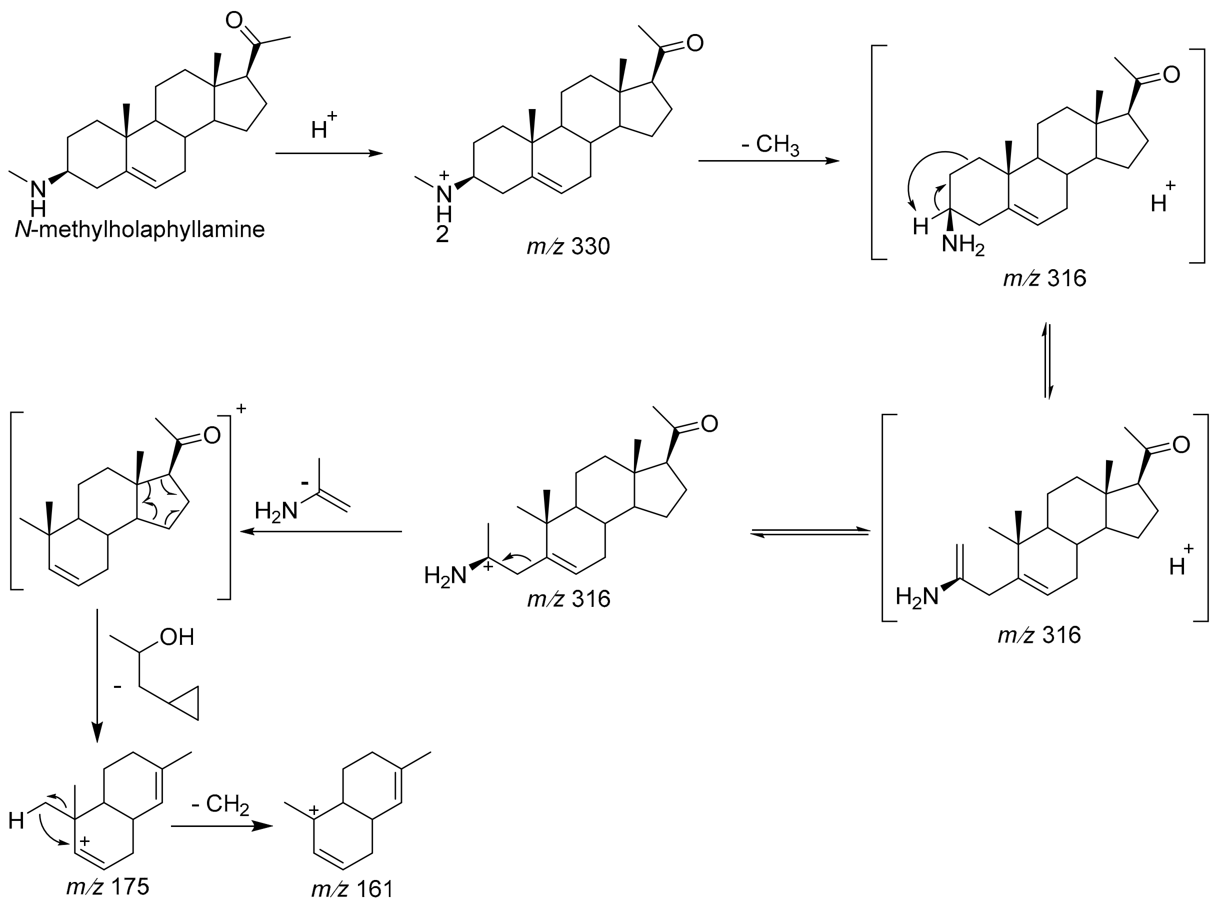

Scheme S14: MS/MS fragmentation of *N*-methylholaphyllamine (**16**)

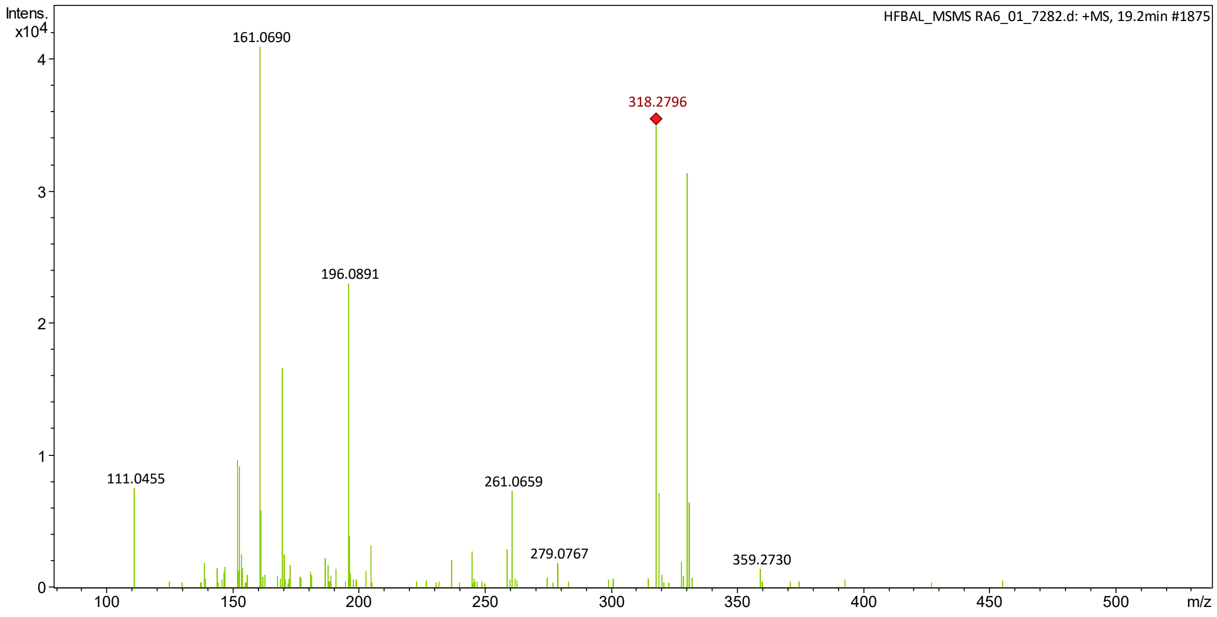

Figure S54: MS/MS fragmentation pattern of holaphyllaminol (**15**)

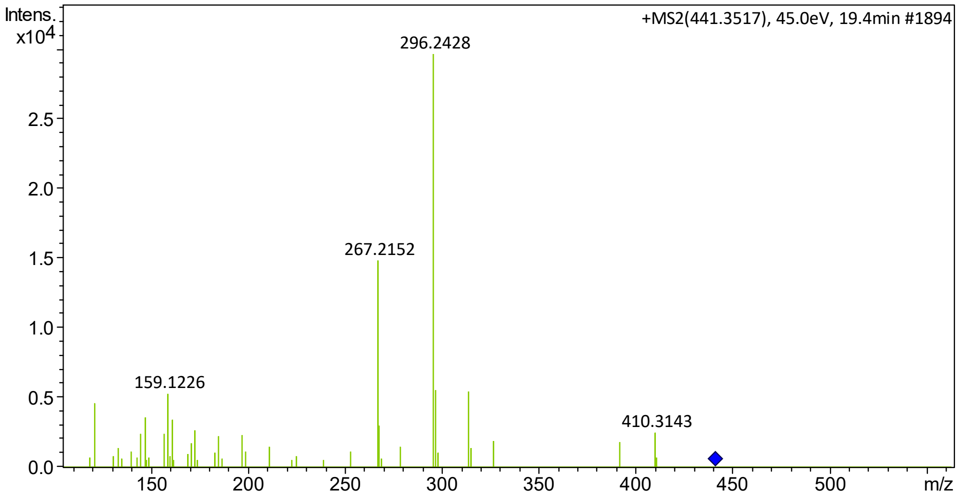

Figure S55: MS/MS fragmentation pattern of salignemamide D isomer (**17**)

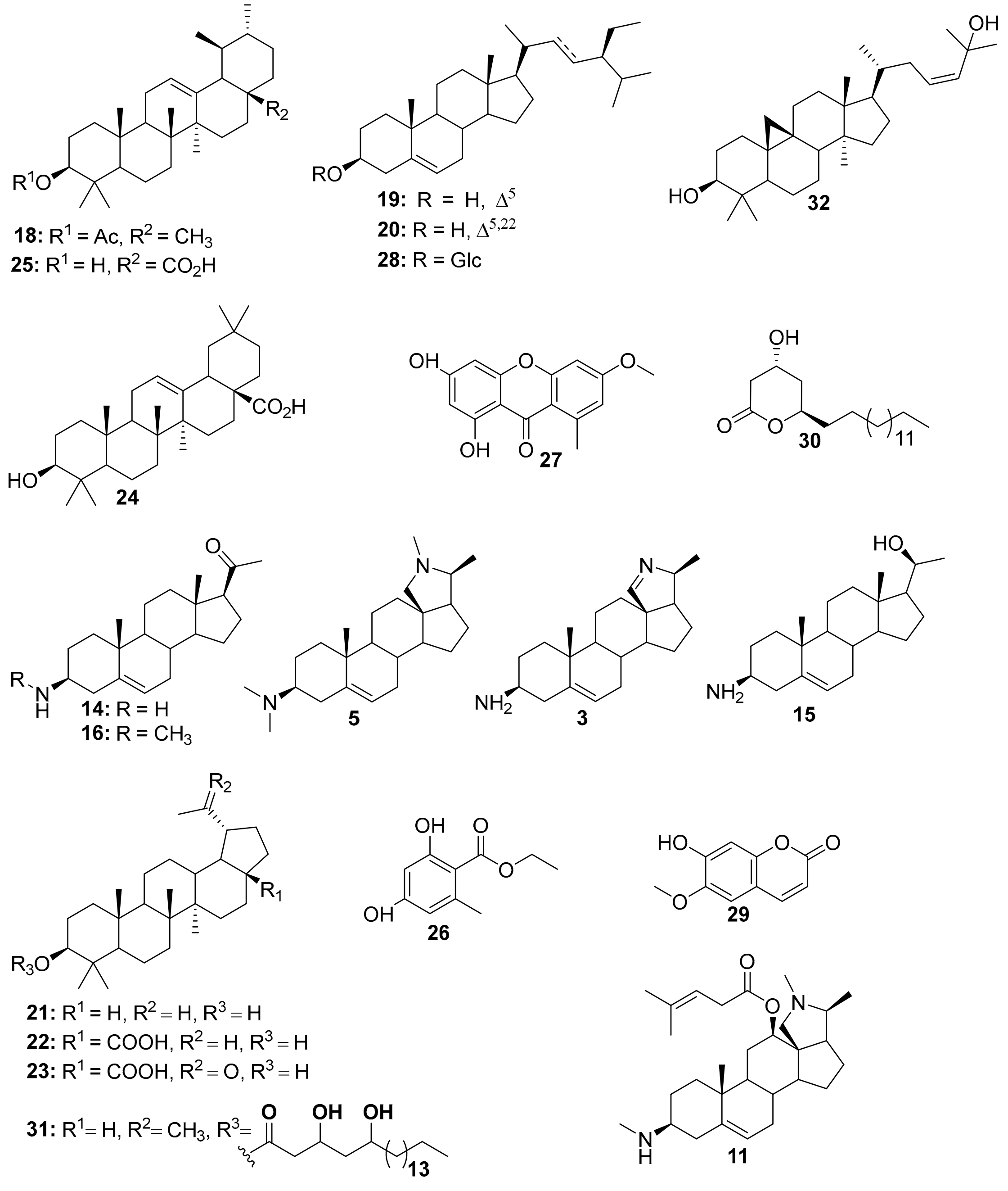

Figure S56: Structures of isolated compounds from the stem bark of *H. floribunda*
